# Tetrasubstituted Selenophenes from the Stepwise Assembly of Molecular Fragments on a Diiron Frame and Final Cleavage of a Bridging Alkylidene

**DOI:** 10.1021/acs.inorgchem.0c02748

**Published:** 2020-11-18

**Authors:** Giacomo Provinciali, Marco Bortoluzzi, Tiziana Funaioli, Stefano Zacchini, Beatrice Campanella, Guido Pampaloni, Fabio Marchetti

**Affiliations:** † Dipartimento di Chimica e Chimica Industriale, Università di Pisa, Via G. Moruzzi 13, I-56124 Pisa, Italy; ‡ Dipartimento di Scienze Molecolari e Nanosistemi, Ca’ Foscari Università di Venezia, Via Torino 155, I-30170 Mestre (VE), Italy; § Dipartimento di Chimica Industriale “Toso Montanari”, Università di Bologna, Viale Risorgimento 4, I-40136 Bologna, Italy; ∥ Istituto di Chimica dei Composti Organometallici, Consiglio Nazionale delle Ricerche, Via G. Moruzzi 1, I-56124 Pisa, Italy

## Abstract

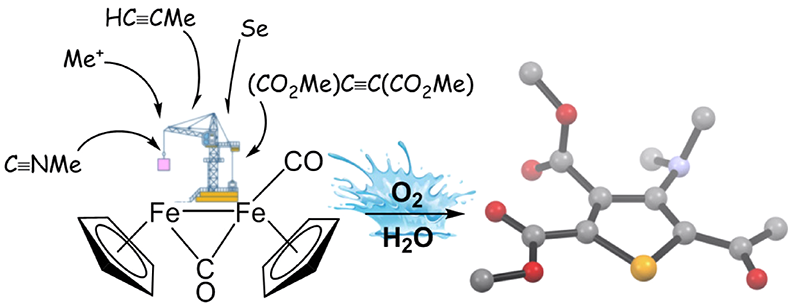

A series of 2,3-dicarboxylato-5-acetyl-4-aminoselenophenes, **5a**–**j**, was obtained via the uncommon assembly of building blocks on a diiron platform, starting from commercial [Fe_2_Cp_2_(CO)_4_] through the stepwise formation of diiron complexes [**2a**–**d**]CF_3_SO_3_, **3a**–**d**, and **4a**–**j**. The selenophene-substituted bridging alkylidene ligand in **4a**–**j** is removed from coordination upon treatment with water in air under mild conditions (ambient temperature in most cases), affording **5a**–**j** in good to excellent yields. This process is highly selective and is accompanied by the disruption of the organometallic scaffold: cyclopentadiene (CpH) and lepidocrocite (γ-FeO(OH)) were identified by NMR and Raman analyses at the end of one representative reaction. The straightforward cleavage of the linkage between a bridging Fischer alkylidene and two (or more) metal centers, as observed here, is an unprecedented reaction in organometallic chemistry: in the present case, the carbene function is converted to a ketone which is incorporated into the organic product. DFT calculations and electrochemical experiments were carried out to give insight into the release of the selenophene-alkylidene ligand. Compounds **5a**–**j** were fully characterized by elemental analysis, mass spectrometry, IR, and multinuclear NMR spectroscopy and by X-ray diffraction and cyclic voltammetry in one case.

## Abstract 2

Metal−metal cooperativity in action! Different fragments are combined on the {Fe_2_Cp_2_(CO)_2_} skeleton to give highly functionalized selenophene ligands, linked to the iron centers through a bridging alkylidene, which is easily removed from coordination by exposure to air/water in ethereal solution.

## Introduction

Stoichiometric organometallic reagents based on transition metals display advantageous features compared to related main group compounds, and thus have been successfully employed for the development of valuable synthesis strategies and the construction of structural motifs not accessible from typical organic reactions, either metal-catalyzed or not.^1^ A striking example is given by monochromium complexes with a Fischer alkylidene ligand of general formula [Cr(CO)_5_{=C(OR)R′}], which have emerged as versatile stoichiometric reagents for multicomponent cyclization reactions, providing access to highly functionalized organic molecules incorporating the alkylidene moiety.^1a,1b,2^ This approach has been largely exploited for the preparation of a variety of fine chemicals (e.g., pharmaceuticals and natural products).^2a,3^ The unique combination of the favorable properties of iron (i.e., its abundance, low cost, and nontoxicity) renders easily available iron compounds ideal candidates for the development of metal-directed processes.^4,5^ In particular, Collman’s reagent (i.e., the Na_2_[Fe(CO)_4_] salt) has been regarded as a transition-metal analogue of the Grignard reagents and is effective in converting alkyl halides to aldehydes/ketones.^6^

Diiron bis-cyclopentadienyl complexes hold an enhanced potential, with respect to related monoiron species, in the assembly of organic fragments.^7^ This is essentially due to the cooperative effects supplied by the two adjacent metal centers working in concert and the availability of bridging coordination sites enabling peculiar reactivity patterns.^8,9^ Starting from the easily available and inexpensive [Fe_2_Cp_2_(CO)_4_] (Cp = η^5^-C_5_H_5_) and via the preliminary replacement of one CO (either by chemical or photochemical methods), it is possible to obtain a huge diversity of hydrocarbyl ligands.^7,10^ Usually, the latter are firmly anchored to the two irons by means of bridging carbyne or carbene atoms, and their removal from coordination is hardly viable even at high temperatures.^11^ More generally, the one-pot extrusion of alkylidene ligands of the Fischer type may be achieved from monometal complexes using various strategies^12^ including hydrolysis;^13^ otherwise, this is an exceedingly rare process when the carbene is bridged between two or more metal centers.^14^ After a careful evaluation of the literature, we became aware of only one example of elimination of a *bridging* alkylidene ligand from late transition-metal complexes obtained from the unclean reaction of a triosmium carbonyl cluster with excess CF_3_CO_2_H at 90 °C.^15^

**Scheme 1:**
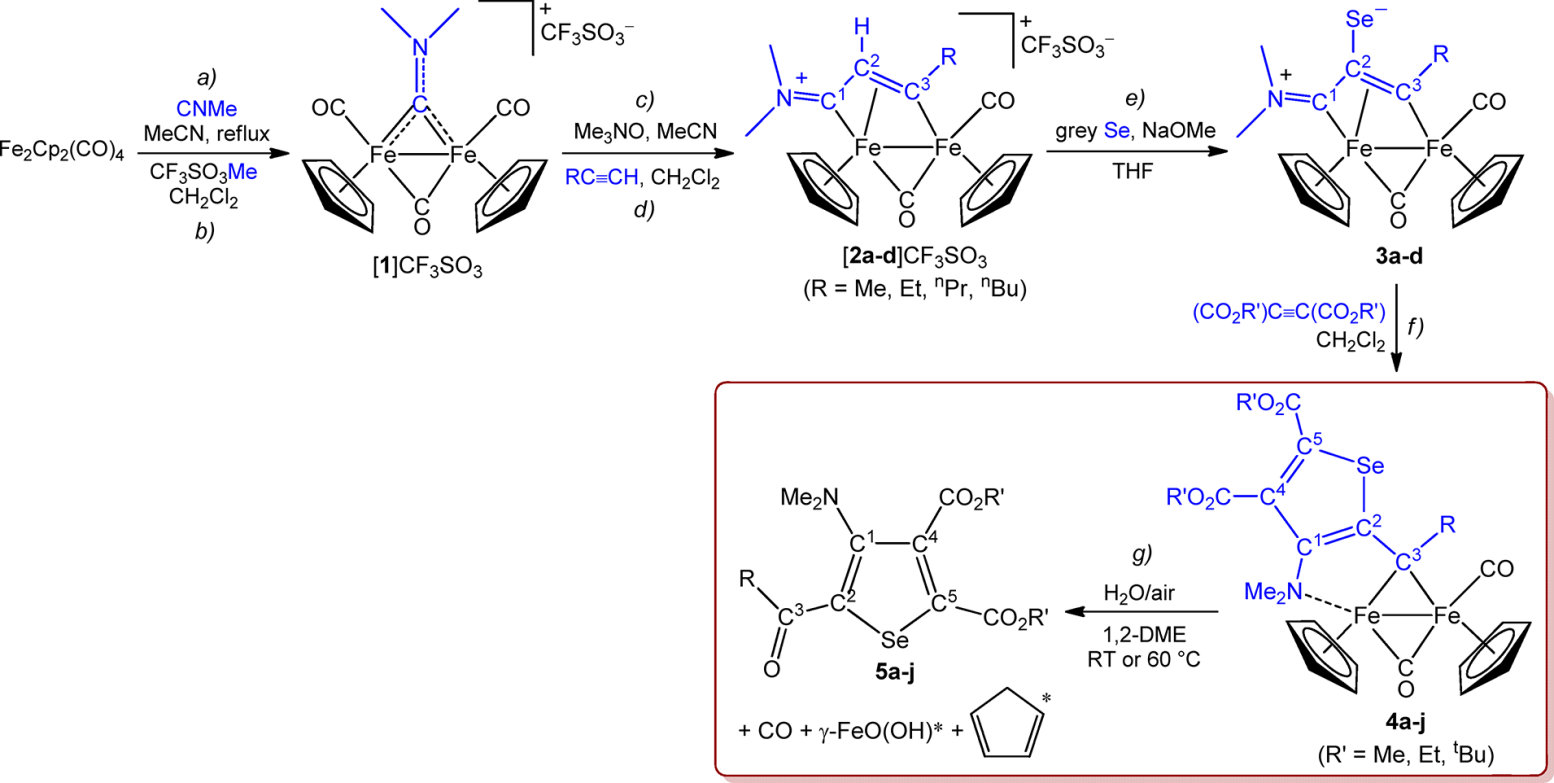
Stepwise Assembly of Molecular Pieces (in Blue) To Build a Selenophene-μ-alkylidene Ligand (Steps a–f) and Subsequent Demetallation (Step g) (*) Detected in the reaction leading to **5a**.

In the last 15 years, some of us have been deeply involved with the versatile chemistry of diiron μ-vinyliminium complexes ([**2a**–**d**]CF_3_SO_3_ in Scheme 1), which can be expeditiously prepared on the gram/multigram scale from [Fe_2_Cp_2_(CO)_4_].^7a,7b,16^ In the framework of our interest in the development of bis-cyclopentadienyl diiron complexes as potential anticancer drugs^17^ and on account of the relevance of the selenophene moiety in medicinal chemistry,^18^ we exploited the derivatization potential of the vinyliminium ligand in preparing selenophene-decorated ligands, connected to the two iron atoms through a bridging Fischer alkylidene carbon (**4a**–**j** in Scheme 1). In view of the biological studies, we preliminarily probed the stability of **4a** in the presence of water and, surprisingly, in this condition we observed the facile release of the functionalized ligand. The demetallation reaction consists in the cleavage of the Fe_2_-alkylidene linkage by the synergic action of air (O_2_) and water, and provides an entry into a new class of highly functionalized selenophenes including a β-amino substituent. Note that functionalized selenophenes may display notable properties and find potential applications in material chemistry^19^ and medicine,^18^ and many synthetic procedures have been reported to access a wide variety of this class of organic compounds.^20^ Differently to the present method, the source for the selenium center is often a selenium compound (e.g. Na_2_S, SeO_2_, SeCl_2_, KSeCN) and most rarely elemental selenium.^20,21^ Moreover, the synthesis of amino-substituted selenophenes is usually not a trivial task,^22^ and selenophenes bearing an amino substituent in β-position are quite rare and available only through elaborated procedures.^22b,23^

## Results and Discussion

### Synthesis and Characterization of Compounds

Selenophenes were obtained through a multi-step synthesis starting from [Fe_2_Cp_2_(CO)_4_] (Scheme 1). Thus, vinyliminium complexes [Fe_2_Cp_2_(CO)(μ-CO){μ–η^1^:η^3^-C^3^(R)=C^2^HC^1^=NMe_2_}]CF_3_SO_3_ (R = Me, [**2a**]CF_3_SO_3_; R = Et, [**2b**]CF_3_SO_3_; R = ^n^Pr, [**2c**]CF_3_SO_3_; R = ^n^Bu, [**2d**]CF_3_SO_3_) were prepared in gram scale in excellent yields, from the aminocarbyne precursor [**1**]CF_3_SO_3_ according to the literature (steps c-d).^24^ Compounds [**2b**]CF_3_SO_3_ and [**2c**]CF_3_SO_3_ are novel, while [**2a**]CF_3_SO_3_^17^ and [**2d**]CF_3_SO_3_^25^ were already reported. Salient IR and NMR features of [**2b**–**c**]CF_3_SO_3_ are in alignment with those ones previously described for such class of compounds.^26^ The dehydrogenative selanylation^27^ of [**2a**–**d**]CF_3_SO_3_ to afford the zwitterionic derivatives [Fe_2_Cp_2_(CO)(μ-CO){μ–η^1^:η^3^-C^3^(R)C^2^(Se)C^1^NMe_2_}] (R = Me, **3a**; R = Et, **3b**; R = ^n^Pr, **3c**; R = ^n^Bu, **3d**) exploits the previously documented acidity of the C^2^–H hydrogen (step e); here, this reaction has been performed using a modified, optimized literature procedure under inert atmosphere.^28^ The new compounds **3b**–**c** were fully characterized by means of IR and NMR (^1^H, ^13^C, ^77^Se) spectroscopy. The selenium nucleus resonates in the range 130-150 ppm. The successive cyclization reaction with alkynes generates the selenophene moiety (step f), which is tethered to the diiron frame via coordination with a nitrogen (belonging to a tertiary amine group) and a carbon atom (bridging alkylidene). This reaction was known for **3a** leading to **4a** (R = R′ = Me),^29^ and here is extended to a series of new compounds, **4b**–**j**. In general, the IR spectra of **4a**–**j** (in CH_2_Cl_2_) display the bands due to the carbonyl ligands at about 1930 (terminal CO) and 1745 cm^–1^ (bridging-CO). In the NMR spectra, the *N*-methyls are non-equivalent, the rotation around the C^1^–N bond being inhibited by N-coordination to one iron center [e.g. in the case of **4b** (R = Me, R′ = Et): δ(^1^H) = 2.49, 2.05 ppm; δ(^13^C) = 59.6, 52.0 ppm]. The bridging carbon C^3^ manifests its alkylidene nature with the ^13^C resonance falling around 185 ppm. The ^77^Se spectrum of **4a**–**j** consists of a signal in the range 480–500 ppm. For sake of comparison, the selenium atom in unsubstituted selenophene resonates at 613 ppm (CDCl_3_ solution).^30^ The structure of **4e** (R = R′ = Et) was confirmed by a single crystal X-ray diffraction analysis (Figure S1), resembling that previously described for **4a**. The annulation reaction affording **4a**–**j** seems circumscribed to di(mono)alkyl-acetylenedicarboxylates,^29^ and does not work with other alkynes such as HC≡CH, (CF_3_)C≡C(CF_3_), MeC(O)C≡CH and HC≡C[3,5-C_6_H_3_(CF_3_)_2_].

Details on the preparation and the characterization of [**2a**–**d**]CF_3_SO_3_, **3a**–**c** and **4a**–**j** are supplied in the Supporting Information.

As explained in the Introduction, the unprecedented and unexpected reactivity of complexes **4a**–**j** with water/air (inside the red rectangle in Scheme 1) was serendipitously discovered, in the attempt to examine the stability of the complexes in aqueous media in view of potential biological applications. Following treatment of a dichloromethane solution of **4a** with a large excess of H_2_O (1-2 mL vs. ca. 0.3 mmol of **4a**), IR spectroscopy indicated the complete disappearance of the CO ligands after 24 h, and the selenophene **5a** (R = R′ = Me) was subsequently isolated in a low yield (5-10%) after filtration of the reaction mixture through alumina. A screening of possible reaction conditions outlined that ethereal solvents (e.g. THF, diethyl ether) lead to significantly higher yields of selenophene, and aerated 1,2-dimethoxyethane(1,2-dme)/H_2_O mixture resulted the optimal reaction medium. The favorable role of air (O_2_) was suggested by the observation that solid samples of **4e** and **4i** decomposed with gas release (CO) after being stored for 4-8 weeks in contact with air inside sealed vials (ca. 20 mg of each compound in a volume of ca. 5 mL). It has to be noted that both ionic and neutral diiron complexes analogous to **4a**–**j** and containing different bridging hydrocarbyl ligands may be water sensitive but are indefinitely air stable in the solid state.^17a−17d^ As for **5a**, **5b**–**j** were isolated after work-up in 48-97% yields from the parent diiron species **4b**–**j**: the reactions in aerated 1,2-dimethoxyethane generally proceeded to completion at ambient temperature in 24 h, except those leading to **5f**–**h** which needed a gentle heating of the mixtures. The oxidative dissociation process is highly selective, in that selenium products different from **5a**–**j** were not found. The destiny of the {Fe_2_Cp_2_(CO)_2_} scaffold was elucidated in the case of the formation of **5a** (details in the Experimental Secction). Thus, cyclopentadiene (CpH) was detected by a ^1^H NMR experiment in THF-*d*^8^ solution in 2:1 ratio with respect to **5a**, as expected for a selective fragmentation process. Besides, the inorganic solid precipitated from the 1,2-dme solution was analyzed by Raman spectroscopy and thus identified as lepidocrocite, γ-FeO(OH) (Scheme 1 and Figure S2).

In summary, we report the synthesis of a novel class of tetrasubstituted selenophenes, **5a**–**j**, with a set of substituents which is unprecedented in the literature, generated from the unusual combination of building blocks on a diiron frame (Scheme 1: one isocyanide, one methyl group, two alkynes, and one Se atom). In general, in classic organic synthesis, the source of the selenium center is a selenium compound and not elemental selenium (Introduction), and that of the amino group is an amine and not an isocyanide.^22^

Remarkably, the synthesis of aminocarbyne complex [**1**]CF_3_SO_3_ efficiently works up to the 15–20 g scale, and that of vinyliminium complex **2** up to the 5–10 g scale.

**Figure 1:**
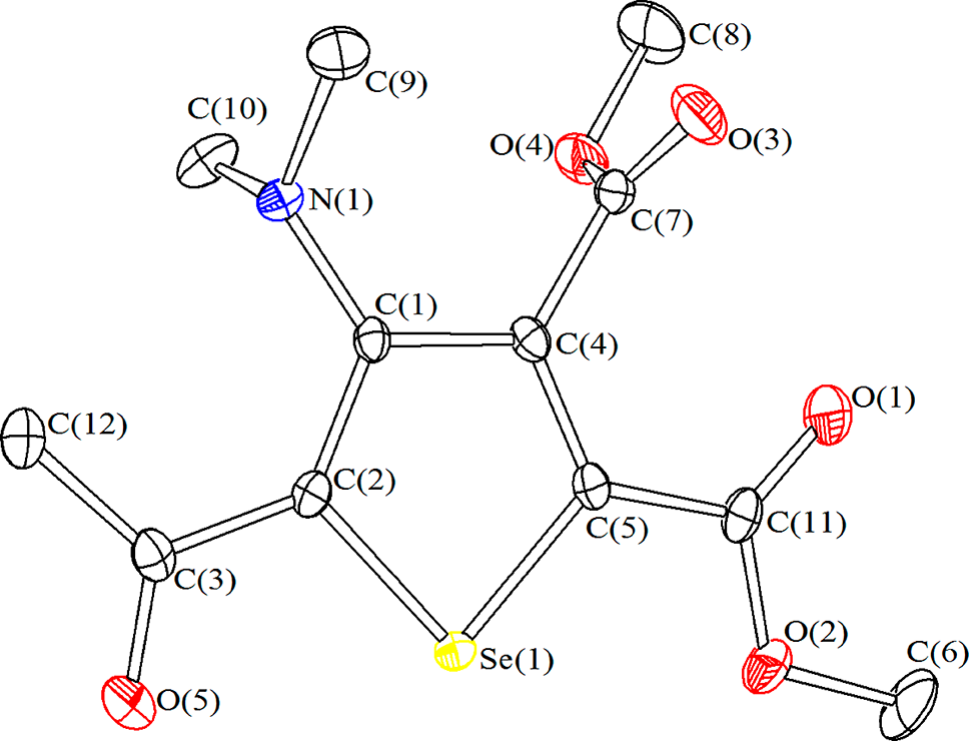
Molecular structure of SeC{C(O)Me}C(NMe_2_)C(CO_2_Me)C(CO_2_Me) (**5a**) with labeling. Displacement ellipsoids are at the 50% probability level. H atoms have been omitted for clarity. Selected bond distances (Å) and angles (deg): C(5)–Se(1) 1.8595(16), C(2)–Se(1) 1.8653(16), C(5)–C(4) 1.368(2), C(1)–C(2) 1.387(2), C(4)–C(1) 1.439(2), C(4)–C(7) 1.505(2), C(5)–C(11) 1.479(2), C(2)–C(3) 1.480(2), C(1)–N(1) 1.414(2), C(11)–O(1) 1.209(2), C(11)–O(2) 1.334(2), C(6)–O(2) 1.453(2), C(7)–O(3) 1.197(2), C(7)–O(4) 1.339(2), C(8)–O(4) 1.452(2), C(3)–O(5) 1.227(2), C(3)–C(12) 1.499(2), Se(1)–C(5)–C(4) 113.00(11), C(5)–C(4)–C(1) 114.46(14), C(4)–C(1)–C(2) 113.26(14), C(1)–C(2)–Se(1) 112.73(11), C(2)–Se(1)–C(5) 86.55(7), C(2)–C(3)–O(5) 118.39(15), sum at C(11) 360.0(3), sum at C(7) 360.0(3), sum at C(3) 360.0(3), and sum at N(1) 346.7(3).

Compounds **5a**–**j** were obtained as crystalline solid materials from cooled pentane solutions and fully characterized by elemental analysis, mass spectrometry, IR (CH_2_Cl_2_ and solid state), and multinuclear NMR spectroscopy. Moreover, the molecular structure of **5a** was elucidated by X-ray diffraction (Figure 1). It is based on a selenophene ring which is perfectly planar [mean deviation from the C(5)C(4)C(1)C(2)Se(1) least-squares plane = 0.0049 Å], and also C(11), C(7), N(1), and C(3) lie in the same plane [maximum deviation from the plane = 0.1437 Å]. A similar situation was previously observed in SeC_4_(CO_2_Me)_4_.^31^ Alternating double and single C–C bonds are present in the ring of **5a**,^32^ thus C(5)–C(4) [1.368(2) Å] and C(1)–C(2) [1.387(2) Å] are considerably shorter than C(4)–C(1) [1.439(2) Å]. The C(5)–Se(2) [1.8595(16) Å] and C(2)–Se(1) [1.8653(16) Å] distances are as expected for single C(sp^2^)–Se bonds. Carboxylic and carbonylic centers C(11), C(7), and C(3) display a perfect sp^2^ hybridization [sum angles are 360.0(3)°], whereas N(1) presents a considerable pyramidilization [sum angle at N(1) 346.7(3)°]. In agreement with this, the C(1)–N(1) contact [1.414(2) Å] is essentially a single bond. The angle between the least-squares plane of the CO_2_Me group bonded to C(4) in **5a** and the plane of the selenophene ring is 83.0°.

**Figure 2:**
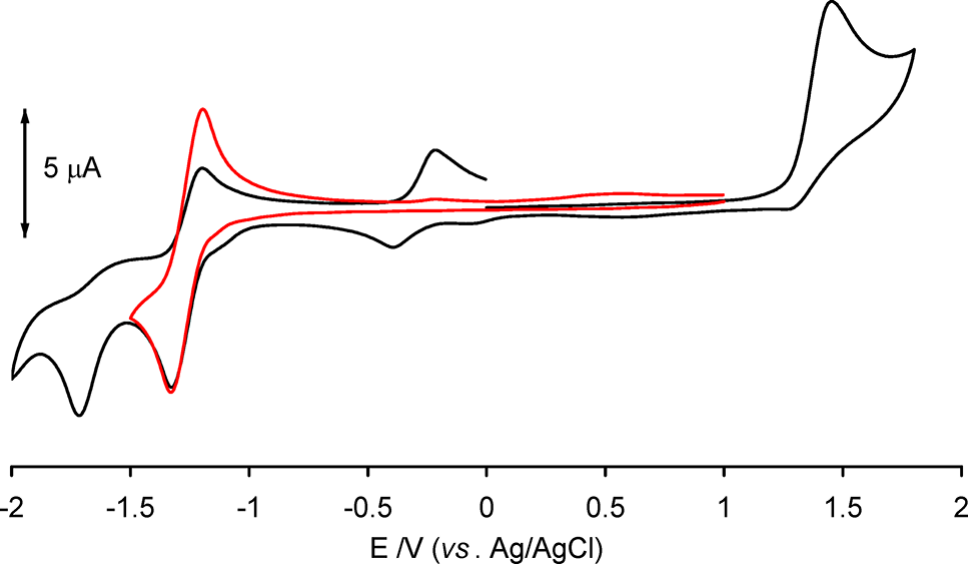
Voltammetric profiles of **5a** at a platinum electrode in 0.2 M 1,2-dme/[N^n^Bu_4_]PF_6_. (Black line) VC between +1.5 and −2.5 V. (Red line) VC between +0.5 and −2.0 V. Scan rate 0.1 V s^–1^.

The NMR spectra of **5a**–**j** (in CDCl_3_) exhibit single resonances for the {NMe_2_} group [e.g., at 2.87 ppm (^1^H) and 44.2 ppm (^13^C) for **5a**], indicating free rotation around the C^1^–N axis. The C^1^–C^4^ atoms, constituting the selenophene ring, resonate within the 136.6–154.1 ppm interval, as expected for alkene carbons. The selenium center was detected by ^77^Se NMR, occurring as a singlet at 584.4–591.7 ppm, thus evidencing some deshielding with respect to the situation found in **4a**–**j** (see above). The IR spectra of **5a**–**j** (solid state) comprise the absorption due to the ketonic C^3^=O function, which falls in the range of 1635–1646 cm^–1^. As a representative compound of the series, **5a** was also characterized by cyclic voltammetry (Figure 2 and Table S2). Thus, **5a** dissolved in 1,2-dimethoxyethane undergoes an irreversible oxidation at *E*_a_ = +0.91 V versus FeCp_2_ and two reductions. The analysis of the cyclic voltammetric response at different scan rates (between 0.02 and 2 V s^–1^) confirms that the redox change at *E*°′ = −1.79 V is an electrochemically quasi-reversible and chemically reversible one-electron process, while that at −2.25 V is irreversible and is associated with the appearance of the irreversible oxidation peak at −0.75 V. A CV of between +0.5 and −2.0 V attributed the peaks at −0.62 and −0.93 V to decomposition products arising from the irreversible oxidation.

### DFT Calculations and Electrochemical Studies

**Figure 3:**
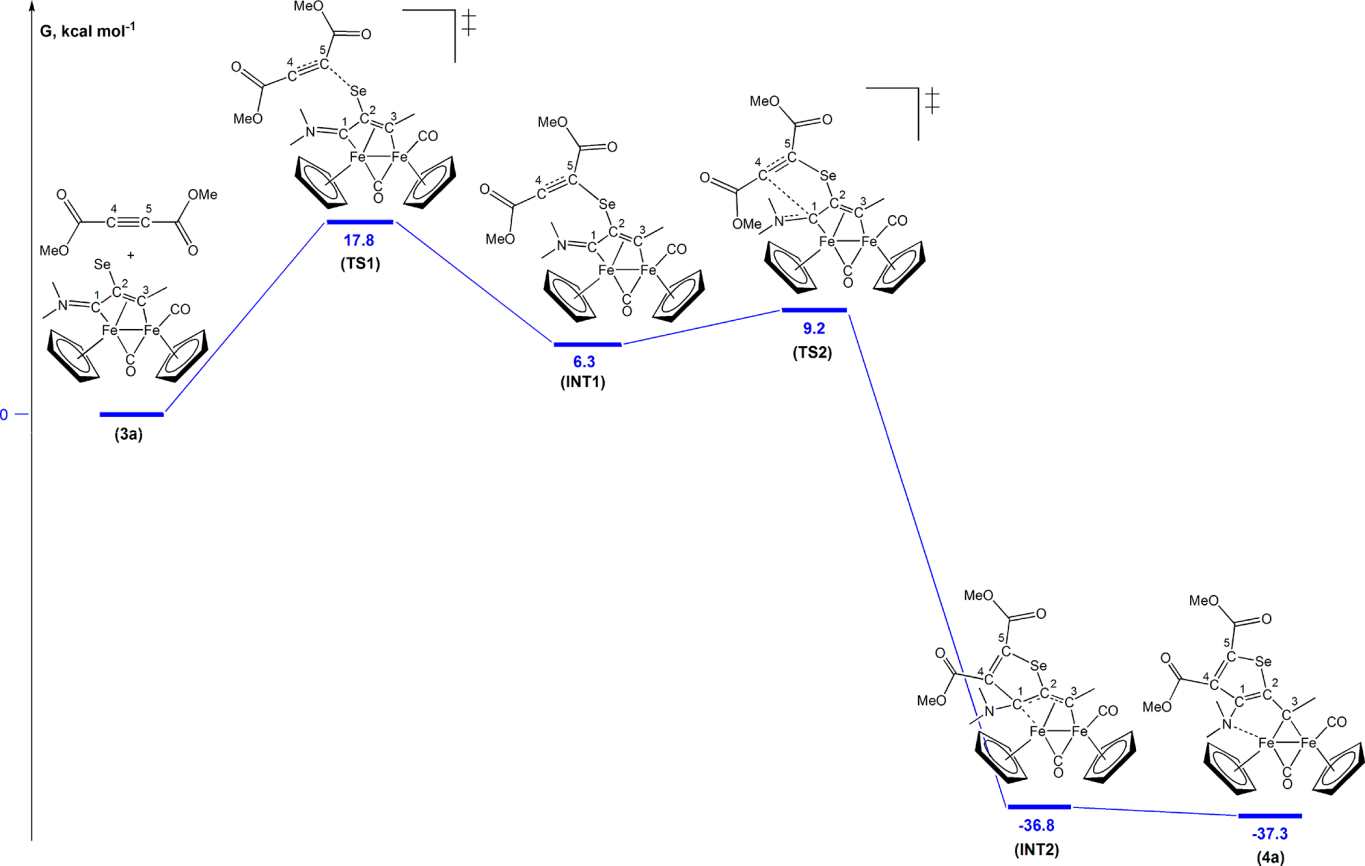
Computed mechanism for the reaction of **3a** (green) with dimethyl acetylenedicarboxylate to obtain **4a**. C-PCM/ωB97X/def2-SVP calculations, with chloroform as a continuous medium.

To the best of our knowledge, the straightforward de-coordination of the alkylidene ligand in **4a**–**j** represents a novelty in the landscape of the largely investigated chemistry of bridging alkylidene ligands coordinated to low-valence transition metals (Fischer alkylidene ligands). A parallel computational and electrochemical investigation was carried out in order to shed some light on the mechanism of such an unusual transformation, and the reaction leading from **3a** to **5a** was selected as a model one. First, starting from the DFT-optimized geometry of **3a** (Figure S3), the reaction with dimethyl acetylenedicarboxylate was computer-simulated (Figure 3). It is documented in the literature that acetylene dicarboxylates are prone to cyclization couplings due to their electron deficiency.^33^ The initial attack of the selenium on the [C≡C] bond gives **INT1** (Figure S4), whose relative energy is 6.3 kcal mol^–1^ higher than the sum of the reactants, through the transition state **TS1** (Figure S5, unique imaginary frequency i151 cm^–1^). The electron density on the alkyne carbon atoms is meaningfully increased in **INT1**, as highlighted by the variation of Mulliken charges (from −0.036 a.u. in dimethyl acetylenedicarboxylate to −0.177 and −0.355 a.u., respectively, for C^4^ and C^5^, in **INT1**). The delocalization of negative charge should favor the attack of C^4^ on the electron-poor C^1^ carbon (Mulliken charge = 0.084 a.u. in **INT1**), and in fact the transition state for the second step (**TS2**) has a low kinetic barrier of 2.9 kcal mol^–1^ (Figure S6, unique imaginary frequency i85 cm^–1^). The selenophene ring closure gives rise to a large variation in Gibbs free energy affording **INT2** (Figure S7), where the multidentate ligand is still anchored to the diiron frame through C^2^ and C^1^, other than the alkylidene carbon C^3^; subsequent ligand slippage affords final product **4a** (coordination via C^3^ and N). Overall, the mechanism depicted in Figure 3 suggests that the rate-determining step is the initial attack of the selenium on the alkyne.

Subsequently, the possible generation of **5a** from the hydrolysis of **4a** was evaluated by DFT; after many attempts, we did not find a plausible pathway for the direct attack of H_2_O on either alkylidene carbon C^3^ or the iron atoms of **4a** (Figure S8). The same is true when considering one molecule of solvent (1,2-dme) instead of H_2_O. For instance, the computed Gibbs energy variation for the formation of **4a-H**_**2**_**O** (Figure S9), from the nucleophilic attack of water on the alkylidene carbon C^3^, is +37.9 kcal mol^–1^ (C-PCM/ωB97X/def2-SVP calculations, H_2_O as the continuous medium).

**Figure 4:**
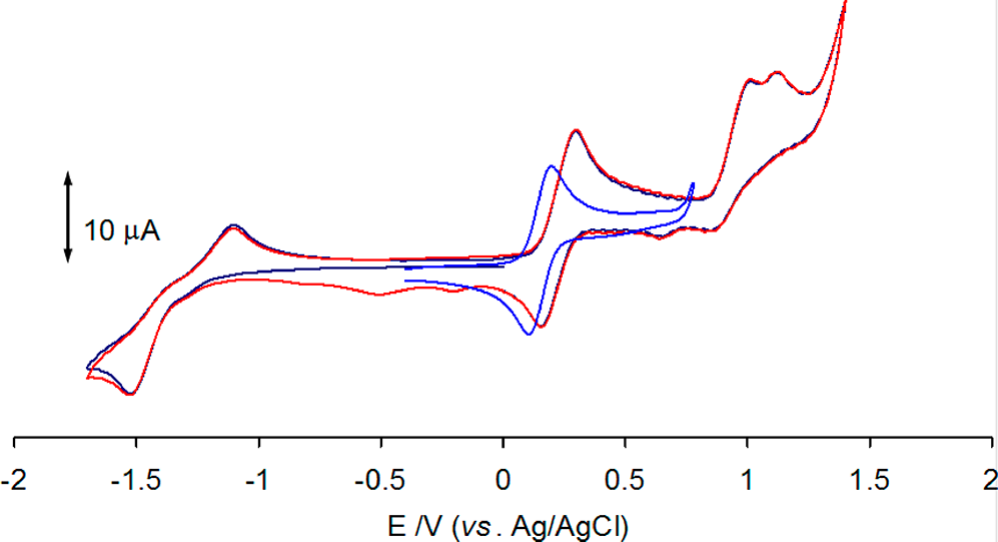
Voltammetric profiles of **4a** at a platinum electrode in 0.2 M 1,2-dme/[N^n^Bu_4_]PF_6_ solution. First cycle (black line) and second cycle (red line) of double-cycle voltammetry. Blue line, VC after the addition of water (14% V/V) to the 1,2-dme solution of **4a**. Scan rate 0.1 V s^–1^.

Since the formation of selenophenes via simple hydrolysis appears unlikely, we turned to consider the possible oxidative role of air by performing an electrochemical study. The cyclic voltammetry at a platinum electrode of **4a** in 1,2-dme containing [N^n^Bu_4_]PF_6_ as a supporting electrolyte is shown in Figure 4 and consists of one electrochemically quasi-reversible reduction at −2.06 V versus FeCp_2_ (peak-to-peak separation Δ*E*_p_ = 420 mV at 0.1 V s^–1^) and three oxidations (Table S2). The first oxidation occurs at the formal electrode potential *E*°′ = −0.31 V and is a one-electron, electrochemically and chemically reversible process on the time scale of CV. Instead, the further two oxidations, occurring respectively at *E*_a1_ = +0.58 V and *E*_a2_ = +0.47 V, are complicated by subsequent fast chemical reactions, as pointed out by the appearance of new reduction peaks at −0.74 V and −1.05 V during the back scan towards negative potentials, in the second cycle (red line) voltammetry of Figure 4.

We repeated the CV by adding water (14% V/V) to the 1,2-dme solution of **4a** (Figure 4, blue line): thus, we detected a 80 mV downshift of the first oxidation formal electrode potential (*E*°′ = −0.39 V), confirming that the presence of water favors the oxidation process. The chemical reversibility on the CV time scale did not change.

The number of electrons involved in the reversible oxidation was assessed by hydrodynamic voltammetry at a platinum rotating disk electrode before and after the addition of silver triflate to a solution of **4a**. The addition of 1 equiv of oxidizing agent per mole of complex was required in order to achieve complete oxidation, as indicated by the shift in the current/potential wave towards negative current values, according to the formation of the reducible [**4a**]^+^. We observed that the limiting current for the reduction of the newly generated [**4a**]^+^ slowly decreased, suggesting a limited stability of [**4a**]^+^ in 1,2-dme solution.

An *in situ* IR spectroelectrochemical experiment was conducted on a solution of **4a** in anhydrous 1,2-dme/[N^n^Bu_4_]PF_6_; a slow potential scan (1 mV/s) from −0.3 and +0.6 V (vs Ag) generated a sequence of IR spectra showing a shift of the terminal and bridging CO bands to higher wavenumber values (from 1931 and 1752 cm^–1^ to 1994 and 1837 cm^–1^, respectively), in agreement with the formation of [**4a**]^**+**^ (Figure S10a). During the backward reduction step (Figure S10b), **4a** was partially recovered (approximately 63% based on the peak areas in Figure S11) and new unidentified bands appeared at 2040 and 1819 cm^–1^.

**Figure 5:**
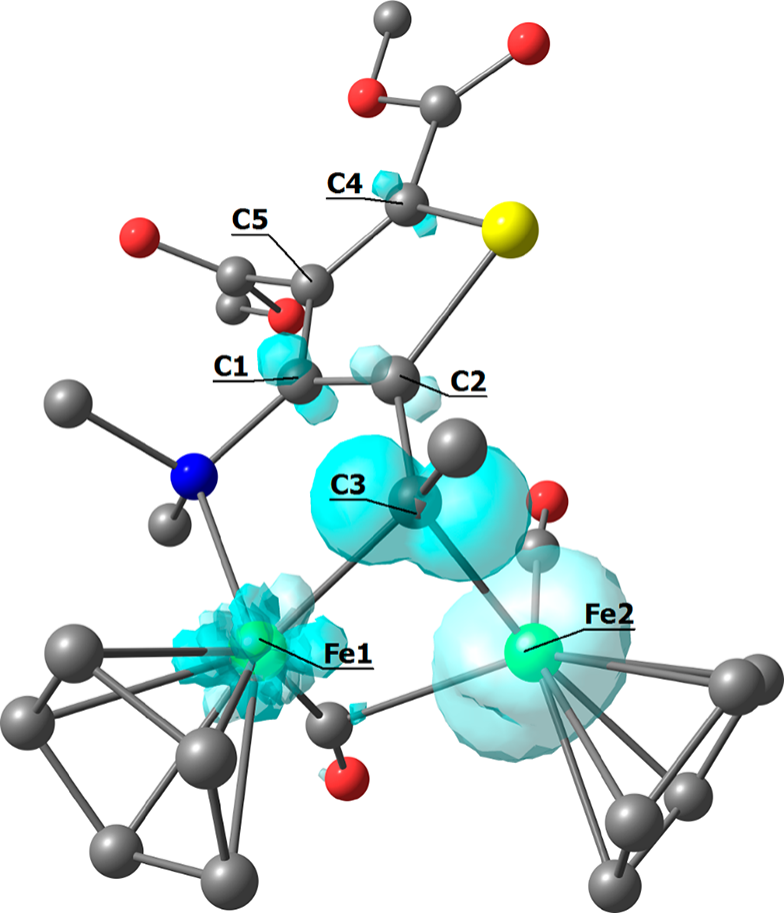
DFT-optimized structure of [**4a**]^+^ (C-PCM/ωB97X/def2-SVP, water as a continuous medium) and spin density plot (surface isovalue = 0.01 a.u.). Fe, green; Se, yellow; O, red; N, blue; and C, grey. Hydrogen atoms are omitted for clarity. Selected computed bond lengths (Å): Fe1–N 2.185; C1–N 1.449; C1–C2 1.371; C2–Se 1.864; C2–C3 1.471; C3–Fe1 2.048; and C3–Fe2 1.997. Selected Mulliken atomic spin densities (a.u.): Fe1 −0.007; Fe2 1.640; C1 −0.121; C2 0.133; C3 −0.461; C4 −0.085; C5 0.067; N 0.003; and Se −0.036.

The structure of [**4a**]^+^ was computationally optimized, and a view is given in Figure 5. The geometry of [**4a**]^+^ closely resembles that of **4a**, with the RMSD being only 0.378 Å, in accordance with the electrochemical reversibility experimentally observed. The plot of the spin density provided in Figure 5 indicates that the atoms most involved in the oxidation process are the two irons and the alkylidene carbon.

The IR spectroelectrochemical experiment was repeated in the presence of water (14% v/v) (Figure S12): following the above-described oxidation of **4a** to [**4a**]^+^, a significantly smaller amount of **4a** (about 34%) was recovered in the backward reduction step (Figure S13). Although we were unable to ascertain the formation of **5a** at the end of this experiment (the broad IR water band at 1650 cm^–1^ would cover the absorptions of **5a**), in principle a possible route to **5a** might be determined by the synergistic action of dioxygen, leading to the accessible mono-oxidation of **4a** and water, providing an accelerating effect on the decomposition of [**4a**]^+^.

**Figure 6:**
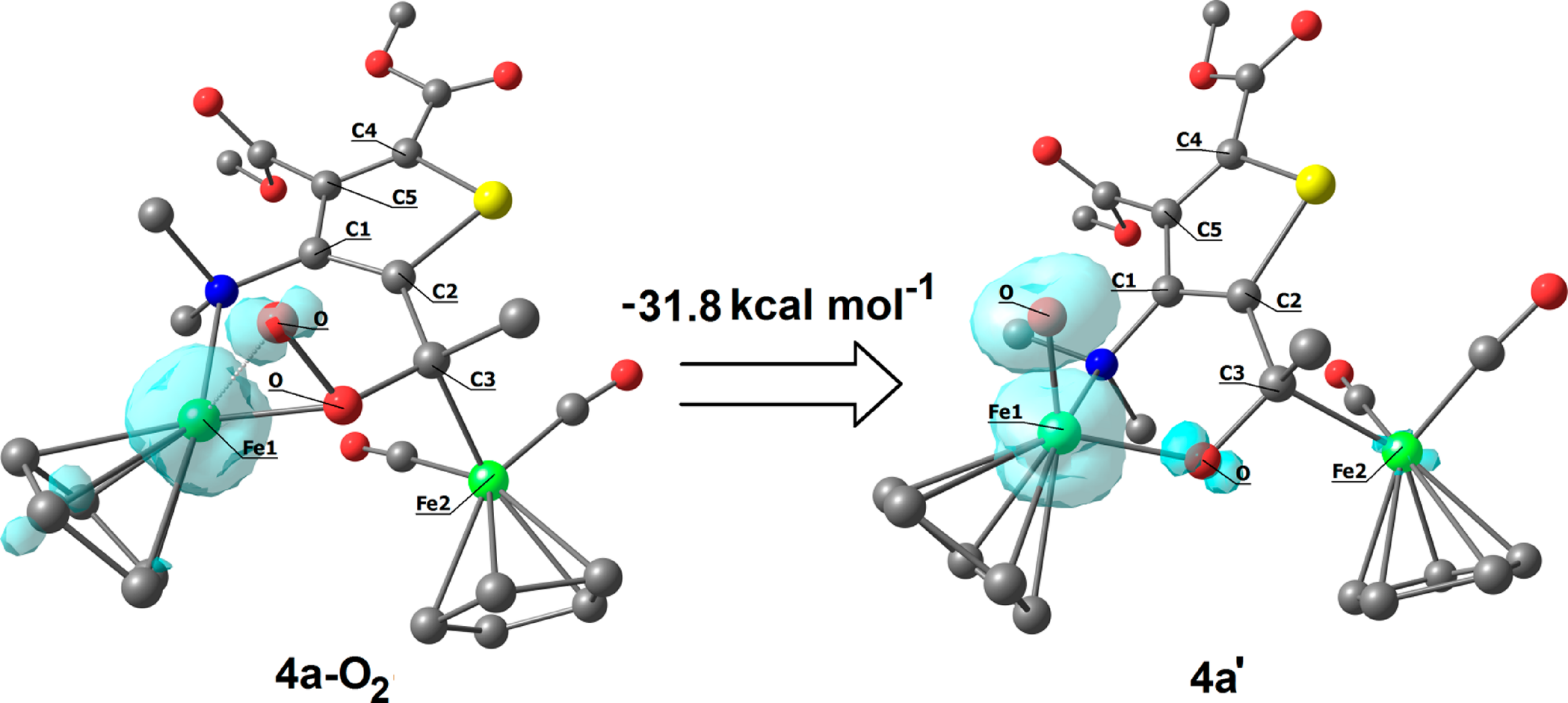
DFT-optimized structures of **4a-O**_**2**_ and **4a**′ (C-PCM/ωB97X/def2-SVP, water as continuous medium) and spin density plots (surface isovalue = 0.01 a.u.). Fe, green; Se, yellow; O, red; N, blue; and C, grey. Hydrogen atoms are omitted for clarity. Selected computed bond lengths for **4a-O**_**2**_ (Å): Fe^1^–O 1.952, 2.137; O–O 1.449; C^3^–O 1.419; C^3^–Fe^2^ 2.098; C^3^–C^2^ 1.517; C^1^–C^2^ 1.388; and C^1^–N 1.461. Selected computed bond lengths for **4a**′ (Å): Fe^1^–O 1.615, 1.866; C^3^–O 1.373; C^3^–Fe^2^ 2.138; C^3^–C^2^ 1.506; C^1^–C^2^ 1.388; and C^1^–N 1.462.

However, analyzing the interaction of **4a** (singlet state) with O_2_ (triplet state) by DFT, we found another plausible pathway for the generation of **5a**. Thus, after several studies, we recognized a possible key intermediate (i.e., **4a-O**_**2**_ (Figure 6)); the Δ*G* on going from **4a** to **4a-O**_**2**_ is −9.9 kcal mol^–1^. Compound **4a-O**_**2**_ contains the [O_2_] fragment interacting with both C^3^ and one iron atom (Fe^1^), with the two Fe^1^–O distances being significantly different (1.952 and 2.137 Å). The O–O bond length (1.449 Å) is close to values of typical organic peroxides.^32a,34^ Moreover, the spin density plot drawn in Figure 6 indicates scarce localization on the [O_2_] fragment of unpaired electrons, which are localized on Fe^1^ instead. In **4a-O**_**2**_, both Fe^1^ and Fe^2^ are formally Fe^II^, but their coordination spheres are somehow different. In fact, Fe^1^ is surrounded by one cyclopentadienide ion, a nitrogen atom, and the peroxido moiety: this relatively weak ligand field may explain the spin density localization on Fe^1^. On the other hand, Fe^2^ is coordinated to one cyclopentadienide and two carbonyl ligands, and its coordination sphere is saturated by the interaction with C^3^. It may be concluded that the interaction of O_2_ with **4a** might lead to formal bielectronic oxidation of the iron centers, with dioxygen converting to peroxide. The interaction of the peroxide unit with C^3^ and Fe^1^ is required to make this reaction thermodynamically viable, since the basic bielectronic oxidation reaction **4a** + O_2_ → [**4a**]^2+^ + [O_2_]^2–^ is associated with a strongly positive Gibbs energy variation, consistent with the electrochemical outcomes (second oxidation potential = +0.47 V). The cleavage of the O–O bond in **4a-O**_**2**_ has been DFT investigated upon slight elongation of the O–O distance. The resulting species, **4a**′, comprises a terminal oxido ligand (Fe^1^–O bond length = 1.615 Å), and its formation is highly favorable on theoretical grounds (Δ*G* = −31.2 kcal mol^–1^). The selenophene ligand remains coordinated to the two irons by the amine group, C^3^ and the newly formed C^3^–O moiety. The following steps are rather hard to rationalize, but it is reasonable that the subsequent interaction of **4a**′ with the solvent, H_2_O, and/or O_2_ will afford the experimentally detected final species **5a**.

In summary, the complexity of the reaction environment and the spectroelectrochemical and DFT results suggest that more mechanistic pathways are viable, finally leading to the ligand dissociation, and both H_2_O and O_2_ are potential sources for the ketonic oxygen in the organic product. Also, the solvent probably plays some role in the decomposition of the diiron frame on the basis of the fact that the reaction is considerably more efficient in ethers than in dichloromethane. Note that oxygen abstraction from 1,2-dimethoxyethane by Lewis acid metal centers was previously documented.^35^ However, the preliminary oxidation of the diiron alkylidene precursor (**4a**–**j**) and the synergic action of O_2_ and H_2_O appear to be necessary.

### Concluding Remarks

The development of organometallic reagents based on earth-abundant and nontoxic metals is highly desirable for specific synthetic purposes, looking to the design of functionalized organic compounds otherwise not accessible. [Fe_2_Cp_2_(CO)_4_] is a commercial chemical that can be employed for the stepwise, non-conventional assembly of various organic and inorganic units, assisted by the cooperative effects provided by the dimetal frame. Herein, we have exploited this approach for the preparation of an unprecedented family of functionalized selenophenes, finally isolated by exploiting the facile release from coordination of a bridging carbene and conversion to a ketone function which is incorporated into the organic product. A multitechnique study (DFT, IR-spectroelectrochemistry, and NMR and Raman spectroscopy) has been carried out to give insight into this exceptional example of bridging alkylidene dissociation, and combined DFT and electrochemical results provide evidence of the synergic action of dioxygen and water in 1,2-dme solution. Our synthesis strategy offers access to a peculiar class of selenophenes for which alternative procedures are missing in the literature and is practical on account of the cheapness and the availability of the iron-based organometallic reagent, the environmentally benign nature of the inorganic residue (iron oxide), and the substantial good/excellent yield character of the reaction steps (organometallic intermediates up to step d can be prepared on gram/multigram scales (Scheme 1)).

## Experimental Section

### Synthesis and Characterization of Compounds

#### General Details

Unless otherwise specified, synthesis and purification procedures (including those reported in the Supporting Information) were conducted under an N_2_ atmosphere using standard Schlenk techniques, and isolated products were stored in air. The synthesis and characterization of [**2a**–**d**]CF_3_SO_3_, **3a**–**c**, and **4a**–**j** are reported in the Supporting Information. Compounds [**1**]CF_3_SO_3_^24^ and **3d**(36) were prepared according to published procedures. Organic reactants (TCI Europe or Merck) and [Fe_2_Cp_2_(CO)_4_] (Strem) were commercial products of the highest purity available. Solvents were purchased from Merck, distilled under N_2_ from appropriate drying agents and stored over molecular sieves. Chromatography separations were carried out on columns of deactivated alumina (Merck, 4% w/w water). Infrared spectra of solid samples were recorded on a Perkin Elmer Spectrum One FT-IR spectrometer equipped with a UATR sampling accessory (4000–400 cm^–1^ range). Infrared spectra of solutions were recorded on a Perkin Elmer Spectrum 100 FT-IR spectrometer with a CaF_2_ liquid transmission cell (2300–1500 cm^–1^ range). IR spectra were processed with Spectragryph software.^37^ The Raman spectrum was recorded with a μ-Raman Invia instrument (Renishaw) equipped with a Leica microscope (50× objective), a diffraction grating with 1800 grooves/mm, and a CCD detector. A HeNe (λ = 633 nm) laser source was used, maintaining the laser power on the sample below 0.2 mW. NMR spectra were recorded at 298 K on a Bruker Avance II DRX400 instrument equipped with a BBFO broadband probe. Chemical shifts (expressed in parts per million) are referenced to the residual solvent peaks^38^ (^1^H, ^13^C) or to an external standard (^77^Se, SeMe_2_). ^1^H and ^13^C NMR spectra were assigned with the assistance of ^1^H–^13^C (*gs*-HSQC and *gs*-HMBC) correlation experiments.^39^ Elemental analyses were performed on a Vario MICRO cube instrument (Elementar). Mass spectrometry measurements in positive ion scan mode were performed on **5a**–**j** (samples dissolved in acetonitrile) with an API 4000 instrument (SCIEX) equipped with an Ionspray/APCI source.

##### Synthesis of **5a**–**j**

##### General Procedure

Compounds **4a**–**j** were dissolved in aerated 1,2-dimethoxyethane (15 mL). Each solution was added to an excess of H_2_O (2–5 mL), and the resulting mixture was stirred at ambient temperature for 24 h (**5a**–**e**, **5i**–**j**) or at 60 °C for 3 h (**5f**–**h**). The volatile materials were removed under vacuum, and the product was extracted with diethyl ether (3 × 15 mL). The organic phase was filtered through an alumina column in air, and a yellow band was collected corresponding to the product. The obtained solution was dried under vacuum, affording a yellow solid. The products were isolated as crystalline materials from the respective concentrated pentane solutions at −30 °C, except for **5e** (oil).

The inorganic residue obtained from **4a** was dried under vacuum and then characterized by Raman spectroscopy. The spectrum, obtained by setting a low laser power (0.15 mW) and a short integration time, was clearly identifiable as lepidocrocite, γ-FeO(OH), an iron oxide-hydroxide mineral featured by a strong band at 250 cm^–1^ (Figure S2). Instrumental parameters are of relevance here, since measurements with laser power >0.2 mW or long acquisition times are known to induce the thermal conversion of lepidocrocite to hematite.^40^

The synthesis of **5a** from **4a** (ca. 0.5 mmol) was also carried out also using THF-*d*^8^ as a solvent (ca. 1 mL). An aliquot of the reaction mixture (0.2 mL) was filtered through a short alumina pad, and the filtered solution was diluted with CDCl_3_. The final solution was analyzed by ^1^H NMR spectroscopy, evidencing the formation of CpH [δ = 6.51 (2H), 6.41 (2H), 2.90 (2H) ppm]^41^ in an admixture with **5a** (CpH/**5a** ratio ≈ 2).

##### SeC^1^{C(O)Me}C^2^(NMe_2_)C^3^(CO_2_Me)C^4^(CO_2_Me) (**5a**) (Chart 1)

**Chart 1:**
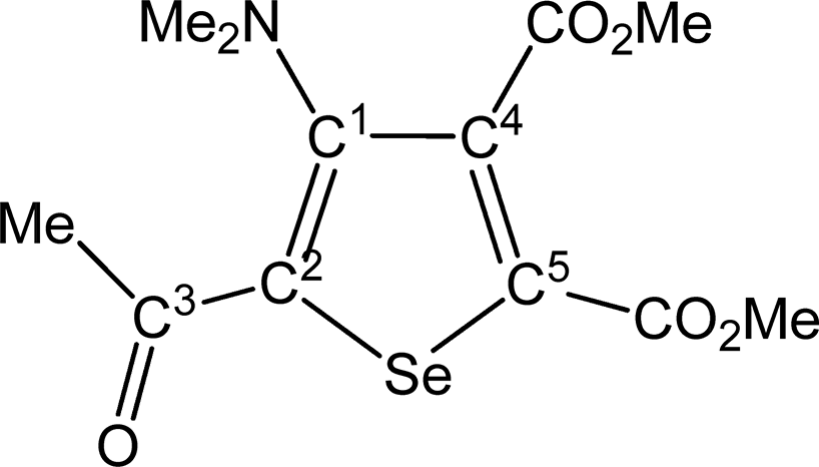
Structure of **5a**

From **4a** (190 mg, 0.309 mmol). Yellow solid, yield 62%. Anal. Calcd for C_12_H_15_NO_5_Se: C, 43.39; H, 4.55; N, 4.22. Found: C, 43.33; H, 4.60; N, 4.25. IR (CH_2_Cl_2_): υ̃/cm^–1^ = 1736 vs (CO_2_Me), 1718 vs (CO_2_Me), 1645 s (C^3^=O), 1542 m (C=C). IR (solid): υ̃/cm^–1^ = 2958 (w), 2837 (w), 2793 (w), 1736 (vs) (CO_2_Me), 1709 (vs) (CO_2_Me), 1635 (m–s) (C^3^=O), 1543 (m) (C=C), 1487 (w), 1451 (w), 1433 (m), 1420 (m), 1366 (m), 1300 (m), 1246 (vs), 1204 (vs), 1174 (m), 1101 (m), 1045 (m), 1021 (m), 989 (w), 957 (w), 928 (w), 831 (w–m), 766 (w), 739 (w), 716 (w). ^1^H NMR (CDCl_3_): δ/ppm = 3.96, 3.87 (s, 6 H, CO_2_Me); 2.87 (s, 6H, NMe_2_); 2.61 (s, 3H, C^3^ Me). ^13^C{^1^H} NMR (CDCl_3_): δ/ppm = 191.9 (C^3^); 167.0, 162.0 (*C*O_2_Me); 153.6 (C^1^); 142.2 (C^2^); 140.3, 138.2 (C^4^ + C^5^); 53.2, 53.1 (CO_2_*Me*); 44.2 (NMe_2_); 28.9 (C^3^*Me*). ^77^Se NMR (CDCl_3_): δ/ppm = 591.7. ESI-MS(+): *m/z* found 334 [M + H]^+^.

##### SeC^1^{C(O)Me}C^2^(NMe_2_)C^3^(CO_2_Et)C^4^(CO_2_Et) (**5b**) (Chart 2)

**Chart 2:**
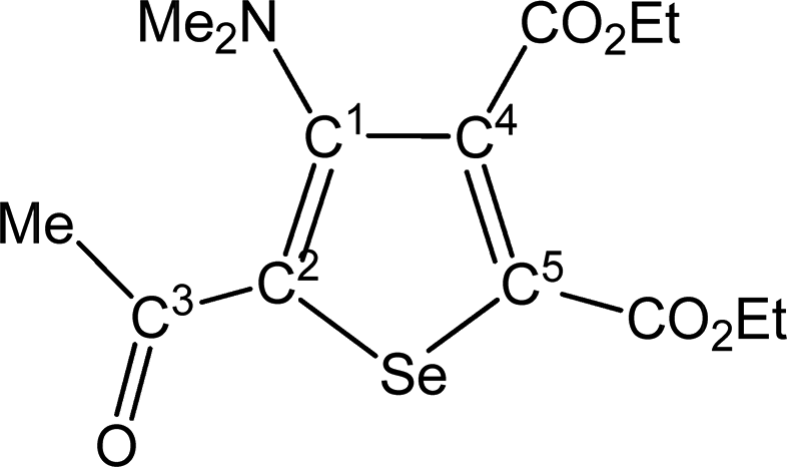
Structure of **5b**

From **4b** (194 mg, 0.302 mmol). Yellow solid, yield 51%. Anal. Calcd for C_14_H_19_NO_5_Se: C, 46.67; H, 5.32; N, 3.89. Found: C, 46.65; H, 5.34; N, 3.94. IR (CH_2_Cl_2_): υ̃/cm^–1^ = 1728 (vs–br) (CO_2_Et), 1643 (m) (C^3^=O), 1541 (w) (C=C). IR (solid): υ̃/cm^–1^ = 2981 (w), 2932 (w), 1716 (vs–br) (CO_2_Et), 1645s (C^3^=O), 1582 (w), 1540 (m) (C=C), 1490 (w), 1446 (w–m), 1416 (w–m), 1363 (m–s), 1299 (m), 1236 (vs), 1186 (vs), 1093s, 1042s, 1020s, 966 (w), 942 (m), 911 (w), 860 (m), 841 (m), 795 (m), 766 (m), 708 (m). ^1^H NMR (CDCl_3_): δ/ppm = 4.41, 4.32 (m, 4H, CH_2_); 2.86 (s, 6H, NMe_2_); 2.60 (s, 3H, C^3^ Me); 1.41, 1.35 (m, 6H, CH_2_C*H*_3_). ^13^C{^1^H} NMR (CDCl_3_): δ/ppm = 191.9 (C^3^); 166.6, 161.6 (*C*O_2_Et); 153.9 (C^1^); 142.1 (C^2^); 140.5, 138.9 (C^4^ + C^5^); 62.3, 62.1 (CH_2_); 43.9 (NMe_2_); 28.9 (C^3^*Me*); 14.1, 13.9 (CH_2_*C*H_3_). ^77^Se NMR (CDCl_3_): δ/ppm = 590.8. ESI-MS(+): *m/z* found 362 [M + H]^+^.

##### SeC^1^{C(O)Me}C^2^(NMe_2_)C^3^(CO_2_^t^Bu)C^4^(CO_2_^t^Bu) (**5c**) (Chart 3)

**Chart 3:**
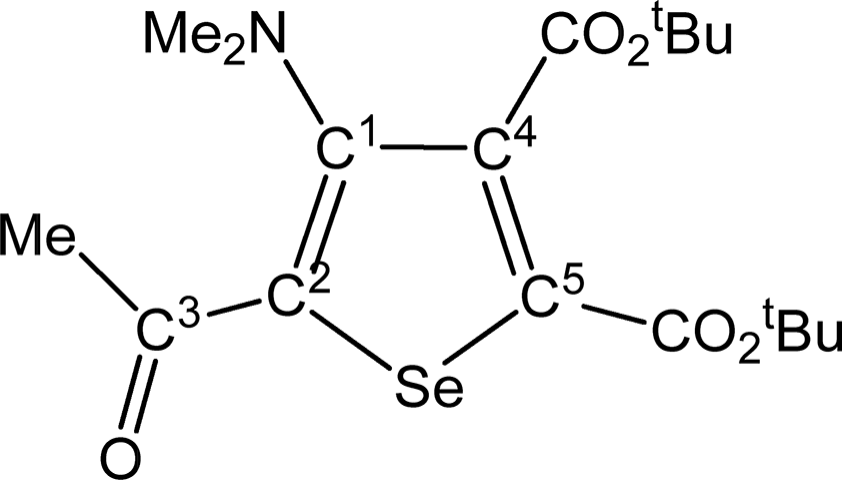
Structure of **5c**

From **4c** (257 mg, 0.368 mmol) and dimethyl acetylenedicarboxylate. Yellow solid, yield 68%. Anal. Calcd for C_18_H_27_NO_5_Se: C, 51.92; H, 6.54; N, 3.36. Found: C, 52.05; H, 6.51; N, 3.39. IR (CH_2_Cl_2_): υ̃/cm^–1^ = 1718 (vs–br) (CO_2_^t^Bu), 1642 (m) (C^3^=O), 1540 (w) (C=C). IR (solid): υ̃/cm^–1^ = 2980 (w), 2932 (w), 1719 (vs) (CO_2_^t^Bu), 1707 (vs) (CO_2_^t^Bu), 1646s (C^3^=O), 1584 (w–m), 1542 (m) (C=C), 1479 (w), 1455 (m), 1422 (w), 1393 (w–m), 1365s, 1309 (m), 1246 (vs), 1149 (vs), 1099s, 1044 (m–s), 1015s, 945 (m), 834 (m–s), 800 (m–s), 761 (m), 729 (w–m), 580 (w–m). ^1^H NMR (CDCl_3_): δ/ppm = 2.87 (s, 6H, NMe_2_); 2.58 (s, 3H, C^3^ Me); 1.60, 1.54 (s, 18 H, CMe_3_). ^13^C{^1^H} NMR (CDCl_3_): δ/ppm = 192.1 (C^3^); 165.2, 160.4 (*C*O_2_^t^Bu); 154.1 (C^1^); 141.7 (C^2^); 141.5, 140.3 (C^4^ + C^5^); 83.8, 83.0 (*C*Me_3_); 43.9 (NMe_2_); 28.7 (C^3^*Me*); 28.0, 27.9 (C*Me*_3_). ^77^Se NMR (CDCl_3_): δ/ppm = 584.4. ESI-MS(+): *m/z* found 418 [M + H]^+^.

##### SeC^1^{C(O)Et}C^2^(NMe_2_)C^3^(CO_2_Me)C^4^(CO_2_Me) (**5d**) (Chart 4)

**Chart 4:**
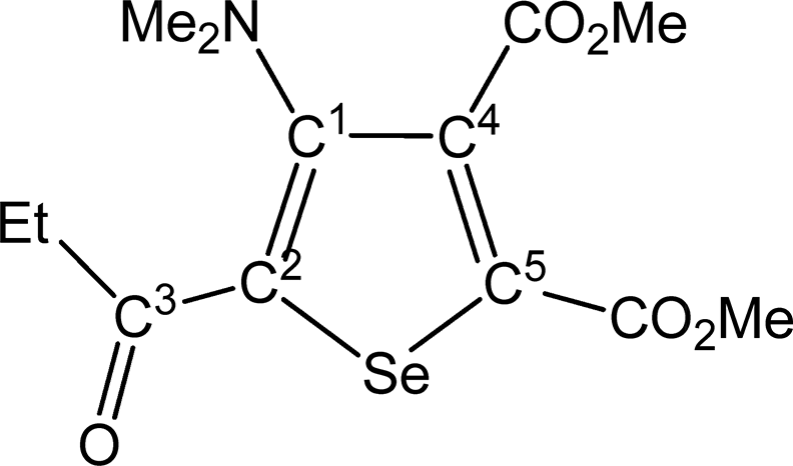
Structure of **5d**

From **4d** (56 mg, 0.089 mmol). Yellow solid, yield 58%. Anal. Calcd for C_13_H_17_NO_5_Se: C, 45.10; H, 4.95; N, 4.05. Found: C, 45.18; H, 5.02; N, 3.98. IR (CH_2_Cl_2_): υ̃/cm^–1^ = 1735 (vs) (CO_2_Me), 1719 (vs) (CO_2_Me), 1644s (C^3^=O), 1542 (m) (C=C). IR (solid state): υ̃/cm^–1^ = 2937 (w), 2796 (w), 1736 (s) (CO_2_Me), 1708 (s) (CO_2_Me), 1637 (m–s) (C^3^=O), 1543 (m–s) (C=C), 1487 (w–m), 1447 (w), 1432 (w–m), 1399 (w), 1369 (m), 1341 (w), 1292 (m), 1252 (m–s), 1229 (m), 1191 (m–s), 1164 (m–s), 1094 (m), 1076 (w–m), 1058 (w), 1033 (m), 991 (m), 958 (m), 855 (m), 839 (m), 804 (w–m), 765 (m), 735 (w–m), 676 (w), 612 (w), 584 (w). ^1^H NMR (CDCl_3_): δ/ppm = 3.96, 3.88 (s, 6H, CO_2_Me); 2.98 (m, 2H, CH_2_); 2.84 (s, 6H, NMe_2_); 1.22 (t, 3 H, ^3^*J*_HH_ = 7.34 Hz, CH_2_C*H*_3_). ^13^C{^1^H} NMR (CDCl_3_): δ/ppm = 195.1 (C^3^); 167.1, 162.1 (*C*O_2_Me); 153.5 (C^1^); 143.3 (C^2^); 141.3, 137.4 (C^4^ + C^5^); 53.1, 53.0 (CO_2_*Me*); 43.8 (NMe_2_); 34.8 (CH_2_); 8.7 (CH_2_*C*H_3_). ^77^Se NMR (CDCl_3_): δ/ppm = 590.9. ESI-MS(+): *m/z* found 348 [M + H]^+^.

##### SeC^1^{C(O)Et}C^2^(NMe_2_)C^3^(CO_2_Et)C^4^(CO_2_Et) (**5e**) (Chart 5)

**Chart 5:**
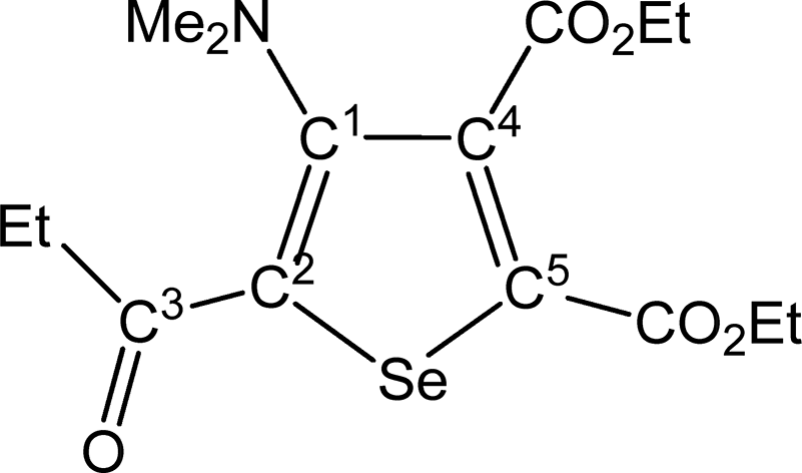
Structure of **5e**

From **4e** (311 mg, 0.474 mmol). Yellow solid, yield 97%. Anal. Calcd for C_15_H_21_NO_5_Se: C, 48.13; H, 5.66; N, 3.74. Found: C, 48.18; H, 5.59; N, 3.77. IR (CH_2_Cl_2_): υ̃/cm^–1^ = 1729 (vs) (CO_2_Et), 1645 (s) (C=O), 1583 (w) (C=C). ^1^H NMR (CDCl_3_): δ/ppm = 4.40, 4.31 (m, 4 H, OCH_2_); 2.95 (m, 2 H, C^3^CH_2_); 2.82 (s, 6 H, NMe_2_); 1.39, 1.34, 1.19 (m, 9 H, CH_2_C*H*_3_). ^13^C{^1^H} NMR (CDCl_3_): δ/ppm = 195.0 (C^3^); 166.6, 161.6 (*C*O_2_Et); 153.6 (C^1^); 141.3 (C^2^); 140.8, 138.0 (C^4^ + C^5^); 62.2, 62.1 (CH_2_); 43.7 (NMe_2_); 34.8 (C^3^*C*H_2_); 14.1, 13.9 (OCH_2_*C*H_3_); 8.6 (C^3^CH_2_*C*H_3_). ^77^Se NMR (CDCl_3_): δ/ppm = 590.1. ESI-MS(+): *m/z* found 376 [M + H]^+^.

##### SeC^1^{C(O)Et}C^2^(NMe_2_)C^3^(CO_2_^t^Bu)C^4^(CO_2_^t^Bu) (**5f**) (Chart 6)

**Chart 6:**
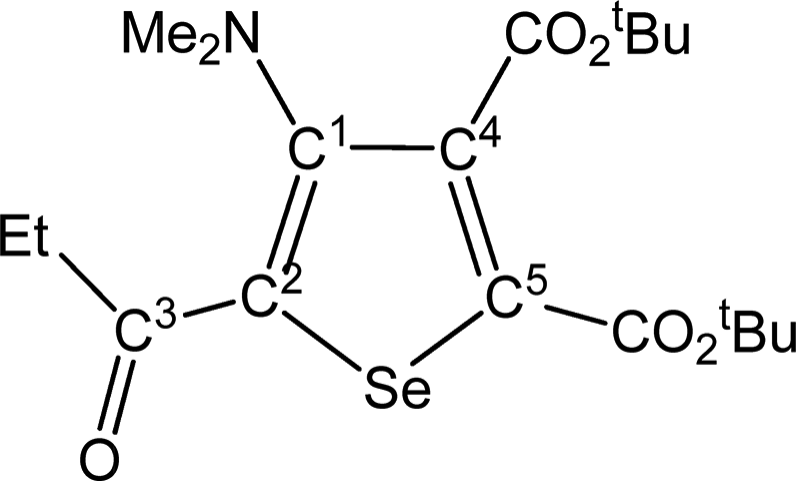
Structure of **5f**

From **4f** (148 mg, 0.208 mmol). Yellow solid, yield 97%. Anal. Calcd for C_19_H_29_NO_5_Se: C, 53.02; H, 6.79; N, 3.25. Found: C, 52.96; H, 6.84; N, 3.30. IR (CH_2_Cl_2_): υ̃/cm^–1^ = 1718 (vs–br) (CO_2_^t^Bu), 1643 (m) (C^3^=O), 1540 (m) (C=C). IR (solid state): υ̃/cm^–1^ = 2978 (w–m), 2932 (w), 2873 (w), 1718 (s) (CO_2_^t^Bu), 1706 (vs) (CO_2_^t^Bu), 1635 (m) (C^3^=O), 1539 (m), 1493 (w), 1476 (w), 1458 (w–m), 1422 (w–m), 1391 (m), 1366 (s), 1332 (w), 1302 (m), 1256 (vs), 1196 (m), 1146 (vs), 1101 (s), 1094 (s), 1069 (s), 1058 (s), 1042 (s), 1021 (s), 944 (m–s), 923 (w), 891 (w), 863 (w–m), 834 (m–s), 798 (s), 760 (m), 734 (m), 708 (w–m), 686 (w–m). ^1^H NMR (CDCl_3_): δ/ppm = 2.94 (q, ^3^*J*_HH_ = 7.34 Hz, 2H, C^3^CH_2_); 2.85 (s, 6H, NMe_2_); 1.61, 1.55 (s, 18 H, CMe_3_); 1.20 (t, ^3^*J*_HH_ = 7.34 Hz, 3H, CH_2_C*H*_3_). ^13^C{^1^H} NMR (CDCl_3_): δ/ppm = 195.2 (C^3^); 165.2, 160.4 (*C*O_2_^t^Bu); 153.9 (C^1^); 142.0, 139.5 (C^4^ + C^5^); 140.7 (C^2^); 82.9, 82.8 (*C*Me_3_); 43.7 (NMe_2_); 34.9 (CH_2_); 28.0, 27.9 (C*Me*_3_); 8.7 (CH_2_*C*H_3_). ^77^Se NMR (CDCl_3_): δ/ppm = 584.8. ESI-MS(+): *m/z* found 432 [M + H]^+^.

##### SeC^1^{C(O)^n^Pr}C^2^(NMe_2_)C^3^(CO_2_Me)C^4^(CO_2_Me) (**5g**) (Chart 7)

**Chart 7:**
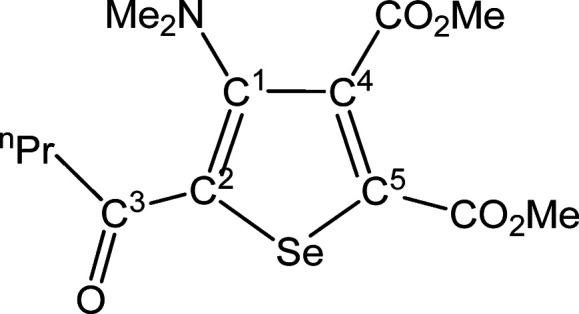
Structure of **5g**

From **4g** (277 mg, 0.431 mmol). Yellow solid, yield 48%. Anal. Calcd for C_14_H_19_NO_5_Se: C, 46.67; H, 5.32; N, 3.89. Found: C, 46.58; H, 5.37; N, 3.79. IR (CH_2_Cl_2_): υ̃/cm^–1^ = 1735 (vs) (CO_2_Me), 1719 (s) (CO_2_Me), 1644 (m) (C^3^=O), 1543 (m) (C=C). IR (solid state): υ̃/cm^–1^ = 2952 (w), 2874 (w), 2798 (w), 1735 (s) (CO_2_Me), 1718 (s) (CO_2_Me), 1644 (m) (C^3^=O), 1540 (m) (C=C), 1488 (w), 1434 (w), 1370 (w), 1300 (w), 1244 (s), 1194 (m), 1161 (m–s), 1102 (w), 1074 (w–m), 1029 (w–m), 988 (w–m), 957 (w–m), 896 (w), 831 (w–m), 796 (w), 767 (m), 754 (w–m). ^1^H NMR (CDCl_3_): δ/ppm = 3.93, 3.85 (s, 6H, CO_2_Me); 2.89 (m, 2H, C^3^CH_2_); 2.82 (s, 6H, NMe_2_); 1.74 (m, 2H, C^3^CH_2_C*H*_2_); 0.98 (m, 3H, CH_2_C*H*_3_). ^13^C{^1^H} NMR (CDCl_3_): δ/ppm = 194.4 (C^3^); 167.0, 162.0 (*C*O_2_Me); 153.5 (C^1^); 140.6, 137.3 (C^4^ + C^5^); 136.7 (C^2^); 52.9 (CO_2_*Me*); 43.7 (NMe_2_); 43.6 (C^3^*C*H_2_); 18.2 (C^3^CH_2_*C*H_2_); 13.8 (CH_2_*C*H_3_). ^77^Se NMR (CDCl_3_): δ/ppm = 590.8. ESI-MS(+): *m/z* found 362 [M + H]^+^.

##### SeC^1^{C(O)^n^Pr}C^2^(NMe_2_)C^3^(CO_2_Et)C^4^(CO_2_Et) (**5h**) (Chart 8)

**Chart 8:**
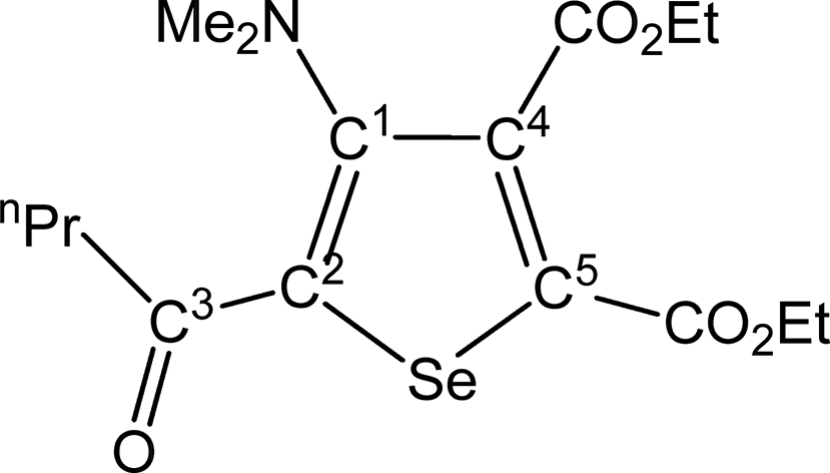
Structure of **5h**

From **4h** (165 mg, 0.246 mmol). Yellow solid, yield 89%. Anal. Calcd for C_16_H_23_NO_5_Se: C, 49.49; H, 5.97; N, 3.61. Found: C, 49.35; H, 6.02; N, 3.68. IR (CH_2_Cl_2_): υ̃/cm^–1^ = 1728 (vs–br) (CO_2_Et), 1642 (w) (C^3^=O), 1540 (w–m) (C=C). IR (solid state): υ̃/cm^–1^ = 2963 (w–m), 2933 (w), 2874 (w), 1729 (s) (CO_2_Et), 1716 (s) (CO_2_Et), 1645 (m) (C^3^=O), 1539 (m) (C=C), 1488 (w–m), 1446 (m), 1365 (m), 1297 (m), 1238 (vs), 1172 (vs), 1159 (vs), 1096 (m), 1071 (m–s), 1019 (s), 953 (m), 861 (w–m), 841 (w–m), 800 (w), 765 (m), 680 (w). ^1^H NMR (CDCl_3_): δ/ppm = 4.42, 4.33 (m, 4 H, OCH_2_); 2.90 (t, 2H, ^3^*J*_HH_ = 7.34 Hz, C^3^CH_2_); 2.85 (s, 6H, NMe_2_); 1.75 (q, 2H, ^3^*J*_HH_ = 7.34 Hz, C^3^CH_2_C*H*_2_); 1.41, 1.36 (q, 6H, OCH_2_C*H*_3_); 1.19 (m, 3H, C^3^CH_2_CH_2_C*H*_3_). ^13^C{^1^H} NMR (CDCl_3_): δ/ppm = ^13^C{^1^H} NMR (CDCl_3_): δ/ppm = 194.5 (C^3^); 166.6, 161.6 (*C*O_2_Et); 153.6 (C^1^); 140.8, 137.9 (C^4^ + C^5^); 136.6 (C^2^); 62.2, 62.1 (OCH_2_); 43.7 (NMe_2_); 43.6 (C^3^*C*H_2_); 18.3 (C^3^CH_2_*C*H_2_); 14.1, 13.9, 13.9 (CH_3_). ^77^Se NMR (CDCl_3_): δ/ppm = 590.1. ESI-MS(+): *m/z* found 390 [M + H]^+^.

##### SeC^1^{C(O)^n^Pr}C^2^(NMe_2_)C^3^(CO_2_^t^Bu)C^4^(CO_2_^t^Bu) (**5i**) (Chart 9)

**Chart 9:**
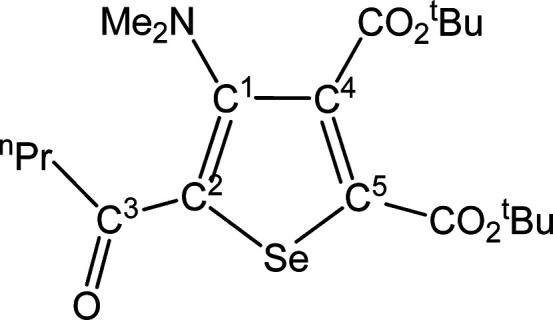
Structure of **5i**

From **4i** (146 mg, 0.201 mmol). Yellow solid, yield 57%. Anal. Calcd for C_20_H_31_NO_5_Se: C, 54.05; H, 7.03; N, 3.16. Found: C, 53.94; H, 6.97; N, 3.18. IR (CH_2_Cl_2_): υ̃/cm^–1^ = 1719 (vs–br) (CO_2_^t^Bu), 1643 (m) (C^3^=O), 1540 (m) (C=C). IR (solid state): υ̃/cm^–1^ = 2966 (w), 2932 (w), 2872 (w), 1715 (s–br) (CO_2_^t^Bu), 1643 (m–s) (C^3^=O), 1541 (m) (C=C), 1485 (w), 1422 (w), 1393 (w), 1368 (m), 1313 (m), 1253 (s), 1154 (m–s), 1108 (m), 1086 (m), 1019 (w), 957 (m), 886 (w), 850 (w), 833 (w–m), 785 (w), 775 (w), 752 (w–m), 722 (w–m), 674 (w), 618 (w). ^1^H NMR (CDCl_3_): δ/ppm = 2.87 (t, 2H, ^3^*J*_HH_ = 7.34 Hz, C^3^CH_2_); 2.85 (s, 6H, NMe_2_); 1.74 (m, 2H, C^3^CH_2_C*H*_2_); 1.61, 1.55 (s, 18H, CMe_3_); 0.98 (m, 3H, CH_2_C*H*_3_). ^13^C{^1^H} NMR (CDCl_3_): δ/ppm = 194.7 (C^3^); 165.3, 160.4 (*C*O_2_^t^Bu); 153.9 (C^1^); 142.0, 139.4 (C^4^ + C^5^); 140.1 (C^2^); 82.9, 82.8 (*C*Me_3_); 43.7 (NMe_2_); 43.7 (C^3^*C*H_2_); 28.0, 27.9 (C*Me*_3_); 18.3 (C^3^CH_2_*C*H_2_); 13.9 (CH_2_*C*H_3_). ^77^Se NMR (CDCl_3_): δ/ppm = 584.5. ESI-MS(+): *m/z* found 446 [M + H]^+^.

##### SeC^1^{C(O)^n^Bu}C^2^(NMe_2_)C^3^(CO_2_Me)C^4^(CO_2_Me) (**5j**) (Chart 10)

**Chart 10:**
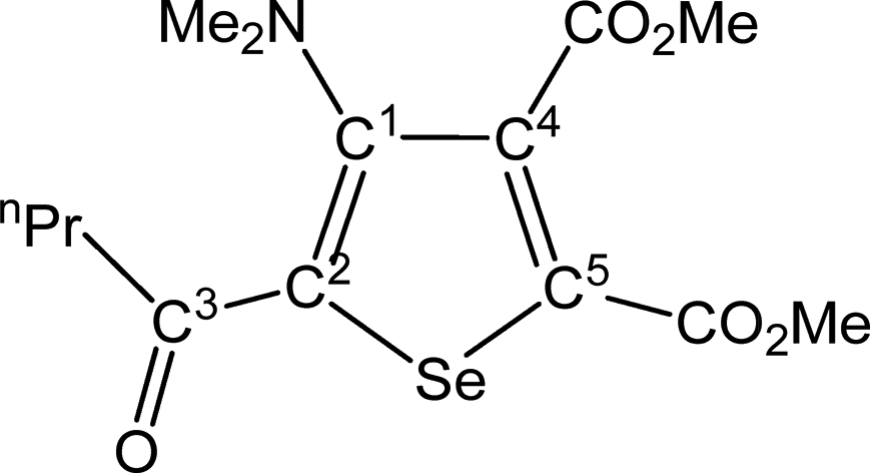
Structure of **5j**

From **4j** (180 mg, 0.274 mmol). Yellow solid, yield 75%. Anal. Calcd for C_15_H_21_NO_5_Se: C, 48.13; H, 5.66; N, 3.74. Found: C, 48.20; H, 5.72; N, 3.72. IR (CH_2_Cl_2_): υ̃/cm^–1^ = 1735 (vs) (CO_2_Me), 1720 (s) (CO_2_Me), 1642 (m) (C^3^=O), 1543 (m) (C=C). IR (solid state): υ̃/cm^–1^ = 2948 (w), 2871 (w), 2796 (w), 1737 (s) (CO_2_Me), 1709 (vs) (CO_2_Me), 1637 (m) (C^3^=O), 1544 (m) (C=C), 1485 (w), 1448 (w), 1434 (m), 1423 (w), 1399 (w), 1373 (w), 1338 (w), 1296 (m), 1251 (vs), 1223 (s), 1189 (m), 1162 (vs), 1111 (m), 1092 (m), 1034 (m), 1021 (w), 993 (m), 961 (w), 926 (w), 871 (w), 837 (m), 768 (w–m), 740 (w), 687 (w–m). ^1^H NMR (CDCl_3_): δ/ppm = 3.96, 3.87 (s, 6H, CO_2_Me); 2.92 (t, ^3^*J*_HH_ = 7.34 Hz, 2H, C^3^CH_2_); 2.84 (s, 6H, NMe_2_); 1.71, 1.40 (m, 4H, C^3^CH_2_C*H*_2_C*H*_2_); 0.95 (t, ^3^*J*_HH_ = 7.34 Hz, 3H, CH_2_C*H*_3_). ^13^C{^1^H} NMR (CDCl_3_): δ/ppm = 194.6 (C^3^); 167.1, 162.1 (*C*O_2_Me); 153.5 (C^1^); 140.7, 137.3 (C^4^ + C^5^); 135.0 (C^2^); 53.1, 52.9 (CO_2_*Me*); 43.7 (NMe_2_); 41.4 (C^3^*C*H_2_); 26.9, 22.5 (C^3^CH_2_*C*H_2_*C*H_2_); 13.9 (CH_2_*C*H_3_). ^77^Se NMR (CDCl_3_): δ/ppm = 591.0. ESI-MS(+): *m/z* found 376 [M + H]^+^.

### X-ray Crystallography

Crystal data and collection details for **4e** and **5a** are reported in Table S1. Data were recorded on a Bruker APEX II diffractometer equipped with a PHOTON100 detector using Mo Kα radiation. Data were corrected for Lorentz polarization and absorption effects (empirical absorption correction SADABS).^42^ Structures were solved by direct methods and refined by full-matrix least squares based on all data using *F*^2^.^43^ Hydrogen atoms were fixed at calculated positions and refined by a riding model. All non-hydrogen atoms were refined with anisotropic displacement parameters.

### Electrochemistry

Cyclic voltammetric measurements were performed with a PalmSens4 instrument interfaced to a computer employing PSTrace5 electrochemical software. 1,2-Dimethoxyethane (Merck) was distilled from calcium hydride under an Ar atmosphere and stored over 3 Å molecular sieves. [NBu_4_]PF_6_ (Fluka, electrochemical grade) was used without further purification. CV measurements were carried out at ambient temperature under Ar using a 0.2 M solution of [NBu_4_]PF_6_ in 1,2-dme as the supporting electrolyte. The working and counter electrodes consisted of a Pt disk and a Pt gauze, respectively, both sealed in a glass tube. A Ag/AgCl, KCl sat. electrode was employed as a reference. The three-electrode home-built cell was predried by heating under vacuum and filled with argon; the Schlenk-type construction of the cell maintained anhydrous and anaerobic conditions. The supporting electrolyte solution, prepared under Ar, was introduced into the cell, and the CV of the solvent was recorded. The analyte was then introduced, and voltammograms were recorded. Under the present experimental conditions, the one-electron reduction of ferrocene occurred at *E*° = +0.54 V vs Ag/AgCl, KCl sat. LSV with the renewal of the diffusion layer that made use of a Metrohm 628-10 rotating disk electrode consisting of a platinum disk surrounded by insulating Teflon. Infrared (IR) spectroelectrochemical measurements were carried out using an optically transparent thin-layer electrochemical (OTTLE) cell equipped with CaF_2_ windows, platinum mini-grid working and auxiliary electrodes, and a silver wire pseudoreference electrode.^44^ During the micro-electrolysis procedures, the electrode potential was controlled by a PalmSens4 instrument interfaced to a computer employing PSTrace5 electrochemical software. Argon-saturated 1,2-dme solutions of **4a**, containing 0.2 M [NBu_4_]PF_6_ as the supporting electrolyte, were used. The *in situ* spectroelectrochemical experiments were performed by collecting IR spectra at fixed time intervals during oxidation or reduction, obtained by continuously increasing or lowering the initial working potential at a scan rate of 1.0 mV/s.

### DFT Calculations

The electronic structures of the compounds were optimized using the range-separated ωB97X DFT functional^45^ in combination with Ahlrichs’ split-valence polarized basis set.^46^ The C-PCM implicit solvation model was added to ωB97X calculations, considering chloroform or water as a continuous medium.^47^ The stationary points were characterized by IR simulations (harmonic approximation), from which zero-point vibrational energies and thermal corrections (*T* = 25 °C) were obtained. The software used was *Gaussian 09*.^48^ Cartesian coordinates of the DFT-optimized structures are collected in a separated.xyz file. Plots of the computed electron densities are available in the Supporting Information.

## Supporting Information Available

Synthesis and characterization of [**2a**–**d**]CF_3_SO_3_, **3a**–**c**, **4a**–**j**; X-ray structure of **4e** and details of X-ray diffraction analyses; Raman spectrum; IR spectroelectrochemical studies; formal electrode potentials; DFT optimized structures; NMR spectra of compounds (PDF)
Cartesian coordinates of the DFT-optimized structure (XYZ)

## Supplementary Material

ic0c02748_si_001.pdf

ic0c02748_si_002.xyz

## Accession Codes

CCDC 2006799–2006800 contain the supplementary crystallographic data for this paper. These data can be obtained free of charge via www.ccdc.cam.ac.uk/data_request/cif, or by emailing data_request@ccdc.cam.ac.uk, or by contacting The Cambridge Crystallographic Data Centre, 12 Union Road, Cambridge CB2 1EZ, UK; fax: +44 1223 336033.
