# Supplementary material for: Tetrasubstituted Selenophenes from the Stepwise Assembly of Molecular Fragments on a Diiron Frame and Final Cleavage of a Bridging Alkylidene
Source: Inorg Chem. 2020 Nov 18;59(23):17497–508. doi: 10.1021/acs.inorgchem.0c02748 (PMC8016200; doi:10.1021/acs.inorgchem.0c02748)
Supplement: Supplementary file 1 — ic0c02748_si_001.pdf [file ic0c02748_si_001.pdf]

Tetrasubstituted Selenophenes from the Stepwise Assembly of Molecular Fragments on a Diiron Frame and Final Cleavage of a Bridging Alkylidene

Giacomo Provinciali, Marco Bortoluzzi, Tiziana Funaioli, Stefano Zacchini, Beatrice Campanella, Guido Pampaloni, Fabio Marchetti

[a] *Dipartimento di Chimica e Chimica Industriale, Università di Pisa, Via G. Moruzzi 13, I-56124 Pisa, Italy*

[b] *Ca' Foscari Università di Venezia, Dipartimento di Scienze Molecolari e Nanosistemi, Via Torino 155, I-30170 Mestre (VE), Italy*

[c] *Dipartimento di Chimica Industriale "Toso Montanari", Università di Bologna, Viale Risorgimento 4, I-40136 Bologna, Italy*

[d] *Istituto di Chimica dei Composti Organometallici, Consiglio Nazionale delle Ricerche, Via G. Moruzzi 1, I-56124 Pisa, Italy*

Supporting Information

<u>**Table of contents**</u> *Page*

**Synthesis and characterization of [2a-d]CF_3SO_3**	S2
**Synthesis and characterization of 3a-c**	S5
**Synthesis and characterization of 4a-j**	S8
**Figure S1**: X-ray structure of **4e**	S16
**Table S1**: Crystal data and measurement details for **4e** and **5a**	S17
**Figure S2**: Raman spectrum	S18
**Figures S3-S9**: DFT optimized structures	S19-S22
**Table S2**: Formal electrode potentials	S23
**Figures S10-S13**: IR spectro-electrochemical studies	S24-S25
**Figures S14-S85**: NMR spectra of compounds	S26-S72
**References**	S73

Synthesis of [2a-d]CF_3SO_3. *General procedure.* Compound [**1**]CF_3SO_3 (0.80 - 2.0 g) was dissolved into acetonitrile (10 mL) and the solution was treated with Me_3NO (1.3 eq.). The resulting mixture was stirred in air allowing gas release for 1 hour, during which time progressive color darkening was noticed. The complete conversion of the starting material into the acetonitrile adduct [Fe_2Cp_2(CO)(μ-CO)(NCMe){μ-$CNMe_2$}]CF_3SO_3 was checked by IR spectroscopy.[1] The volatiles were removed under vacuum, thus the dark-brown residue was dissolved into dichloromethane (ca. 20 mL). The solution was treated with the appropriate alkyne, and the mixture was stirred at ambient temperature for 48 hours. The final mixture was charged on an alumina column in air. Elution with CH_2Cl_2 and CH_2Cl_2/THF mixtures allowed to remove unreacted alkyne and impurities, then a fraction corresponding to the desired product was collected using acetonitrile as eluent. Removal of the solvent under reduced pressure afforded air-stable solids.

[Fe_2Cp_2(CO)(μ-CO){μ-η^1:η^3-C^3(Me)C^2H$C^1$$NMe_2$}]$CF_3SO_3$, [2a]$CF_3SO_3$ (Chart S1).[2]

Chart S1. Structure of [**2a**]$^+$.

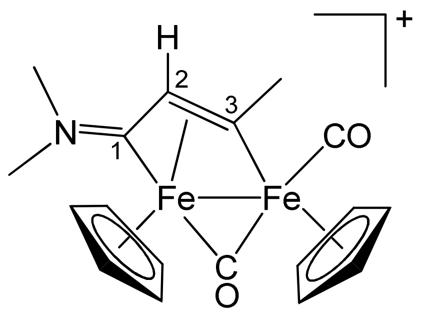

From [**1**]CF_3SO_3 (460 mg, 0.866 mmol) and propyne (3.5 mL of THF solution, ca. 1 mol/L). Brown solid, yield 96%. Anal. calcd. for $C_{19}H_{20}F_3Fe_2NO_5S$: C, 42.02; H, 3.71; N, 2.58; S, 5.90. Found: C, 41.89; H, 3.64; N, 2.62; S, 5.88. IR (CH_2Cl_2): $\tilde{\upsilon}$/cm^{-1} = 1990vs (CO), 1806s (μ-CO), 1684m (C^2C^1N). ^{1}H NMR (dmso-d_6): δ/ppm = 5.48, 5.14 (s, 10 H, Cp); 4.51 (s, 1 H, C^2H); 3.82, 3.77 (s, 6 H, NMe + C^3Me); 3.18 (s, 3 H, NMe). ^{13}C{^{1}H} NMR (dmso-d_6): δ/ppm = 258.4 (*μ*-CO); 225.6 (C^1); 211.4 (CO); 208.0 (C^3); 91.2, 88.0 (Cp); 52.1 (C^2); 51.0, 44.8 (NMe_2); 41.7 (C^3Me).

$[Fe_2Cp_2(CO)(\mu\text{-}CO)\{\mu\text{-}\eta^1:\eta^3\text{-}C^3(Et)C^2HC^1NMe_2\}]CF_3SO_3$, [2b]$CF_3SO_3$ (Chart S2).

Chart S2. Structure of [**2b**]$^+$.

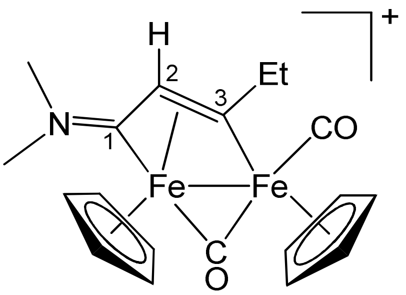

From [**1**]CF_3SO_3 (800 mg, 1.47 mmol) and 1-butyne (large excess bubbled into the CH_2Cl_2 solution). Brown solid, yield 87%. Anal. calcd. for $C_{20}H_{22}F_3Fe_2NO_5S$: C, 43.12; H, 3.98; N, 2.51; S, 5.76. Found: C, 43.02; H, 4.04; N, 2.57; S, 5.80. IR (CH_2Cl_2): $\tilde{\upsilon}$/cm^{-1} = 1991vs (CO), 1806s (μ-CO), 1691m (C^2C^1N). ^1H NMR ($CDCl_3$): δ/ppm = 5.20, 5.02 (s, 10 H, Cp); 4.59 (s, 1 H, C^2H); 4.17, 3.94 (br, 2 H, CH_2); 3.86, 3.28 (s, 6 H, NMe_2); 1.67 (br, 3 H, CH_2CH_3). $^{13}C\{^1H\}$ NMR ($CDCl_3$): δ/ppm = 257.5 (*μ*-CO); 226.0 (C^1); 215.0 (CO); 209.8 (C^3); 89.8, 87.5 (Cp); 51.3 (C^2), 50.7, 44.8 (NMe_2); 48.0 (CH_2); 19.9 (CH_2CH_3).

$[Fe_2Cp_2(CO)(\mu\text{-}CO)\{\mu\text{-}\eta^1:\eta^3\text{-}C^3(^nPr)C^2HC^1NMe_2\}]CF_3SO_3$, [2c]$CF_3SO_3$ (Chart S3).

Chart S3. Structure of [**2c**]$^+$.

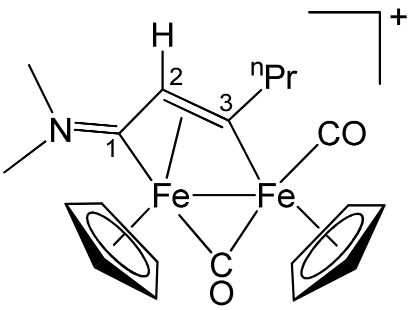

From [**1**]CF_3SO_3 (603 mg, 1.13 mmol) and 1-pentyne (0.33 mL, 3.35 mmol). Brown solid, yield 88%. Anal. calcd. for $C_{21}H_{24}F_3Fe_2NO_5S$: C, 44.16; H, 4.24; N, 2.45; S, 5.61. Found: C, 44.23; H, 4.19; N, 2.49; S, 5.54. IR (CH_2Cl_2): $\tilde{\upsilon}$/cm^{-1} = 1989vs (CO), 1805s (μ-CO), 1683m (C^2C^1N). ^1H NMR (acetone-d_6): δ/ppm = 5.51, 5.22 (s, 10 H, Cp); 4.59 (s, 1 H, C^2H); 4.40, 3.87 (m, 2 H,

C^3CH_2); 3.97, 3.35 (s, 6 H, NMe_2); 2.26, 1.98 (m, 2 H, $C^3CH_2CH_2$); 1.28 (t, 3 H, CH_2CH_3, $^3J_{HH}$ = 6.85 Hz). $^{13}C\{^1H\}$ NMR (acetone-d6): δ/ppm = 257.1 (μ-CO); 226.0 (C^1); 212.9 (C^3); 210.8 (CO); 90.4, 87.6 (Cp); 56.4 (C^3CH_2); 50.9 (C^2), 50.7, 44.3 (NMe_2); 28.3 ($C^3CH_2CH_2$); 13.7 (CH_2CH_3).

[Fe_2Cp_2(CO)(μ-CO){μ-η^1:η^3-C^3(^{n}Bu)C^2HC^1NMe_2}]CF_3SO_3, [2d]CF_3SO_3 (Chart S4).[3]

Chart S4. Structure of [**2d**]$^+$.

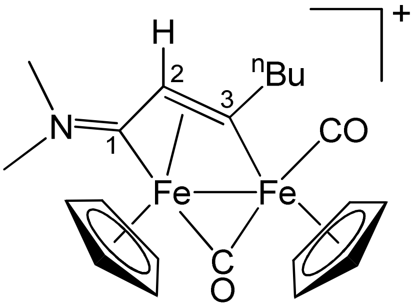

From [**1**]CF_3SO_3 (300 mg, 0.565 mmol) and 1-hexyne (0.15 mL, 1.3 mmol). Brown solid, yield 86%. Anal. calcd. for $C_{22}H_{26}F_3Fe_2NO_5S$: C, 45.15; H, 4.48; N, 2.39; S, 5.48. Found: C, 45.04; H, 4.55; N, 2.34; S, 5.55. IR (CH_2Cl_2): ṽ/cm^{-1} = 1990vs (CO), 1805s (μ-CO), 1682m (C^2C^1N). ^{1}H-NMR ($CDCl_3$): δ/ppm = 5.16, 4.99 (s, 10 H, Cp); 4.52 (s, 1 H, C^2H); 4.14, 3.74 (m, 2 H, C^3CH_2); 3.83, 3.25 (s, 6 H, NMe_2); 2.05, 1.86 (m, 2 H, $C^3CH_2CH_2$); 1.67 (m, 2 H, $C^3CH_2CH_2CH_2$); 1.10 (t, 3 H, $^3J_{HH}$ = 7.32 Hz, CH_2CH_3). $^{13}C\{^1H\}$NMR ($CDCl_3$): δ/ppm = 257.0 (μ-CO); 225.6 (C^1); 212.8 (C^3); 209.6 (CO); 89.6, 87.3 (Cp); 54.7 (C^3CH_2); 51.3 (C^2); 50.9, 44.7 (NMe_2); 37.7 ($C^3CH_2CH_2$); 22.8 ($C^3CH_2CH_2CH_2$); 14.1 (CH_2CH_3).

Synthesis of 3a-c. *General procedure.* Compound [**2a-c**]CF_3SO_3 (0.5-0.8 mmol) was dissolved in THF (10 mL), and the solution was added of grey selenium (ca. 10 eq.) and NaOMe (ca. 2 eq.) in the order given. The mixture was stirred for 30-60 minutes, then IR analysis evidenced the disappearance of the starting diiron complex. The mixture was filtered through a short alumina pad using THF as eluent. Then the solvent was removed under vacuum. In the case of **3a**, the resulting residue was washed with diethyl ether (2 x 30 mL) and dried under vacuum. In the cases of **3b-c**, the residue was dissolved in CH_2Cl_2 and charged on an alumina column: elution with CH_2Cl_2 allowed to remove impurities, then a green fraction was collected using THF as eluent and subsequently the solvent was evaporated under vacuum. Isolated products were stored under N_2.

[Fe_2Cp_2(CO)(μ-CO){μ-η^1:η^3-C^3(Me)C^2(Se)$C^1$$NMe_2$}], 3a (Chart S5).[4]

Chart S5. Structure of **3a**.

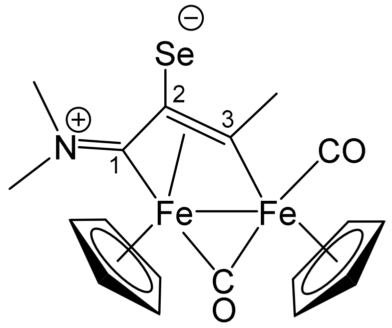

From [**2a**]CF_3SO_3 (258 mg, 0.475 mmol). Green solid, yield 82%. Anal. calcd. for $C_{18}H_{19}Fe_2NO_2Se$: C, 45.80; H, 4.06; N, 2.97. Found: C, 45.89; H, 3.99; N, 3.02. IR (CH_2Cl_2): $\tilde{\upsilon}$/cm^{-1} = 1968vs (CO), 1783s (μ-CO), 1657m (C^2C^1N). IR (THF): $\tilde{\upsilon}$/cm^{-1} = 1959vs (CO), 1781s (μ-CO), 1654m (C^2C^1N). ^1H NMR ($CDCl_3$): δ/ppm = 4.99, 4.56 (s, 10 H, Cp); 4.02 (s, 3 H, C^3Me); 3.76, 3.33 (s, 6 H, NMe_2). $^{13}C\{^1H\}$ NMR ($CDCl_3$): δ/ppm = 265.9 (μ-CO); 226.1 (C^1); 211.0 (CO); 199.7 (C^3); 91.2 (C^2); 90.4, 89.0 (Cp); 45.1, 44.6 (NMe_2); 41.1 (C^3*Me*). ^{77}Se NMR ($CDCl_3$): δ/ppm = 150.6.

[Fe_2Cp_2(CO)(μ-CO){μ-η^1:η^3-C^3(Et)C^2(Se)$C^1$$NMe_2$}], 3b (Chart S6).[4]

Chart S6. Structure of **3b**.

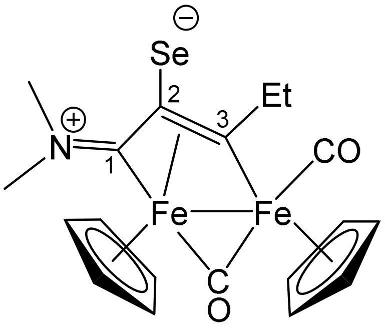

From [**2b**]CF_3SO_3 (179 mg, 0.323 mmol). Dark-green solid, yield 81%. Anal. calcd. for $C_{19}H_{21}Fe_2NO_2Se$: C, 46.95; H, 4.36; N, 2.88. Found: C, 47.03; H, 4.31; N, 2.82. IR (CH_2Cl_2): $\tilde{\upsilon}$/cm^{-1} = 1968vs (CO), 1781s (μ-CO), 1653m (C^2C^1N). IR (THF): $\tilde{\upsilon}$/cm^{-1} = 1959vs (CO), 1781s (μ-CO), 1653m (C^2C^1N). ^1H NMR ($CDCl_3$): δ/ppm = 5.02, 4.60 (s, 10 H, Cp); 4.90, 3.92 (m, 2 H, CH_2); 3.79, 3.33 (s, 6 H, NMe_2); 1.78 (t, 3 H, $^3J_{HH}$ = 7.30 Hz, CH_2CH_3). $^{13}C\{^1H\}$ NMR ($CDCl_3$): δ/ppm = 266.5 (μ-CO); 225.8 (C^1); 211.2 (CO); 208.1 (C^3); 90.6, 88.9 (Cp); 88.2 (C^2); 46.3 (CH_2) 45.1, 44.8 (NMe_2); 16.9 (CH_2CH_3). ^{77}Se NMR ($CDCl_3$): δ/ppm = 130.3.

[Fe_2Cp_2(CO)(μ-CO){μ-η^1:η^3-C^3(^{n}Pr)C^2(Se)C^1NMe_2}], 3c (Chart S7).4

Chart S7. Structure of **3c**.

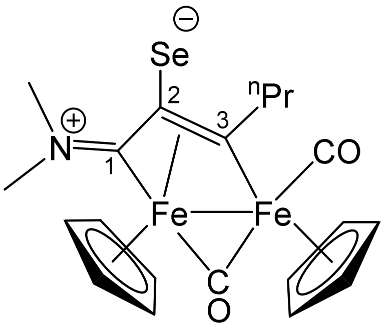

From [**2c**]CF_3SO_3 (178 mg, 0.311 mmol). Dark-green solid, yield 80%. Anal. calcd. for $C_{20}H_{23}Fe_2NO_2Se$: C, 48.04; H, 4.64; N, 2.80. Found: C, 48.11; H, 4.71; N, 2.83. IR (THF): $\tilde{\upsilon}$/cm^{-1} = 1960vs (CO), 1781s (μ-CO), 1653m (C^2C^1N). ^1H NMR ($CDCl_3$): δ/ppm = 5.00, 4.60 (s, 10 H, Cp); 4.81, 3.81 (m, 2 H, C^3CH_2); 3.78, 3.32 (s, 6 H, NMe_2); 2.73, 1.74 (m, 2 H, $C^3CH_2CH_2$); 1.33 (t, 3

H, $^{3}J_{HH}$ = 7.34 Hz, CH_2CH_3). $^{13}C\{^{1}H\}$ NMR ($CDCl_3$): δ/ppm = 266.4 (μ-CO); 225.7 (C^1); 211.2 (CO); 206.3 (C^3); 88.4 (C^2); 90.6, 88.9 (Cp); 55.8 (C^3CH_2); 45.1, 44.7 (NMe_2); 25.6 ($C^3CH_2CH_2$), 15.0 (CH_2CH_3). ^{77}Se NMR ($CDCl_3$): δ/ppm = 134.0.

Synthesis of 4a-j. *General procedure.* A solution of **3a-d** in CH_2Cl_2 (15 mL) was added of the appropriate alkyne reactant (1.2 eq.), and the resulting mixture was stirred overnight at ambient temperature. Then the volatile materials were removed under vacuum, and the residue was charged on an alumina column. Elution with diethyl ether/petroleum ether mixtures allowed to separate impurities. The products were collected using Et_2O/CH_2Cl_2 (1:1 v/v) as eluent and, after removal of the solvent under vacuum, isolated as solid materials which were stored under N_2.

[Fe$_2$Cp$_2$(CO)(μ-CO){μ-κ^1*N*:κ^1*C*:κ^1*C*-C^3(Me)C^2(Se)C^1(NMe$_2$)C^4(CO$_2$Me)C^5(CO$_2$Me)}], 4a (Chart S8).

Chart S8. Structure of **4a**.

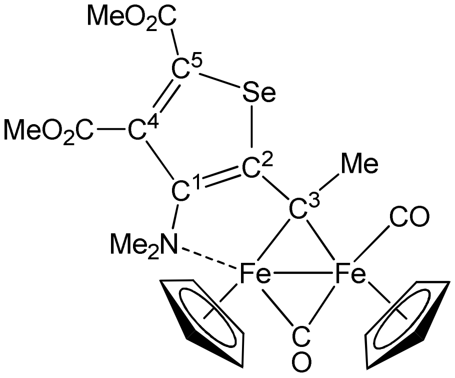

From **3a** (128 mg, 0.271 mmol) and dimethyl acetylenedicarboxylate. Dark-red solid, yield 67%. Anal. calcd. for $C_{24}H_{25}Fe_2NO_6Se$: C, 46.94; H, 4.10; N, 2.28. Found: C, 47.02; H, 4.03; N, 2.35. IR (CH_2Cl_2): $\tilde{\upsilon}$/cm^{-1} = 1932vs (CO), 1749s (μ-CO), 1735s (CO$_2$Me), 1697m (CO$_2$Me), 1605w, 1537w. ^{1}H NMR (CDCl$_3$): δ/ppm = 4.51, 4.38 (s, 10 H, Cp); 3.80, 3.70 (s, 6 H, CO$_2$Me); 3.64 (s, 3 H, C^{3}Me); 2.46, 2.01 (s, 6 H, NMe$_2$). ^{13}C{^{1}H} NMR (CDCl$_3$): δ/ppm = 289.1 (μ-CO); 215.1 (CO); 187.7 (C^3); 168.0, 163.2 (*C*O$_2$Me); 160.2 (C^2); 150.3 (C^1); 137.9, 134.1 (C^4 + C^5); 87.8, 83.8 (Cp); 59.4, 51.9 (NMe$_2$); 52.7 (CO$_2$*Me*); 47.4 (C^3*Me*). ^{77}Se NMR (CDCl$_3$): δ/ppm = 495.9.

[Fe$_2$Cp$_2$(CO)(μ-CO){μ-κ^1*N*:κ^1*C*:κ^1*C*-C^3(Me)C^2(Se)C^1(NMe$_2$)C^4(CO$_2$Et)C^5(CO$_2$Et)}], 4b (Chart S9).

Chart S9. Structure of **4b**.

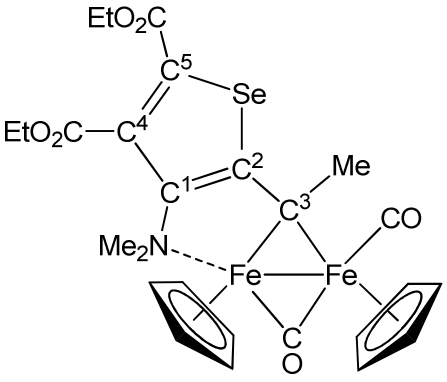

From **3a** (266 mg 0.546 mmol) and diethyl acetylenedicarboxylate (0.90 mL, 0.57 mmol). Dark-red solid, yield 55%. Anal. calcd. for $C_{26}H_{29}Fe_2NO_6Se$: C, 48.63; H, 4.55; N, 2.18. Found: C, 48.73; H, 4.44; N, 2.24. IR (CH_2Cl_2): $\tilde{\upsilon}$/cm^{-1} = 1930vs (CO), 1747s-sh (μ-CO), 1726s (CO_2Et), 1693m (CO_2Et), 1607w, 1536w. ^{1}H NMR ($CDCl_3$): δ/ppm = 4.53, 4.40 (s, 10 H, Cp); 4.33-4.11 (m, 4 H, C*H*$_2$CH$_3$); 3.83 (s, 3 H, C^{3}Me); 2.49, 2.05 (s, 6 H, NMe$_2$); 1.37-1.16 (m, 6 H, CH$_2$C*H*$_3$). ^{13}C{^{1}H} NMR ($CDCl_3$): δ/ppm = 289.6 (μ-CO); 215.1 (CO); 187.4 (C^3); 167.5, 164.8 (*C*O$_2$Et); 162.7 (C^2); 151.8 (C^1); 137.9, 134.3 (C^4 + C^5); 87.8, 83.8 (Cp); 63.0, 61.9 (CH$_2$); 59.6, 52.0 (NMe$_2$); 47.5 (C^3*Me*); 14.2, 13.6 (CH$_2$*C*H$_3$). ^{77}Se NMR ($CDCl_3$): δ/ppm = 494.2.

[Fe$_2$Cp$_2$(CO)(μ-CO){μ-κ^1*N*:κ^1*C*:κ^1*C*-C^3(Me)C^2(Se)C^1(NMe$_2$)C^4(CO$_2$^{t}Bu)C^5(CO$_2$^{t}Bu)}], 4c (Chart S10).

Chart S10. Structure of **4c**.

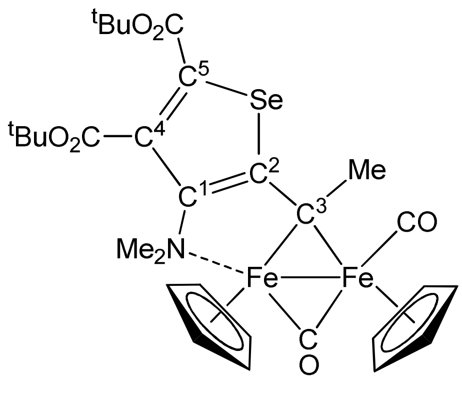

From **3a** (217 mg, 0.460 mmol) and di-*tert*-butyl acetylenedicarboxylate (109 mg, 0.481 mmol). Dark-red solid, yield 80%. Anal. calcd. for $C_{30}H_{37}Fe_2NO_6Se$: C, 51.60; H, 5.34; N, 2.01. Found: C, 51.71; H, 5.33; N, 2.06. ^1H NMR ($CDCl_3$): δ/ppm = 4.52, 4.39 (s, 10 H, Cp); 3.82 (s, 3 H, C^3Me); 2.50, 2.11 (s, 6 H, NMe_2); 1.53 (s, 18 H, CMe_3). $^{13}C\{^1H\}$ NMR ($CDCl_3$): δ/ppm = 290.4 (μ-CO); 215.1 (CO); 185.9 (C^3); 166.1, 165.7 ($\mathit{C}O_2{}^tBu$); 161.5 (C^2); 151.0 (C^1); 138.0, 135.4 (C^4 + C^5); 87.7, 83.8 (Cp); 82.4, 81.0 (*C*Me_3); 59.6, 52.0 (NMe_2); 47.7 ($C^3\mathit{Me}$); 27.9 (CMe_3). ^{77}Se NMR ($CDCl_3$): δ/ppm = 485.5.

[Fe_2Cp_2(CO)(μ-CO){μ-κ^1*N*:κ^1*C*:κ^1*C*-C^3(Et)C^2(Se)C^1(NMe_2)C^4(CO_2Me)C^5(CO_2Me)}], 4d (Chart S11).

Chart S11. Structure of **4d**.

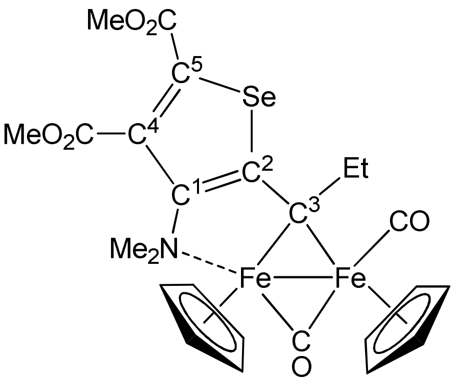

From **3b** (214 mg, 0.441 mmol) and dimethyl acetylenedicarboxylate (63 mg, 0.448 mmol). Dark-red solid, yield 34%. Anal. calcd. for $C_{25}H_{27}Fe_2NO_6Se$: C, 47.80; H, 4.33; N, 2.23. Found: C, 47.72; H, 4.42; N, 2.25. IR (CH_2Cl_2): $\tilde{\upsilon}$/cm^{-1} = 1934vs (CO), 1747s (μ-CO), 1745s (CO_2Me), 1697m (CO_2Me). ^1H NMR ($CDCl_3$): δ/ppm = 4.51, 4.44 (s, 10 H, Cp); 4.07, 3.79 (m, 2 H, CH_2); 3.71, 3.67 (s, 6 H, CO_2Me); 2.55, 2.14 (s, 6 H, NMe_2); 1.22 (m, 3 H, $CH_2C\mathit{H}_3$). $^{13}C\{^1H\}$ NMR ($CDCl_3$): δ/ppm = 287.2 (μ-CO); 214.7 (CO); 186.0 (C^3); 172.8 (C^2); 167.7, 163.1 ($\mathit{C}O_2Me$); 151.8 (C^1); 138.2, 133.9 (C^4 + C^5); 87.7, 83.9 (Cp); 58.8 (NMe); 51.9, 51.8 ($CO_2\mathit{Me}$ + NMe); 43.5 (CH_2); 17.8 ($CH_2\mathit{C}H_3$). ^{77}Se NMR ($CDCl_3$): δ/ppm = 500.6. Crystals suitable for X-ray analysis were obtained from a diethyl ether solution layered with hexane.

[Fe_2Cp_2(CO)(μ-CO){μ-κ^1*N*:κ^1*C*:κ^1*C*-C^3(Et)C^2(Se)C^1(NMe_2)C^4(CO_2Et)C^5(CO_2Et)}], 4e (Chart S12).

Chart S12. Structure of **4e**.

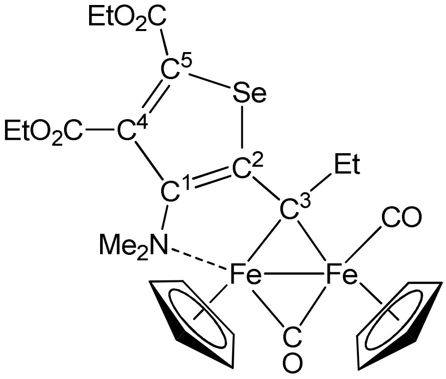

From **3b** (254 mg, 0.524 mmol) and diethyl acetylenedicarboxylate (0.085 mL, 0.531 mmol). Dark-red solid, yield 91%. Anal. calcd. for $C_{27}H_{31}Fe_2NO_6Se$: C, 49.42; H, 4.76; N, 2.13. Found: C, 49.28; H, 4.74; N, 2.20. IR (CH_2Cl_2): ῦ/cm^{-1} = 1935vs (CO), 1746s-sh (μ-CO), 1725s (CO_2Et), 1694m (CO_2Et), 1538w. ^{1}H NMR ($CDCl_3$): δ/ppm = 4.51, 4.44 (s, 10 H, Cp); 4.33, 4.16 (m, 4 H, CH_2); 4.11, 3.80 (m, 2 H, CH_2); 2.55, 2.16 (s, 6 H, NMe_2); 1.36, 1.27, 1.18 (m, 9 H, CH_2CH_3). ^{13}C{^{1}H} NMR ($CDCl_3$): δ/ppm = 288.9 (μ-CO); 214.1 (CO); 185.5 (C^3); 173.2 (C^2); 167.5, 162.8 (*C*O_2Et); 151.8 (C^1); 138.2, 134.0 (C^4 + C^5); 87.6, 83.9 (Cp); 63.0, 61.9, 51.9 (CH_2); 59.0, 52.0 (NMe_2); 14.2, 13.9, 13.6 (CH_2CH_3). ^{77}Se NMR ($CDCl_3$): δ/ppm = 500.2.

[Fe_2Cp_2(CO)(μ-CO){μ-κ^1*N*:κ^1*C*:κ^1*C*-C^3(Et)C^2(Se)C^1(NMe_2)C^4(CO_2^{t}Bu)C^5(CO_2^{t}Bu)}], 4f (Chart S13).

Chart S13. Structure of **4f**.

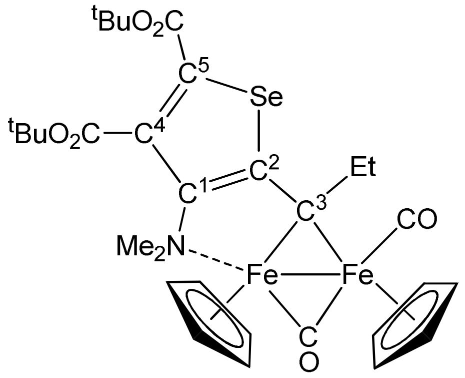

From **3b** (154 mg, 0.317 mmol) and di-*tert*-butyl acetylenedicarboxylate (73 mg, 0.322 mmol). Dark-red solid, yield 65%. Anal. calcd. for $C_{31}H_{39}Fe_2NO_6Se$: C, 52.27; H, 5.52; N, 1.97. Found: C, 52.16; H, 5.60; N, 1.99. ^{1}H NMR ($CDCl_3$): δ/ppm = 4.50, 4.42 (s, 10 H, Cp); 4.06, 3.81 (m, 2 H, CH_2); 2.57, 2.22 (s, 6 H, NMe_2); 2.18 (m, 3 H, CH_2CH_3); 1.48, 1.37 (s, 18 H, CMe_3). $^{13}C\{^1H\}$ NMR ($CDCl_3$): δ/ppm = 289.9 (μ-CO); 213.9 (CO); 183.9 (C^3); 174.4 (C^2); 166.2, 161.6 ($CO_2{}^tBu$); 150.0 (C^1); 138.2, 135.1 (C^4 + C^5); 87.5, 83.8 (Cp); 82.4, 81.0 (CMe_3); 59.0, 52.0 (NMe_2); 52.0 (CH_2); 28.1, 27.6 (CMe_3); 17.9 (CH_2CH_3). ^{77}Se NMR ($CDCl_3$): δ/ppm = 491.4.

[Fe_2Cp_2(CO)(μ-CO){μ-κ^1*N*:κ^1*C*:κ^1*C*-C^3(^{n}Pr)C^2(Se)C^1(NMe_2)C^4(CO_2Me)C^5(CO_2Me)}], 4g (Chart S14).

Chart S14. Structure of **4g**.

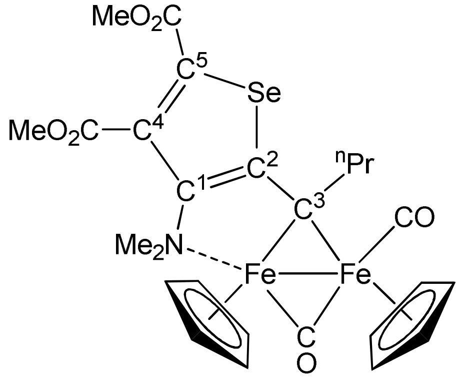

From **3c** (250 mg, 0.500 mmol) and dimethyl acetylenedicarboxylate (73mg, 0.513 mmol). Dark-red solid, yield 86%. Anal. calcd. for $C_{26}H_{29}Fe_2NO_6Se$: C, 48.63; H, 4.55; N, 2.18. Found: C, 48.52; H, 4.60; N, 2.22. IR (CH_2Cl_2): ῡ/cm^{-1} = 1934vs (CO), 1741s (μ-CO), 1750s-sh (CO_2Me),

1695m (CO_2Me). ^{1}H NMR ($CDCl_3$): δ/ppm = 4.51, 4.42 (s, 10 H, Cp); 4.01, 3.78 (m, 2 H, C^3CH_2); 3.71, 3.66 (s, 6 H, CO_2Me); 2.74 (m, 2 H, $C^3CH_2CH_2$); 2.53, 2.13 (s, 6 H, NMe_2); 1.24 (t, 3 H, CH_2CH_3). $^{13}C\{^1H\}$ NMR (CD_2Cl_2): δ/ppm = 288.3 (μ-CO); 214.1 (CO); 186.3 (C^3); 171.0 (C^2); 168.0, 163.3 (CO_2Me); 137.8, 133.9 (C^4 + C^5); 87.7, 83.9 (Cp); 62.5 (C^3CH_2); 59.0, 52.1, 52.0 (CO_2Me + NMe_2); 26.8 ($C^3CH_2CH_2$); 15.8 (CH_2CH_3). C^1 not observed. ^{77}Se NMR (CD_2Cl_2): δ/ppm = 500.9.

[$Fe_2Cp_2(CO)(\mu\text{-}CO)\{\mu\text{-}\kappa^1N:\kappa^1C:\kappa^1C\text{-}C^3(^nPr)C^2(Se)C^1(NMe_2)C^4(CO_2Et)C^5(CO_2Et)\}$], 4h (Chart S15).

Chart S15. Structure of **4h**.

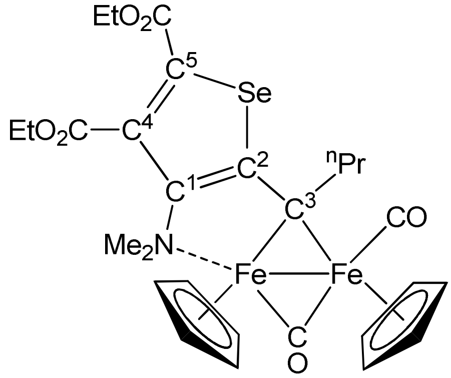

From **3c** (215 mg, 0.430 mmol) and diethyl acetylenedicarboxylate (0.075 mL, 0.468 mmol). Dark-red solid, yield 52%. Anal. calcd. for $C_{28}H_{33}Fe_2NO_6Se$: C, 50.18; H, 4.96; N, 2.09. Found: C, 50.24; H, 5.02; N, 2.12. IR (CH_2Cl_2): $\tilde{\upsilon}$/cm^{-1} = 1935vs (CO), 1747s-sh (μ-CO), 1724s (CO_2Et), 1692m (CO_2Et), 1539w. ^{1}H NMR ($CDCl_3$): δ/ppm = 4.50, 4.40 (s, 10 H, Cp); 4.30-4.09 (m, 4 H, CH_2CH_3); 4.00, 3.67 (m, 2 H, C^3CH_2); 2.73 (m, 2 H, $C^3CH_2CH_2$); 2.51, 2.14 (s, 6 H, NMe_2); 1.40, 1.25, 1.16 (m, 9 H, CH_3). $^{13}C\{^1H\}$ NMR ($CDCl_3$): δ/ppm = 288.9 (μ-CO); 214.0 (CO); 185.9 (C^3); 171.3 (C^2); 167.6, 162.8 (CO_2Et); 137.8, 134.0 (C^4 + C^5); 87.6, 83.9 (Cp); 62.5, 61.9, 61.0 (CH_2CH_3 + C^3CH_2); 59.0, 52.0 (NMe_2); 26.7 ($C^3CH_2CH_2$); 15.8, 14.2, 13.6 (CH_3). ^{77}Se NMR (CD_2Cl_2): δ/ppm = 499.2.

[Fe_2Cp_2(CO)(μ-CO){μ-κ^1*N*:κ^1*C*:κ^1*C*-C^3(Pr)C^2(Se)C^1(NMe_2)C^4($CO_2{}^tBu$)C^5($CO_2{}^tBu$)}], 4i (Chart S16).

Chart S16. Structure of **4i**.

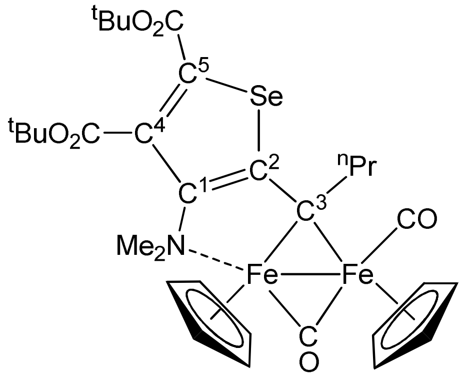

From **3c** (198 mg, 0.346 mmol) and di-*tert*-butyl acetylenedicarboxylate (73 mg, 0.322 mmol). Dark-red solid, yield 55%. Anal. calcd. for $C_{32}H_{41}Fe_2NO_6Se$: C, 52.92; H, 5.69; N, 1.93. Found: C, 53.05; H, 5.61; N, 1.98. IR (CH_2Cl_2): $\tilde{\upsilon}$/cm^{-1} = 1935vs (CO), 1744s-sh (μ-CO), 1716s ($CO_2{}^tBu$), 1693s ($CO_2{}^tBu$). ^{1}H NMR ($CDCl_3$): δ/ppm = 4.50, 4.40 (s, 10 H, Cp); 4.00, 3.69 (m, 2 H, C^{3}CH_2); 2.75 (m, 2 H, C^{3}CH$_2$CH_2); 2.55, 2.22 (s, 6 H, NMe$_2$); 1.48, 1.36 (s, 18 H, CMe$_3$); 1.23 (m, 3 H, C^{3}CH$_2$CH$_2$CH_3). ^{13}C{^{1}H} NMR ($CDCl_3$): δ/ppm = 289.9 (μ-CO); 213.8 (CO); 184.4 (C^3); 172.5 (C^2); 166.2, 161.6 (*C*O$_2{}^t$Bu); 137.9, 135.1 (C^4 + C^5); 87.6, 83.9 (Cp); 82.4, 81.0 (*C*Me$_3$); 62.6 (C^3*C*H$_2$); 58.9, 52.0 (NMe$_2$); 28.1, 27.6 (C*Me*$_3$); 26.7 (C^{3}CH$_2$*C*H$_2$); 15.7 (CH$_2$*C*H$_3$). ^{77}Se NMR (CD_2Cl_2): δ/ppm = 490.5.

[Fe_2Cp_2(CO)(μ-CO){μ-κ^1*N*:κ^1*C*:κ^1*C*-C^3(^{n}Bu)C^2(Se)C^1(NMe_2)C^4(CO_2Me)C^5(CO_2Me)}], 4j (Chart S17).

Chart S17. Structure of **4j**.

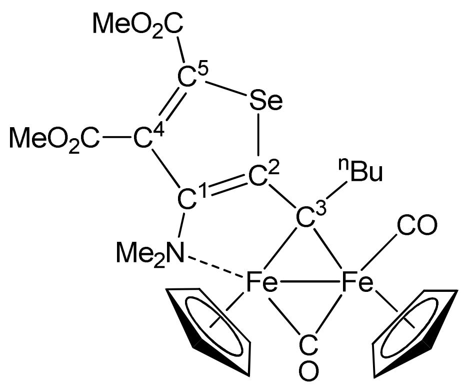

From **3d** (300 mg, 0.584 mmol) and dimethyl acetylenedicarboxylate (0.11 mL, 0.70 mmol). Brown solid, yield 65%. Anal. calcd. for $C_{27}H_{31}Fe_2NO_6Se$: C, 49.42; H, 4.76; N, 2.13. Found: C, 49.48; H, 4.69; N, 2.11. IR (CH_2Cl_2): $\tilde{\upsilon}$/cm^{-1} = 1934s (CO), 1738sh (μ-CO), 1732vs (CO_2Me), 1699s (CO_2Me), 1540w. Satisfying NMR spectra could not be recorded, presumably due to paramagnetic impurities which could not be removed.

Figure S1. Molecular structure of **4e**, with key atoms labelled. Thermal ellipsoids are at the 50% probability level. H-atoms have been omitted for clarity. Selected bond distances (Å) and angles (°): Fe(1)-Fe(2) 2.5384(5), Fe(1)-C(11) 1.747(2), Fe(1)-C(12) 1.958(2), Fe(1)-C(3) 2.047(2), Fe(2)-C(12) 1.858(2), Fe(2)-C(3) 1.976(2), Fe(2)-N(1) 2.181(2), O(11)-C(11) 1.158(3), O(12)-C(12) 1.188(3), C(3)-C(2) 1.460(3), C(2)-C(1) 1.370(3), C(1)-C(4) 1.429(3), C(4)-C(5) 1.372(3), C(5)-Se(1) 1.866(2), Se(1)-C(2) 1.883(2), C(1)-N(1) 1.464(3), C(5)-C(24) 1.463(3), C(4)-C(22) 1.498(3), Fe(1)-C(11)-O(11) 174.2(2), Fe(1)-C(12)-Fe(2) 83.35(10), Fe(1)-C(3)-Fe(2) 78.22(8), Fe(1)-C(3)-C(2) 109.27(16), Fe(2)-C(3)-C(2) 108.36(15), C(3)-C(2)-Se(1) 125.34(17), C(3)-C(2)-C(1) 124.1(2), Se(1)-C(2)-C(1) 110.44(17), C(2)-C(1)-C(4) 116.1(2), C(1)-C(4)-C(5) 113.8(2), C(4)-C(5)-Se(1) 112.26(17), C(5)-Se(1)-C(2) 87.25(10), C(1)-N(1)-Fe(2) 106.24(14).

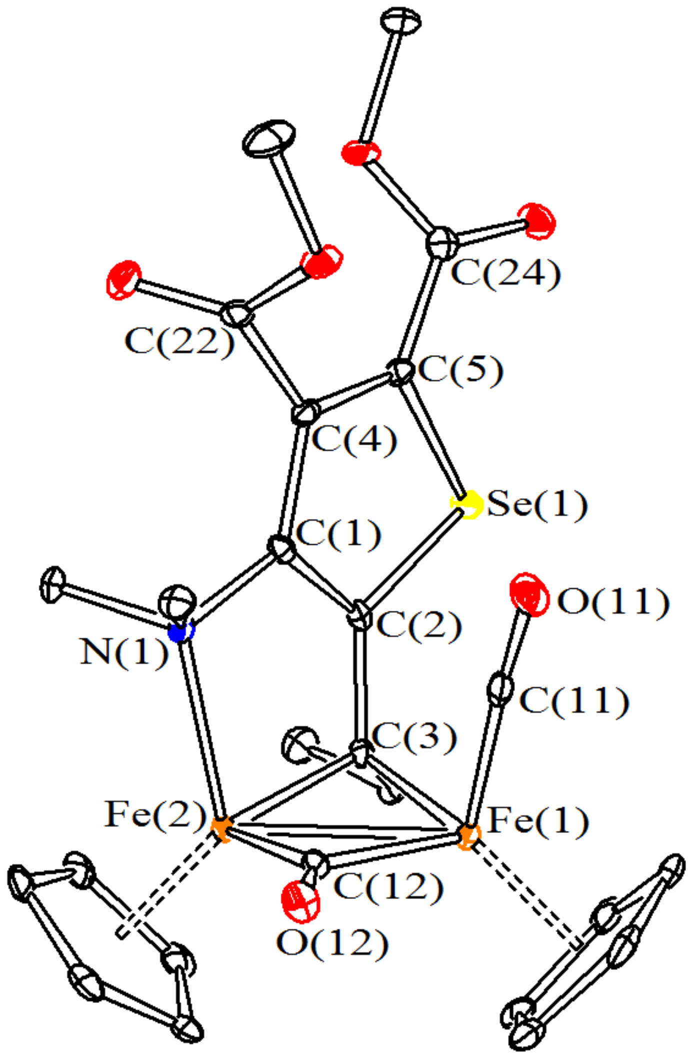

Table S1. Crystal data and measurement details for **4e** and **5a**.

	**4e**	**5a**
Formula	$C_{25}H_{27}Fe_2NO_6Se$	$C_{12}H_{15}NO_5Se$
FW	628.13	332.21
T, K	100(2)	100(2)
λ, Å	0.71073	0.71073
Crystal system	Monoclinic	Monoclinic
Space group	$P2_1/n$	$P2_1/c$
a, Å	8.2872(5)	17.7252(17)
b, Å	14.9958(8)	11.4810(11)
c, Å	19.0769(11)	6.8873(7)
β,°	95.9900(10)	92.523(2)
Cell Volume, $Å^3$	2357.8(2)	1400.2(2)
Z	4	4
D_c, g·cm^{-3}	1.770	1.576
μ, mm^{-1}	2.818	2.697
F(000)	1272	672
Crystal size, mm	0.21×0.18×0.13	0.21×0.15×0.13
θ limits,°	1.731-28.000	2.114-28.000
Reflections collected	35062	20468
Independent reflections	5676 [R_{int} = 0.0408]	3378 [R_{int} = 0.0328]
Data/restraints/parameters	5676 / 0 / 321	3378 / 0 / 177
Goodness of fit on F^2	1.162	1.084
R_1 ($I > 2\sigma(I)$)	0.0359	0.0240
wR_2 (all data)	0.0672	0.05344
Largest diff. peak and hole, e $Å^{-3}$	0.582 / –0.493	0.400 / –0.556

Figure S2. Raman spectrum of the inorganic residue resulting from the reaction of **4a** with H_2O/O_2 in 1,2-dimethoxyethane (633 nm, 0.15 mW, 10 s integration time, 10 accumulations).

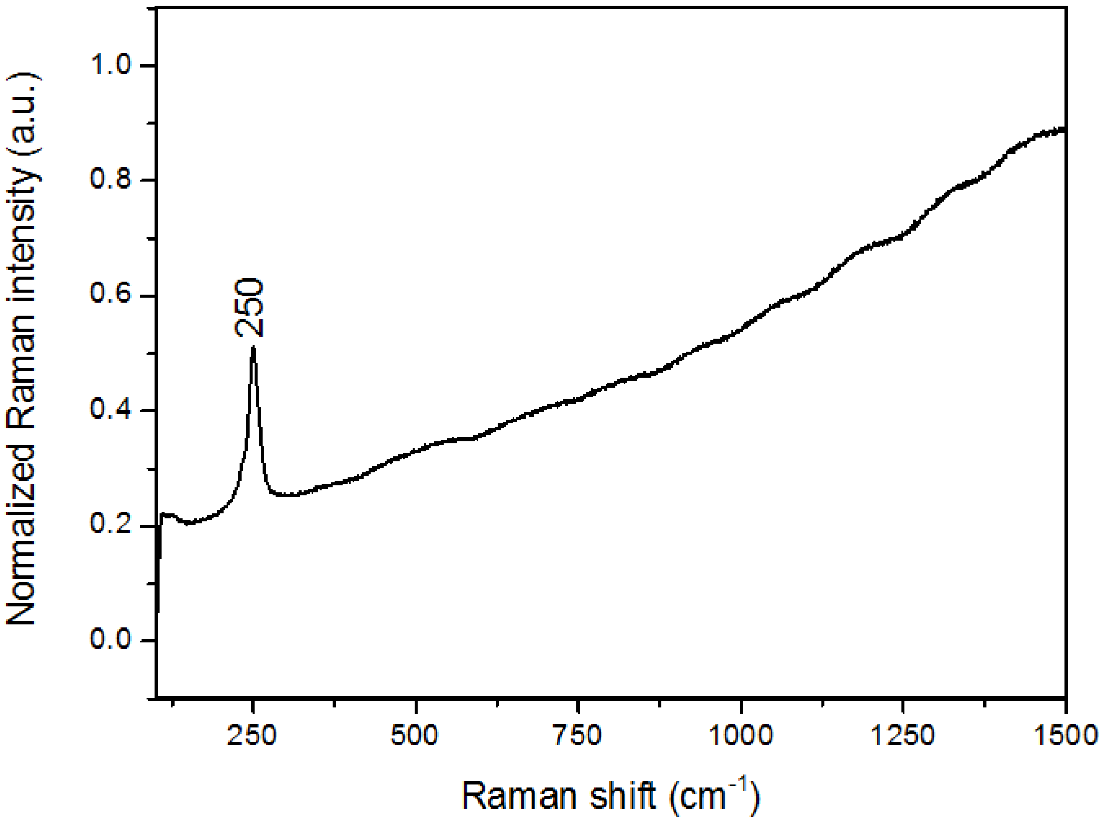

Figure S3. Left: DFT-optimized structure of **3a** (C-PCM/ωB97X/def2-SVP, chloroform as continuous medium). Fe, green; Se, yellow; O, red; N, blue; C, grey. Hydrogen atoms are omitted for clarity. Selected computed bond lengths (Å): Fe1-C1 1.875, C1-N 1.292, C1-C2 1.427, C2-Se 1.900, C2-Fe1 2.109, C2-C3 1.423, C3-Fe1 2.022, C3-Fe2 1.972. Right: electron density surface, isovalue = 0.05 a.u.

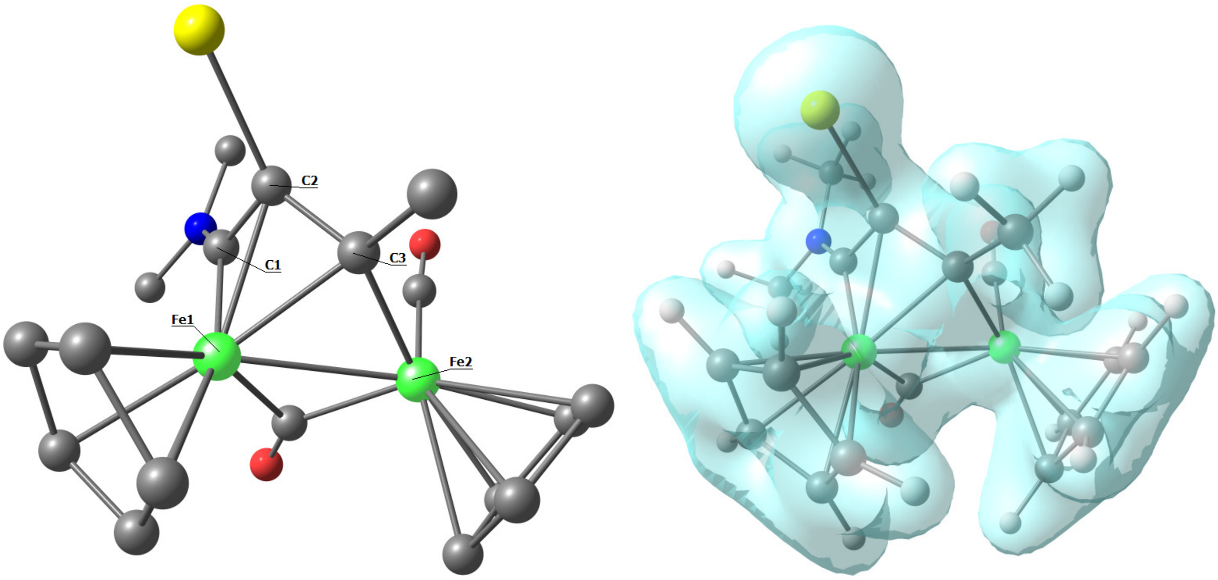

Figure S4. Left: DFT-optimized structure of **INT1** (C-PCM/ωB97X/def2-SVP, chloroform as continuous medium). Fe, green; Se, yellow; O, red; N, blue; C, grey. Hydrogen atoms are omitted for clarity. Selected computed bond lengths (Å): Fe1-C1 1.874, C1-N 1.286, C1-C2 1.428, C2-Se 1.919, Se-C5 1.956, C4-C5 1.331, C2-Fe1 2.073, C2-C3 1.420, C3-Fe1 2.017, C3-Fe2 1.965. Right: electron density surface, isovalue = 0.05 a.u.

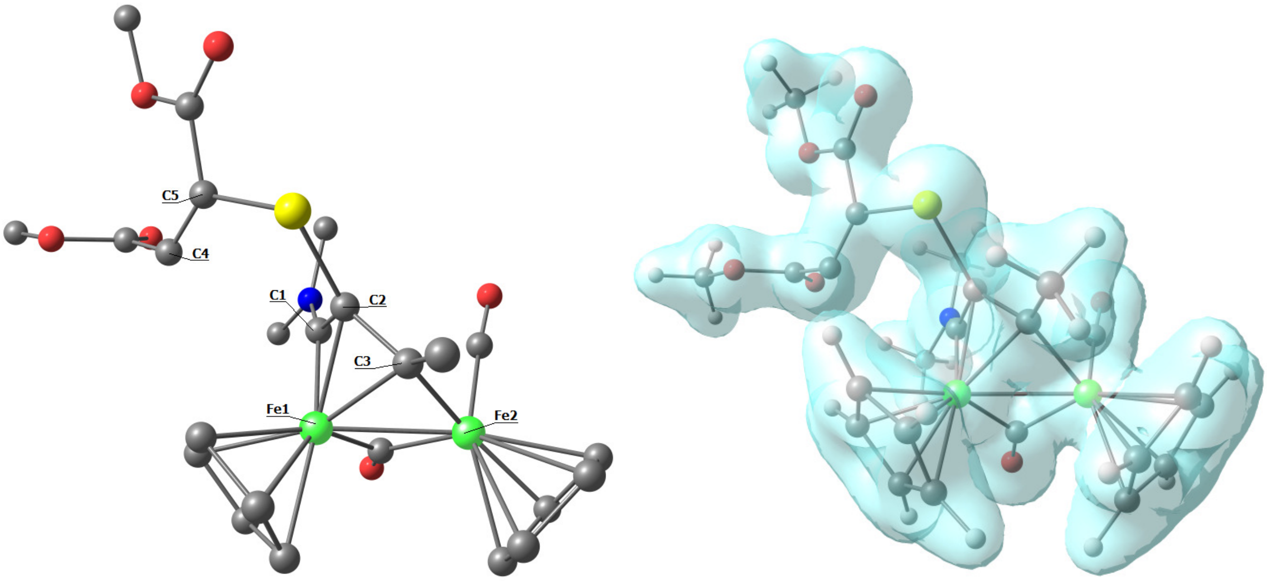

Figure S5. Left: DFT-optimized structure of **TS1** (C-PCM/ωB97X/def2-SVP, chloroform as continuous medium). Fe, green; Se, yellow; O, red; N, blue; C, grey. Hydrogen atoms are omitted for clarity. Selected computed bond lengths (Å): Fe1-C1 1.874, C1-N 1.291, C1-C2 1.427, C2-Se 1.911, Se-C5 2.593, C4-C5 1.237, C2-Fe1 2.098, C2-C3 1.424, C3-Fe1 2.015, C3-Fe2 1.971. Right: electron density surface, isovalue = 0.05 a.u.

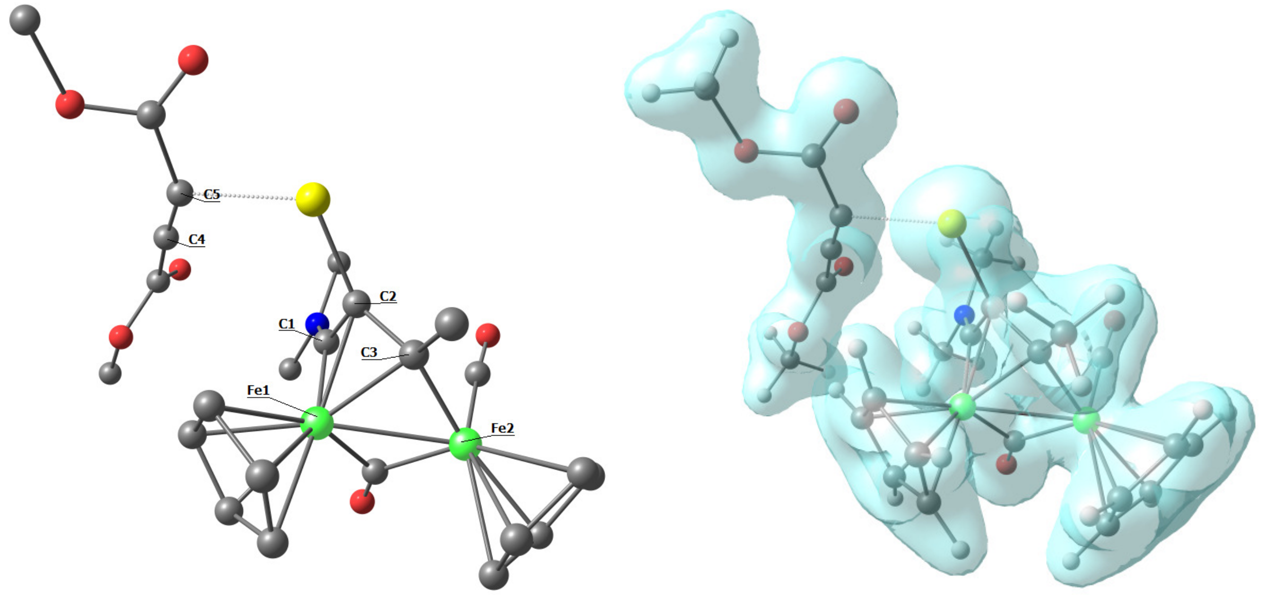

Figure S6. Left: DFT-optimized structure of **TS2** (C-PCM/ωB97X/def2-SVP, chloroform as continuous medium). Fe, green; Se, yellow; O, red; N, blue; C, grey. Hydrogen atoms are omitted for clarity. Selected computed bond lengths (Å): Fe1-C1 1.897, C1-C4 2.608, C1-N 1.298, C1-C2 1.435, C2-Se 1.917, Se-C5 1.948, C4-C5 1.323, C2-Fe1 2.070, C2-C3 1.419, C3-Fe1 2.021, C3-Fe2 1.965. Right: electron density surface, isovalue = 0.05 a.u.

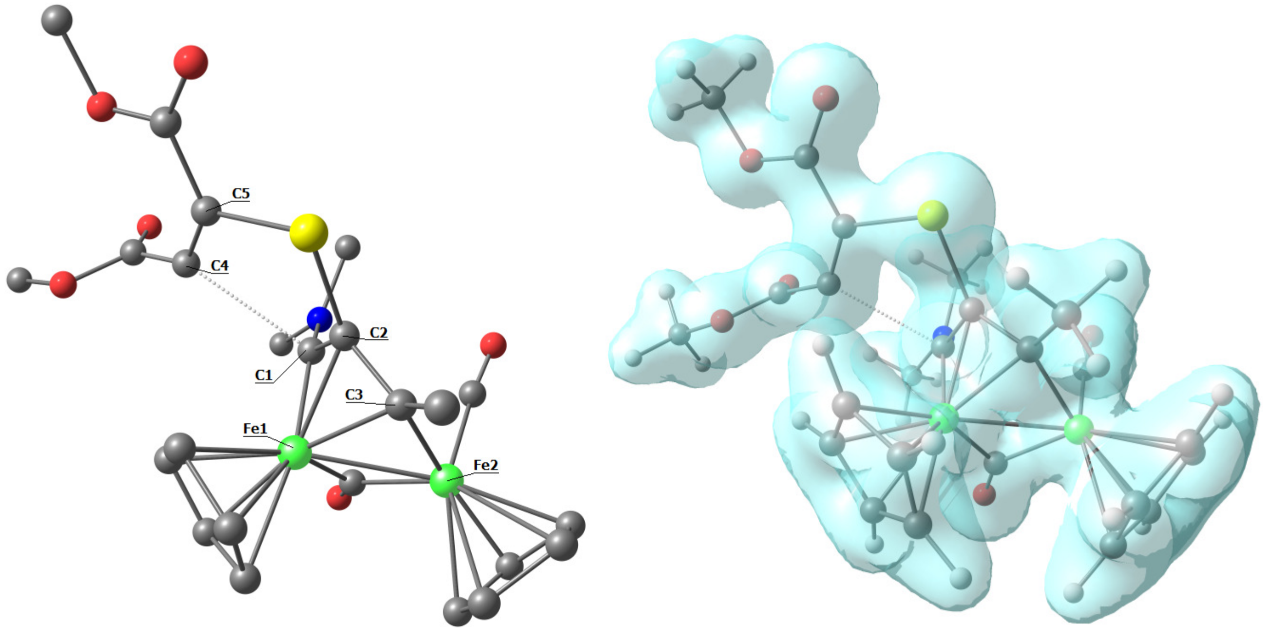

Figure S7. Left: DFT-optimized structure of **INT2** (C-PCM/ωB97X/def2-SVP, chloroform as continuous medium). Fe, green; Se, yellow; O, red; N, blue; C, grey. Hydrogen atoms are omitted for clarity. Selected computed bond lengths (Å): Fe1-C1 2.094, C1-C4 1.494, C1-N 1.443, C1-C2 1.463, C2-Se 1.925, Se-C5 1.880, C4-C5 1.349, C2-Fe1 2.030, C2-C3 1.411, C3-Fe1 2.020, C3-Fe2 1.955. Right: electron density surface, isovalue = 0.05 a.u.

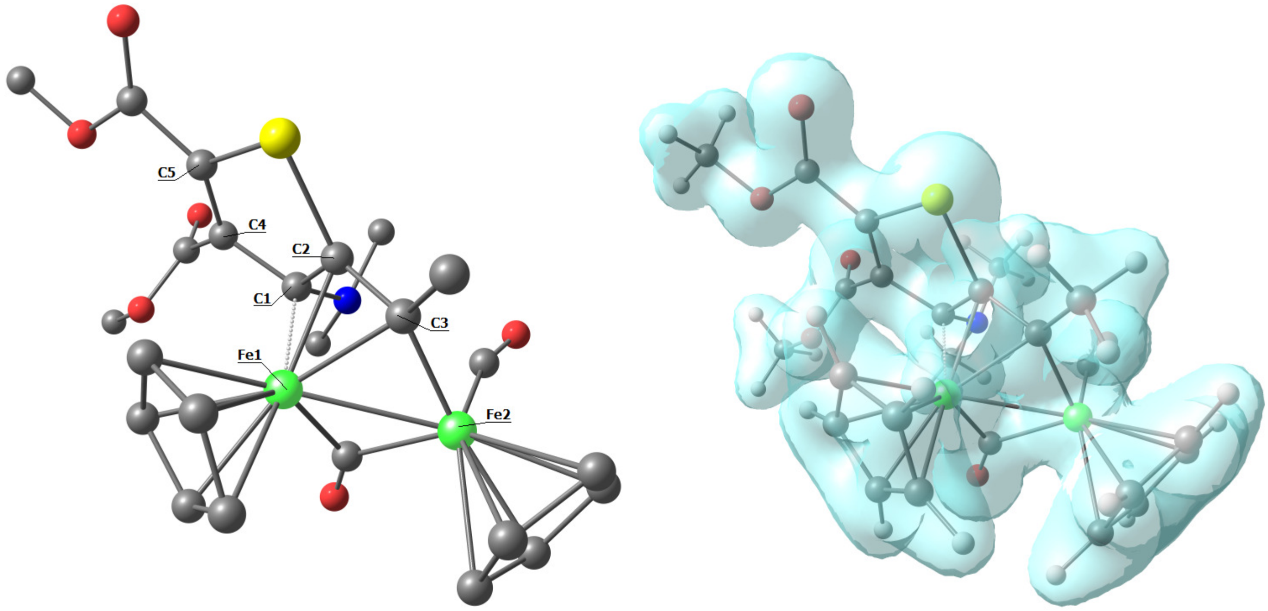

Figure S8. Left: DFT-optimized structure of **4a** (C-PCM/ωB97X/def2-SVP, chloroform as continuous medium). Fe, green; Se, yellow; O, red; N, blue; C, grey. Hydrogen atoms are omitted for clarity. Selected computed bond lengths (Å): Fe1-N 2.231, C1-C4 1.435, C1-N 1.448, C1-C2 1.375, C2-Se 1.867, Se-C5 1.860, C4-C5 1.373, C2-C3 1.472, C3-Fe1 1.954, C3-Fe2 2.011. Right: electron density surface, isovalue = 0.05 a.u.

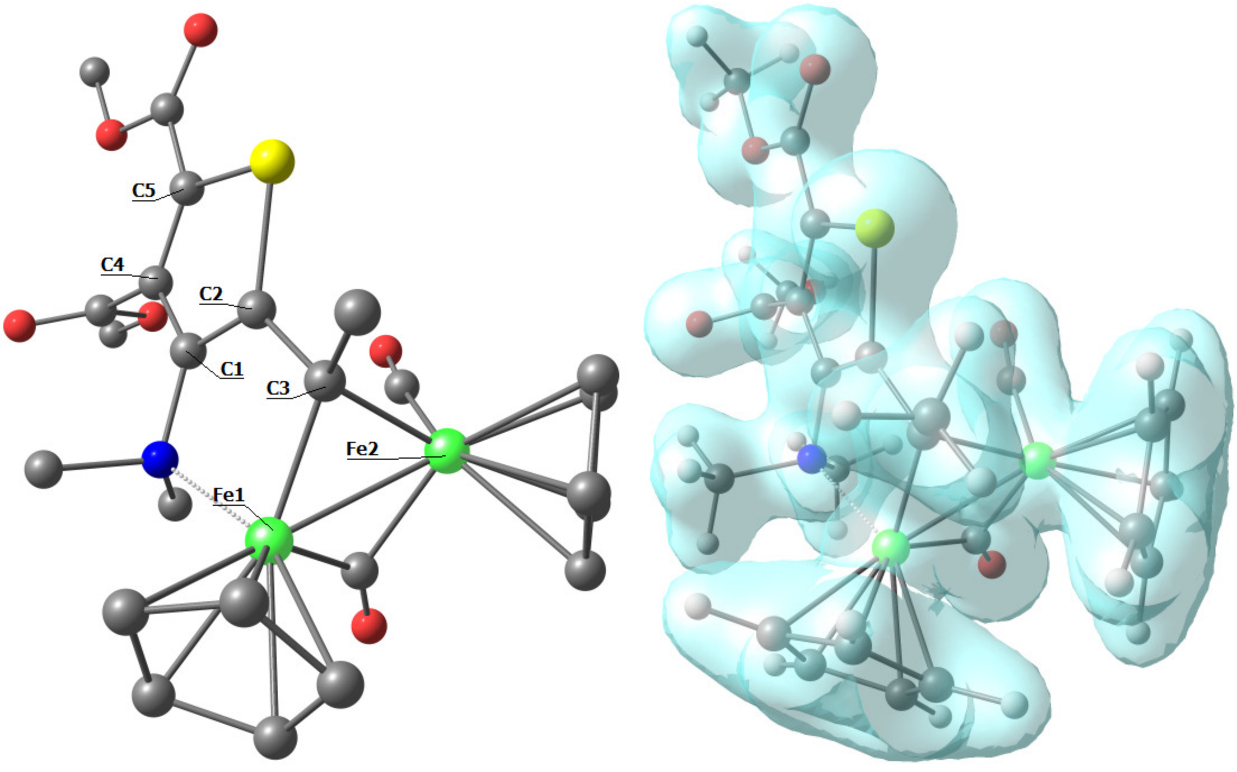

Figure S9. DFT-optimized structures of **4a** and **4a-H$_2$O** and Gibbs energy variations for the reaction of **4a** with water (C-PCM/ωB97X/def2-SVP, water as continuous medium). Fe, green; Se, yellow; O, red; N, blue; C, grey. The alkylidene carbon C^3 is evidenced. C-H hydrogen atoms are omitted for clarity.

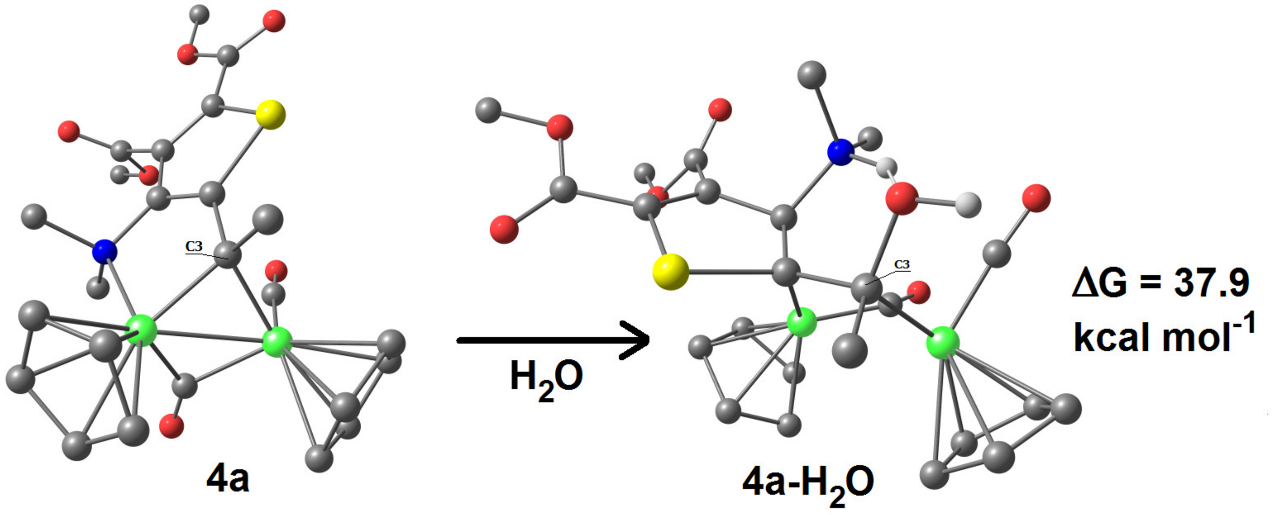

Table S2. Formal Electrode Potentials (V, *vs* $FeCp_2$) and Peak-to-Peak separations (mV) for the redox changes exhibited by **4a** and **5a** in 1,2-dme/$[N^nBu_4]PF_6$ 0.2 M.

			Oxidation		Reduction		
**Compound**	E_{a1}[b]	E_{a2}[b]	$E°'_3$	ΔE_3[a]	$E°'_4$	ΔE_4[a]	E_{c5}[b]
**4a**	+0.58	+0.47	-0.31	90	-2.06	420	
**5a**	+0.91				-1.79	130	-2.25

[a] Measured at 0.1 V s^{-1}. [b] Anodic or cathodic peak potential value for irreversible or quasi-reversible processes.

Figure S10. IR spectral changes of a solution of **4a** in 1,2-dme recorded in an OTTLE cell during: a) progressive increase of the potential from -0.3 to +0.6 V; b) reverse potential scan from +0.6 to -0.3 V. $[N^nBu_4]PF_6$ (0.2 mol dm^{-3}) as the supporting electrolyte. The absorptions related to the solvent and the supporting electrolyte have been subtracted.

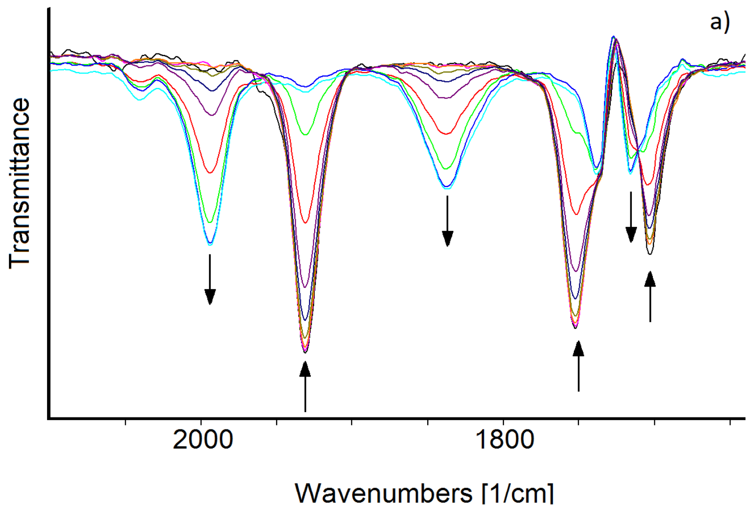

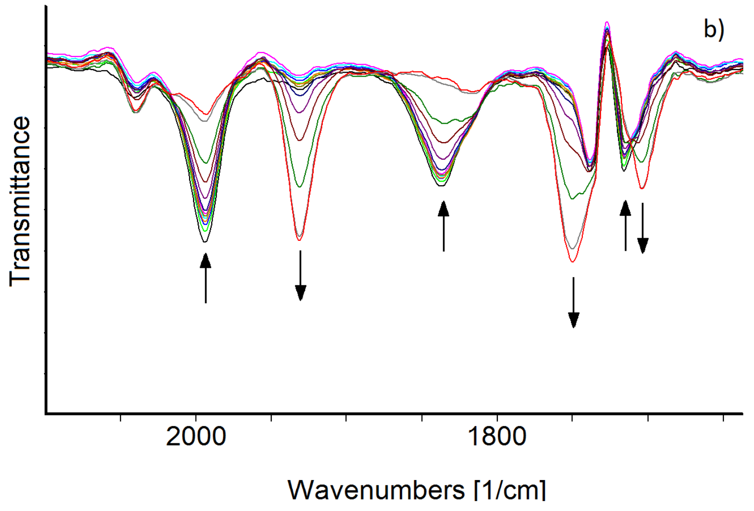

Figure S11. Comparison of the IR spectra in the ν_{CO} region of a solution of **4a** in 1,2-dme recorded in an OTTLE cell. Black line: IR spectrum of the solution before oxidation (**Figure S10a**); red line: IR spectrum of the solution at the end of the reverse potential scan (**Figure S10b**). $[N^nBu_4]PF_6$ (0.2 mol dm^{-3}) as the supporting electrolyte. The absorptions of the solvent and the supporting electrolyte have been subtracted.

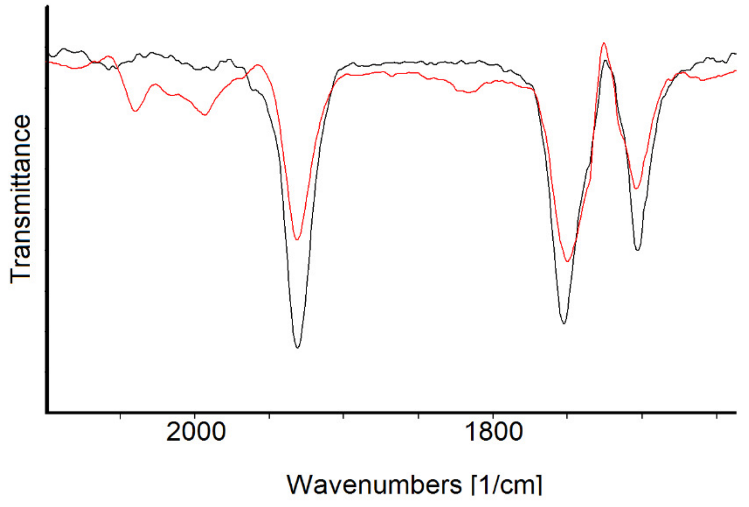

Figure S12. IR spectral changes of a solution of **4a** in 1,2-dme/H_2O recorded in an OTTLE cell during the progressive increase of the potential from -0.3 to +0.6 V. $[N^nBu_4]PF_6$ (0.2 mol dm^{-3}) as the supporting electrolyte. The absorptions related to the solvent and the supporting electrolyte have been subtracted.

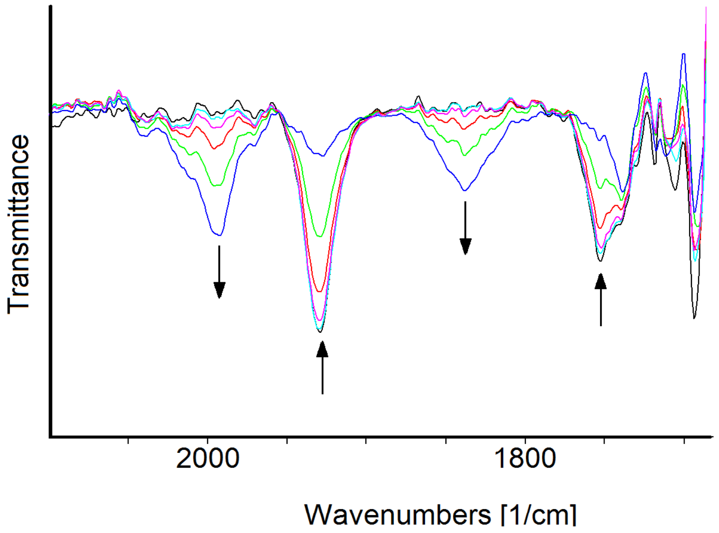

Figure S13. Comparison of IR spectra in the ν_{CO} region of a solution of **4a** in 1,2-dme/H_2O recorded in an OTTLE cell. Black line: IR spectrum of the solution before oxidation (**Figure S12**); red line: IR spectrum of the solution at the end of the reverse potential scan. $[N^nBu_4]PF_6$ (0.2 mol dm^{-3}) as the supporting electrolyte. The absorptions of the solvent and the supporting electrolyte have been subtracted.

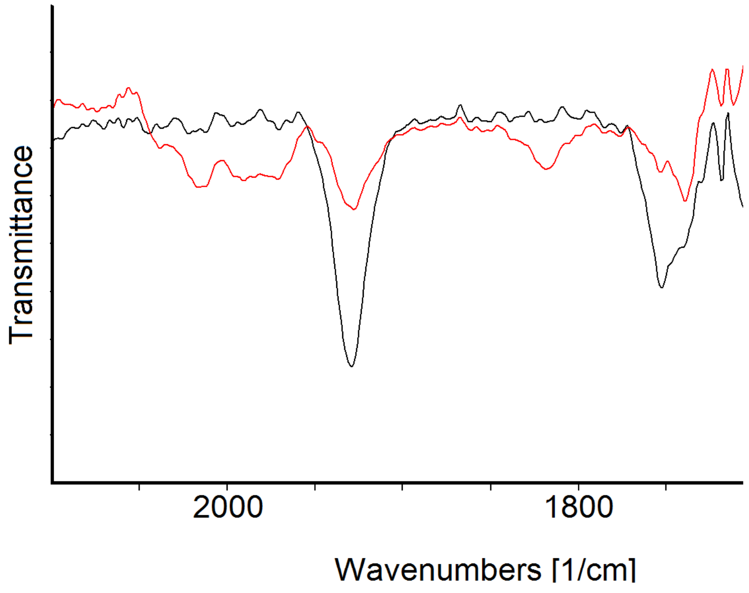

Figure S14. ^{1}H NMR spectrum (401 MHz, acetone-d6) of [**2b**]CF_3SO_3.

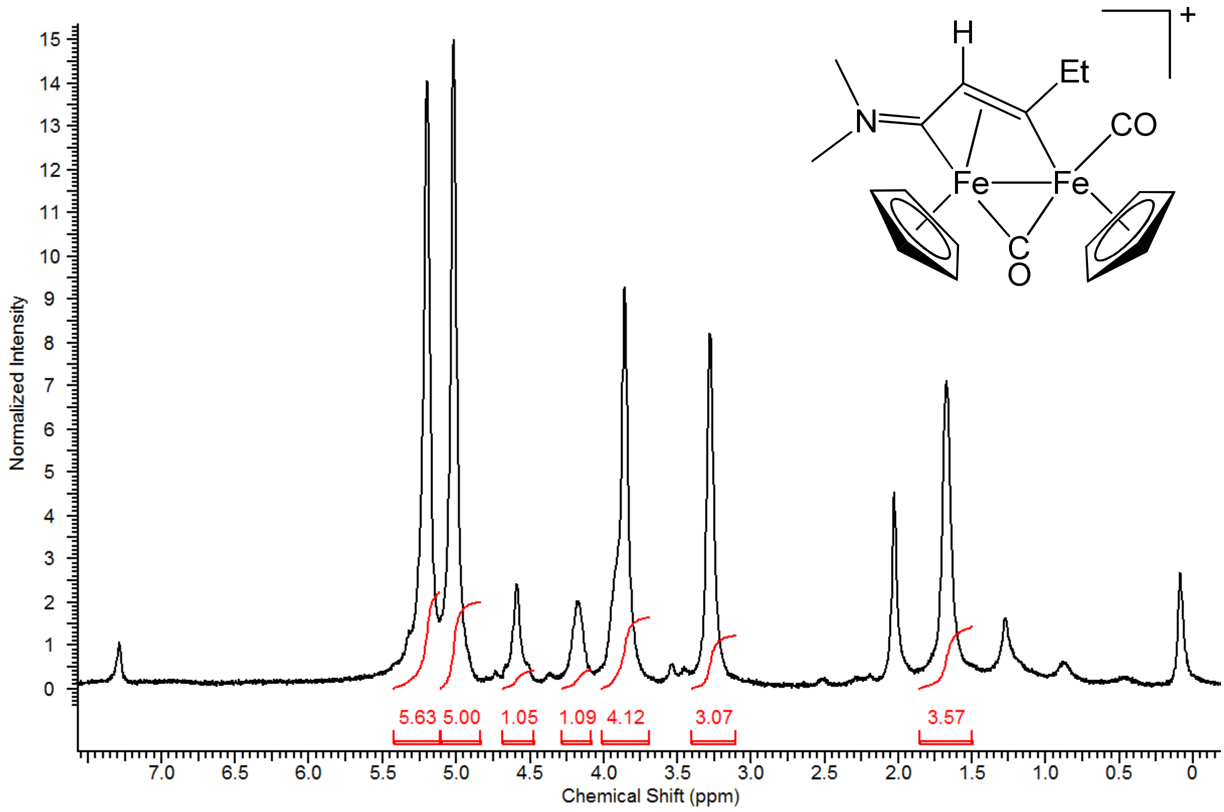

Figure S15. ^{13}C{^{1}H} NMR spectrum (101 MHz, acetone-d6) of [**2b**]CF_3SO_3.

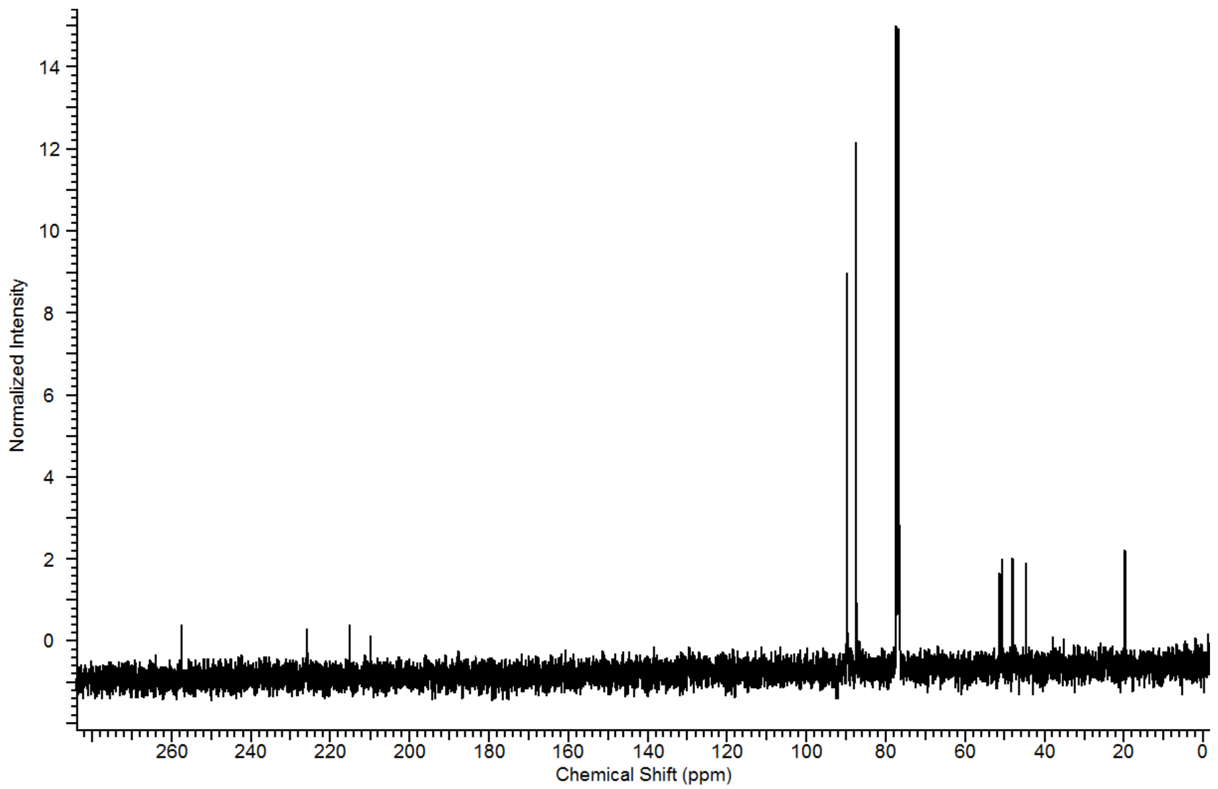

Figure S16. ^{1}H NMR spectrum (401 MHz, acetone-d_6) of [**2c**]CF_3SO_3.

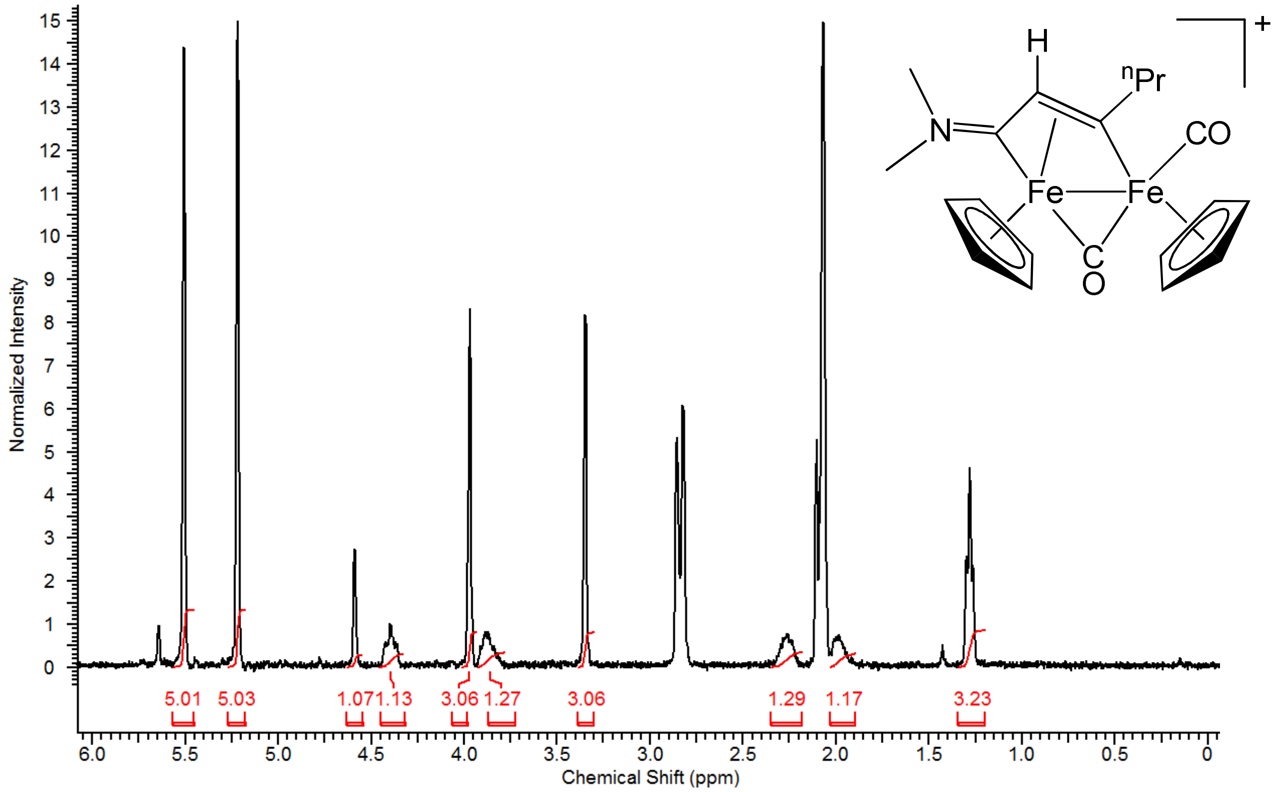

Figure S17. $^{13}C\{^{1}H\}$ NMR spectrum (101 MHz, acetone-d_6) of [**2c**]CF_3SO_3.

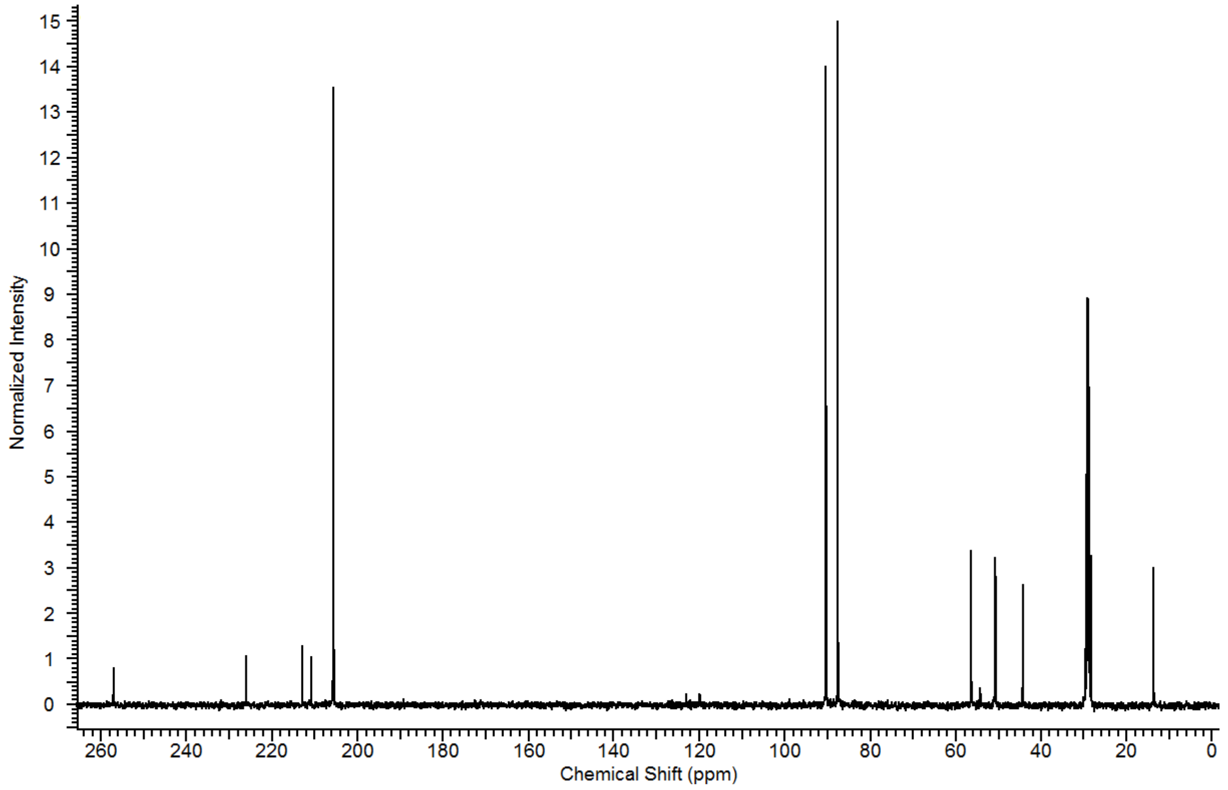

Figure S18. ^{1}H NMR spectrum (401 MHz, $CDCl_3$) of [**2d**]CF_3SO_3.

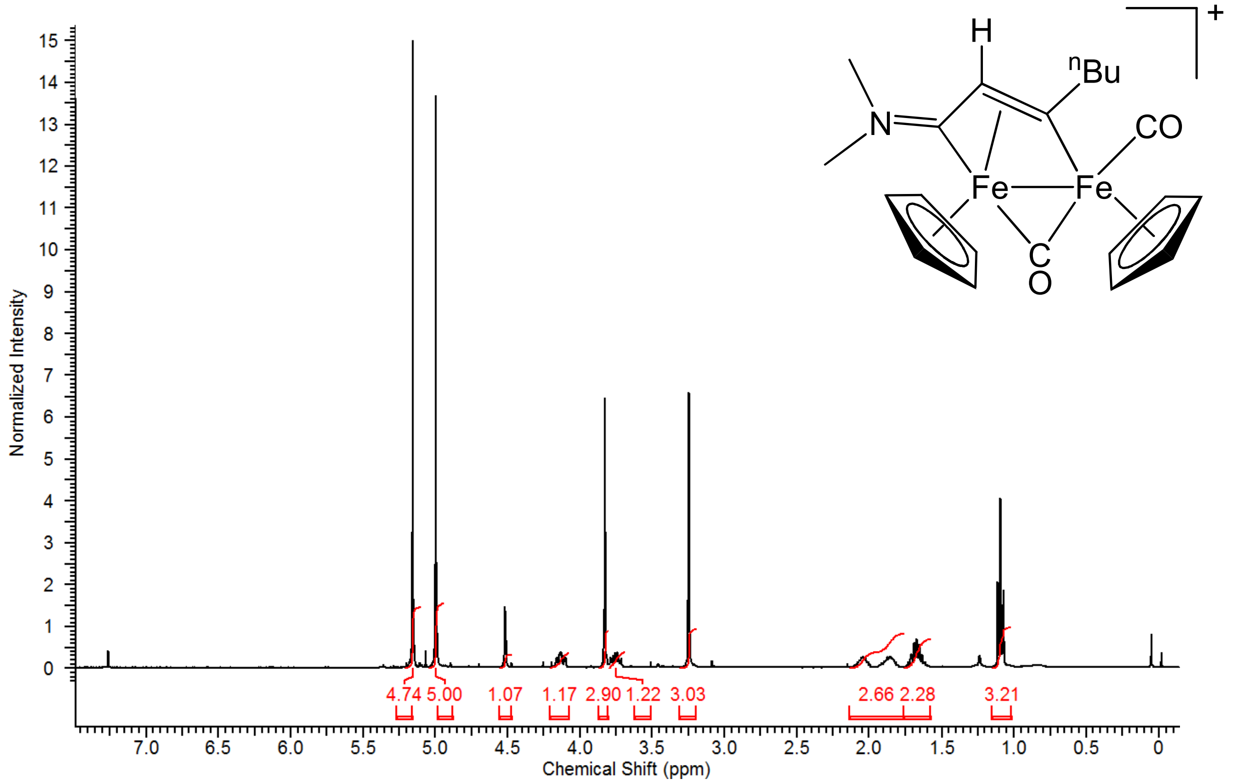

Figure S19. $^{13}C\{^{1}H\}$ NMR spectrum (101 MHz, $CDCl_3$) of [**2d**]CF_3SO_3.

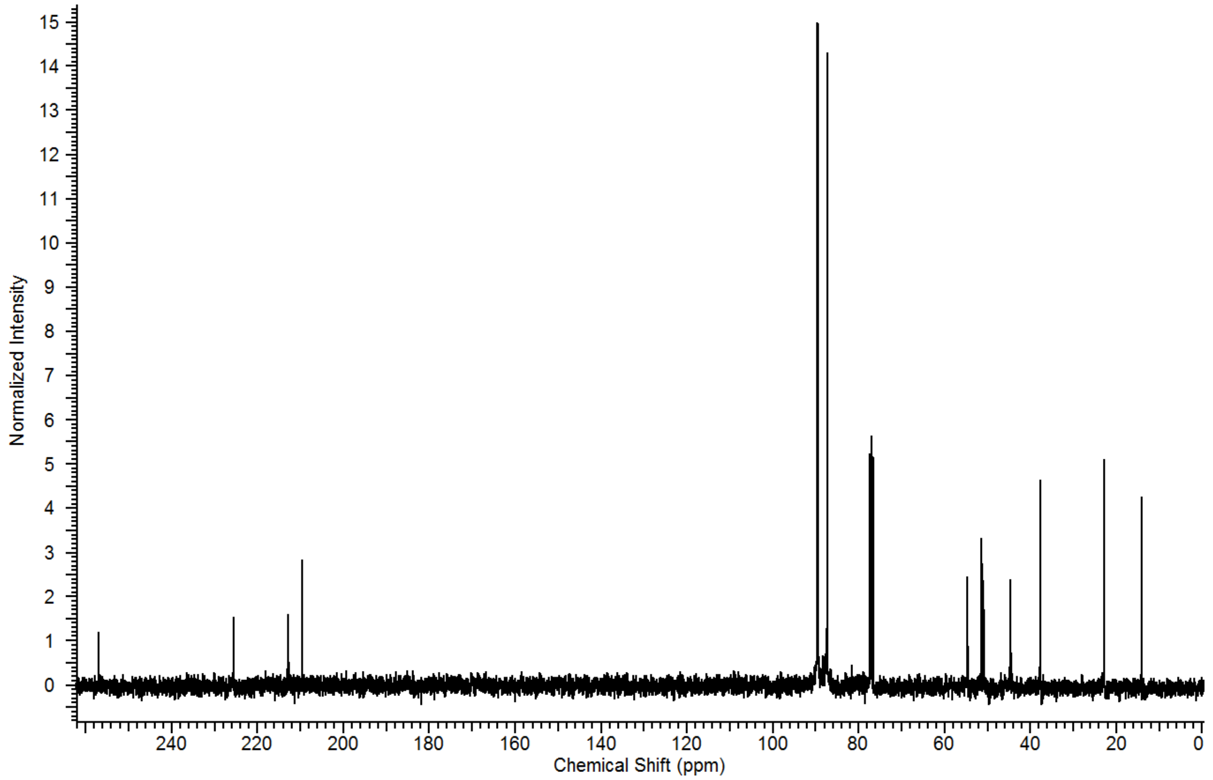

Figure S20. ^{1}H NMR spectrum (401 MHz, $CDCl_3$) of **3a**.

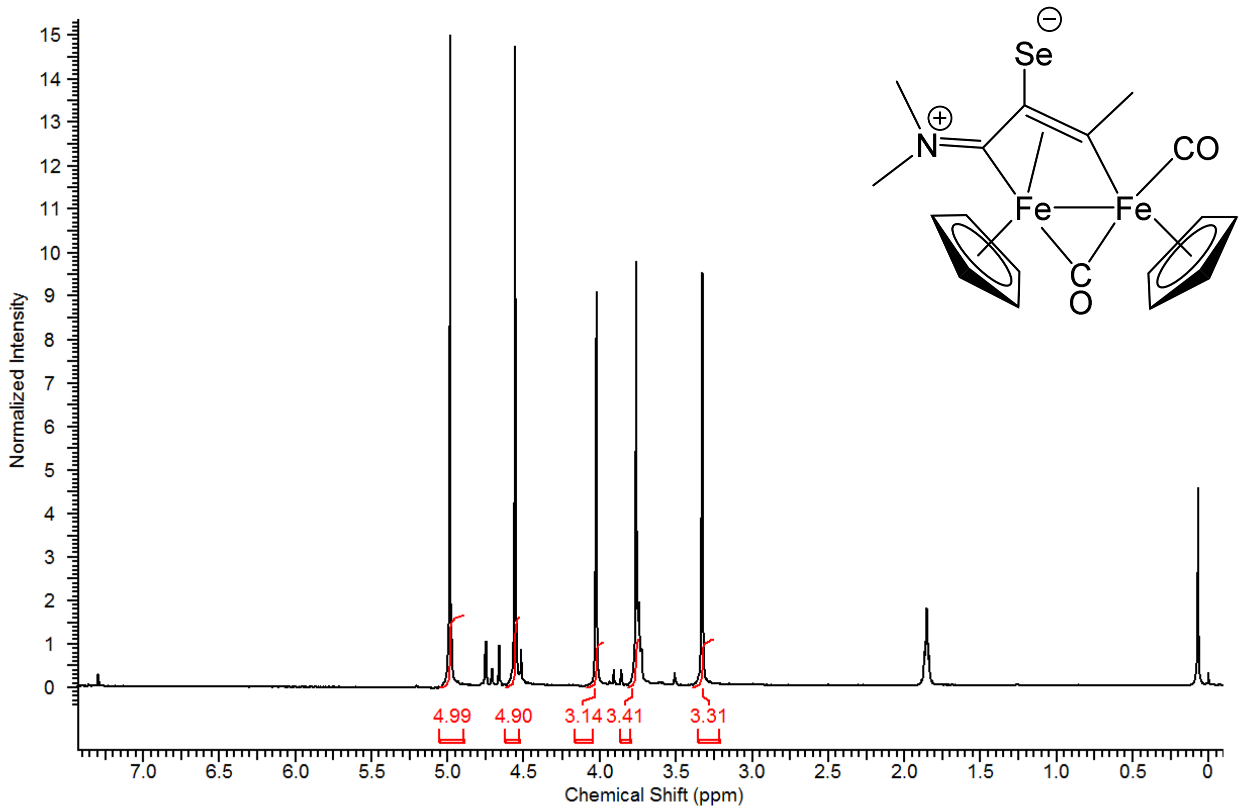

Figure S21. ^{13}C{^{1}H} NMR spectrum (101 MHz, $CDCl_3$) of **3a**.

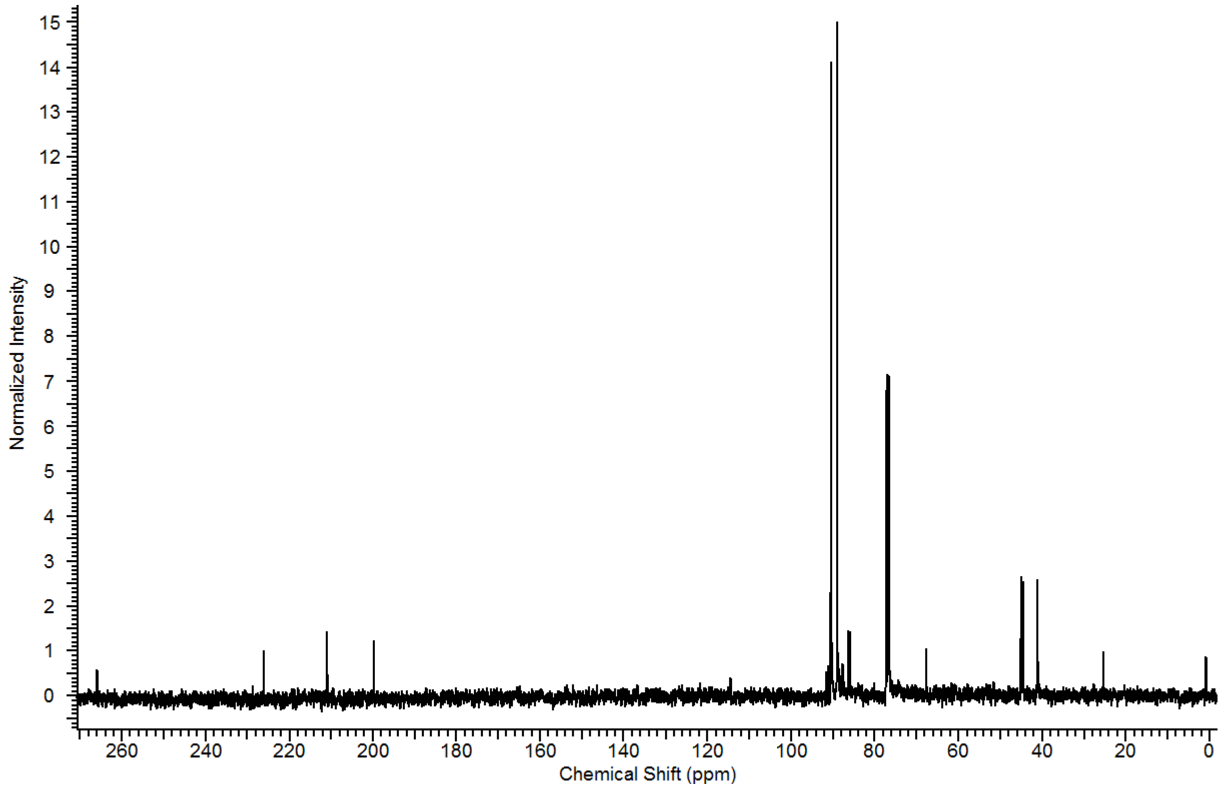

Figure S22. ^{77}Se NMR spectrum (76 MHz, $CDCl_3$) of **3a**.

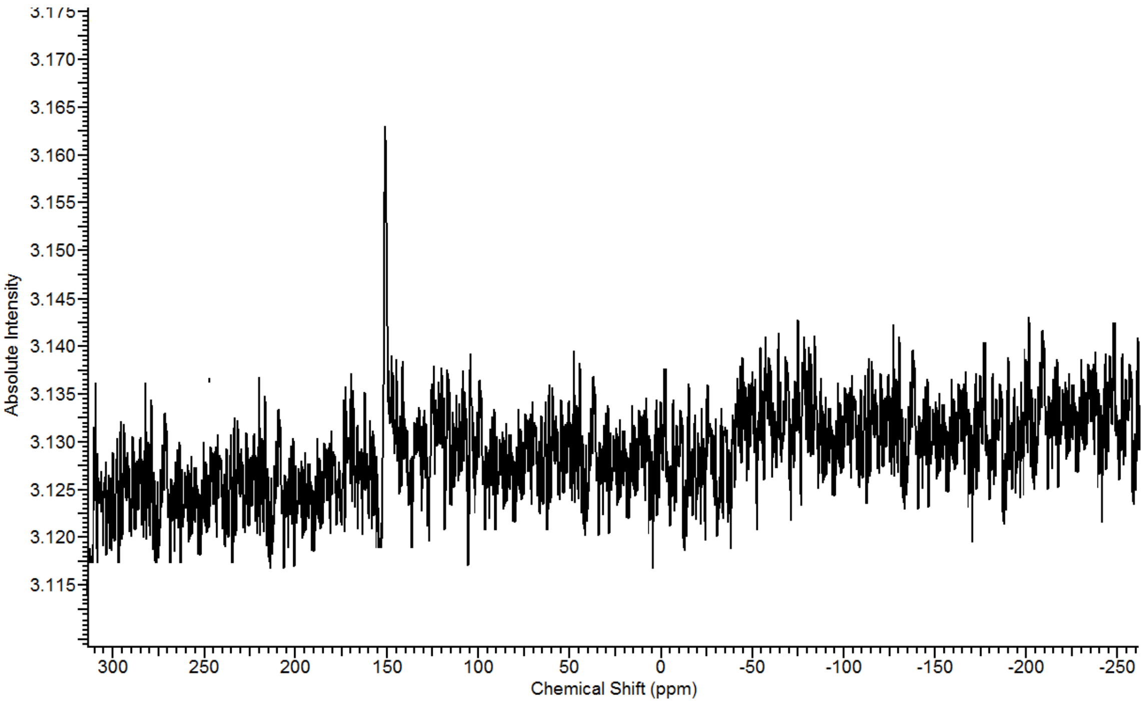

Figure S23. ^{1}H NMR spectrum (401 MHz, $CDCl_3$) of **3b**.

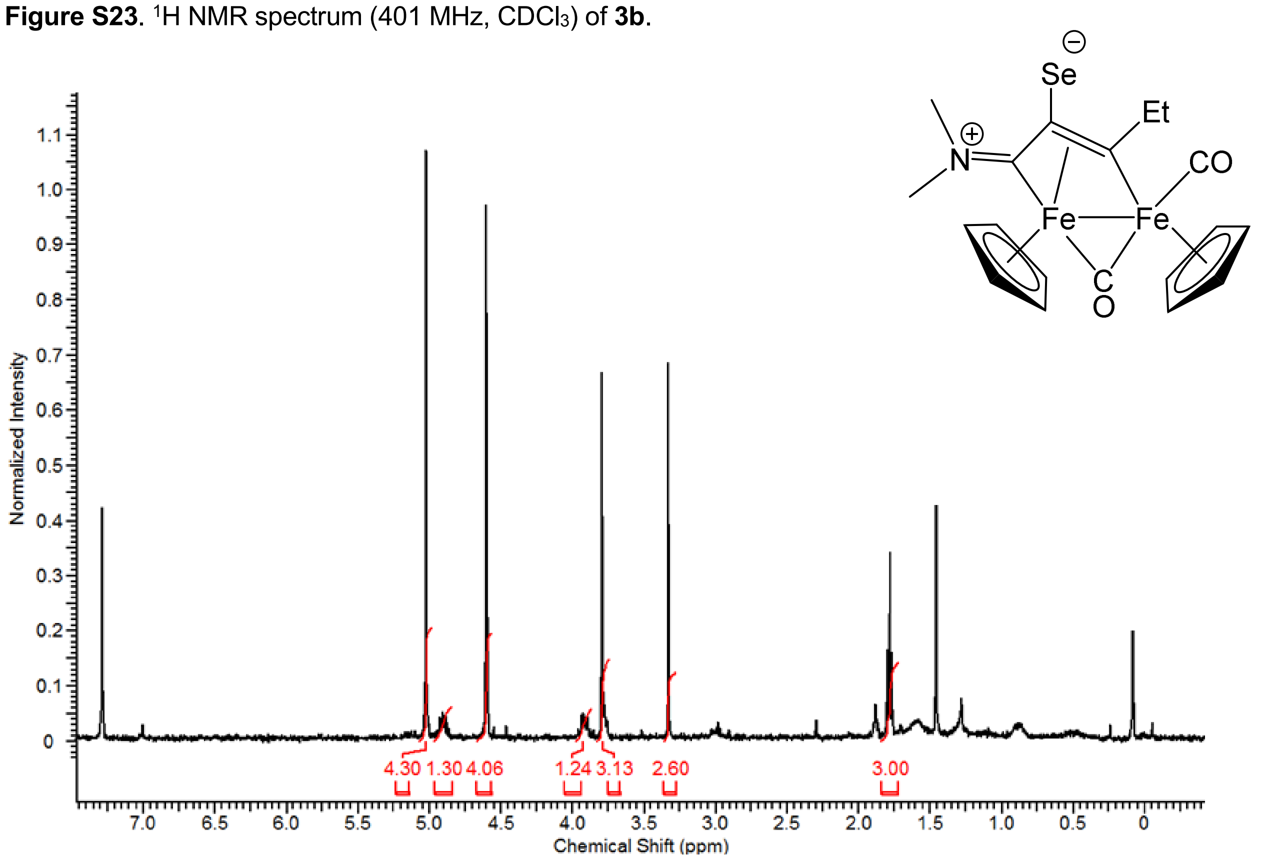

Figure S24. $^{13}C\{^{1}H\}$ NMR spectrum (101 MHz, $CDCl_3$) of **3b**.

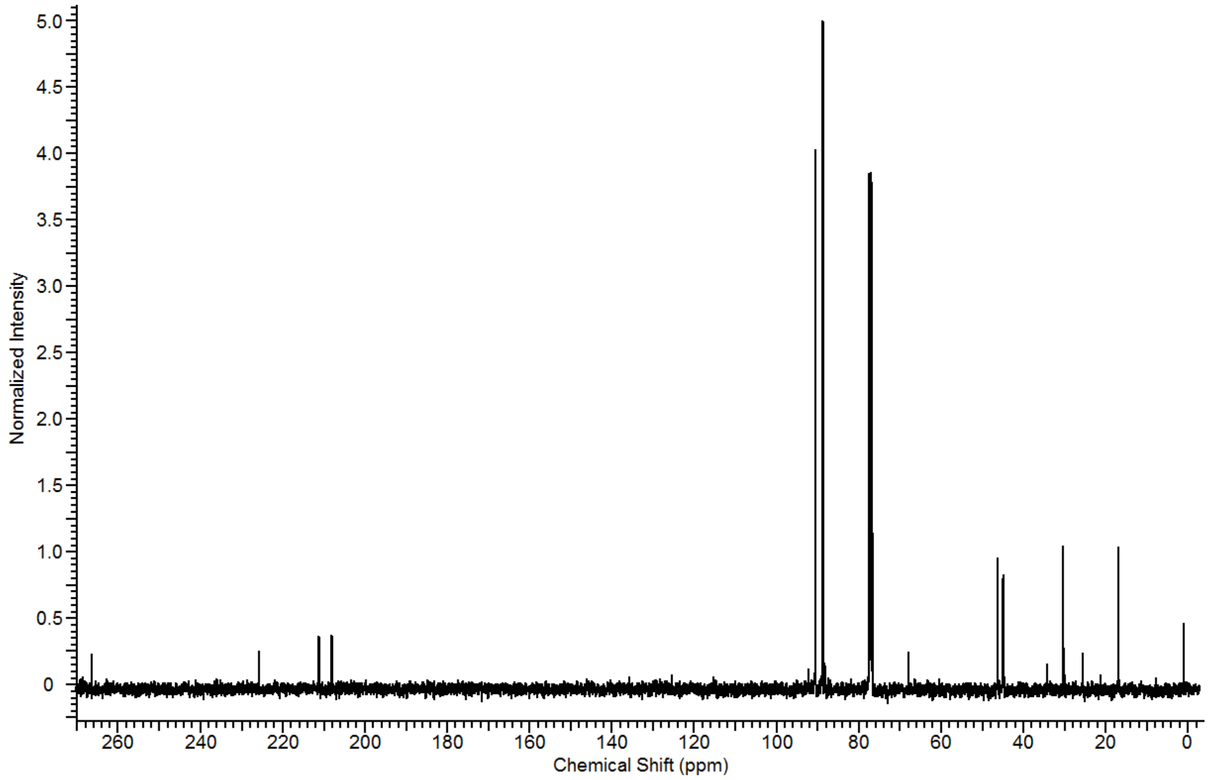

Figure S25. ^{77}Se NMR spectrum (76 MHz, $CDCl_3$) of **3b**.

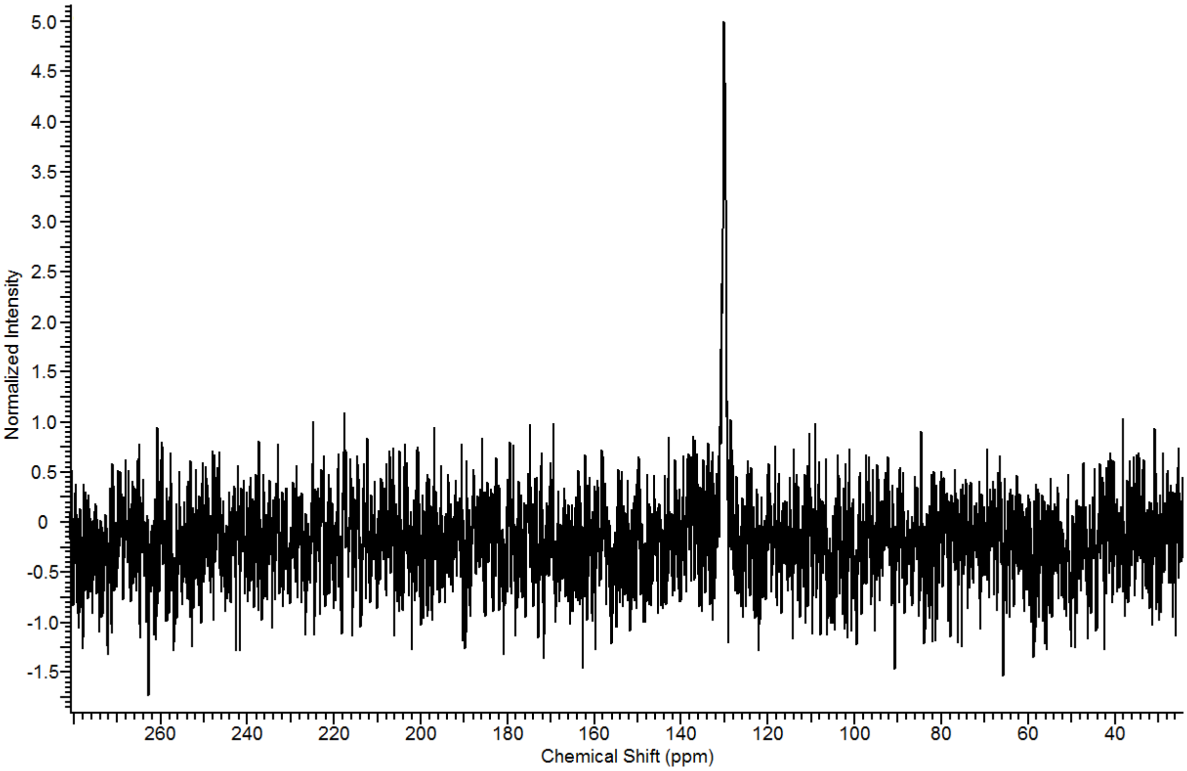

Figure S26. ^{1}H NMR spectrum (401 MHz, $CDCl_3$) of **3c**.

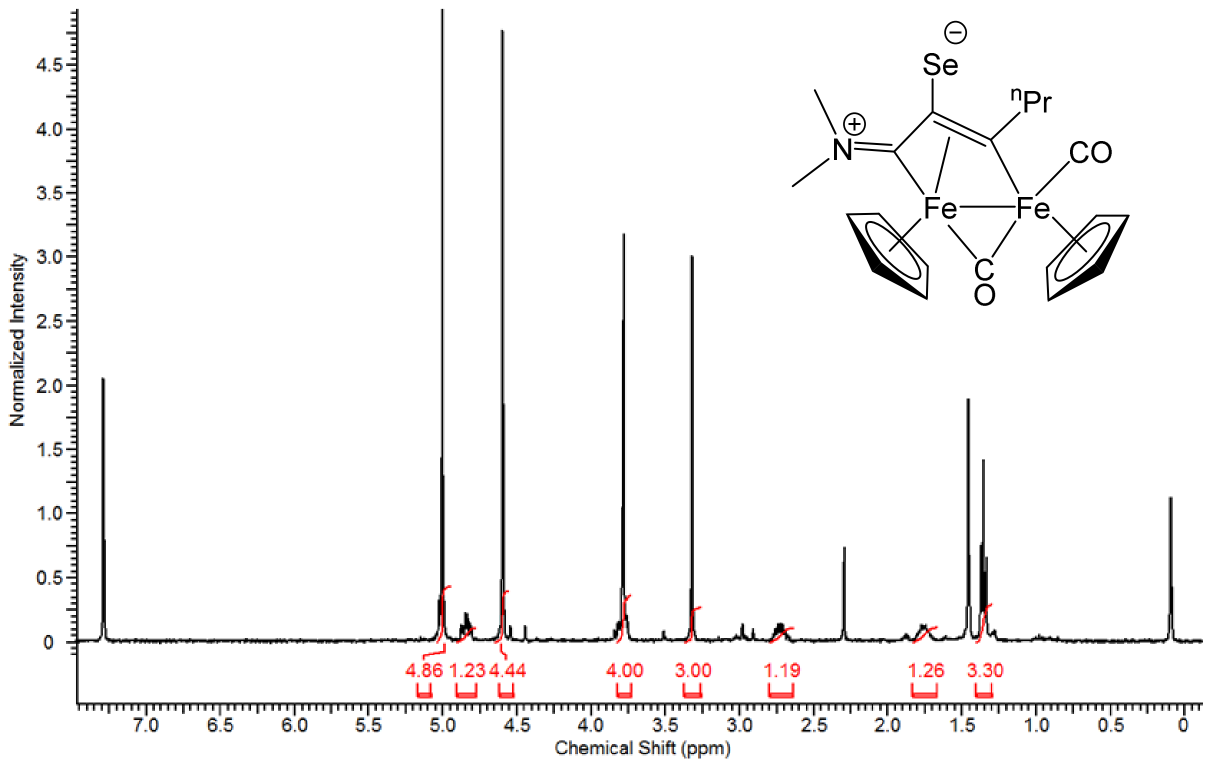

Figure S27. $^{13}C\{^{1}H\}$ NMR spectrum (101 MHz, $CDCl_3$) of **3c**.

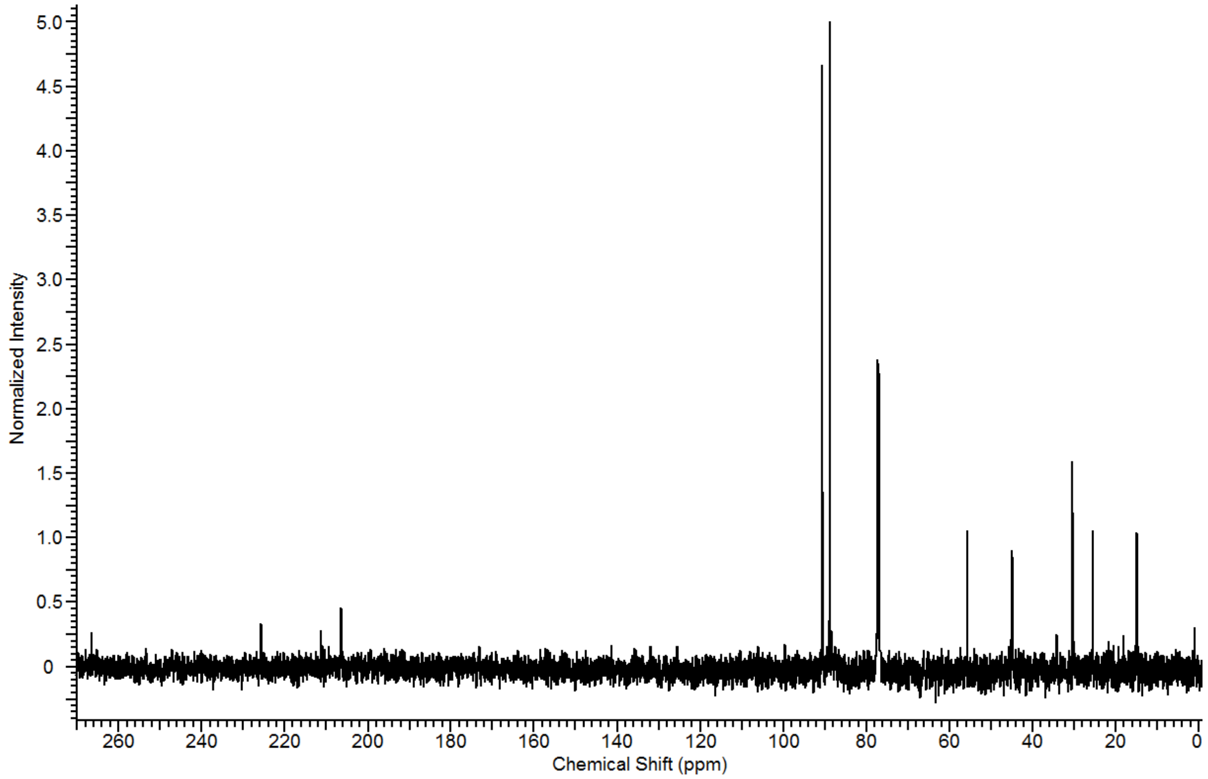

Figure S28. ^{77}Se NMR spectrum (76 MHz, $CDCl_3$) of **3c**.

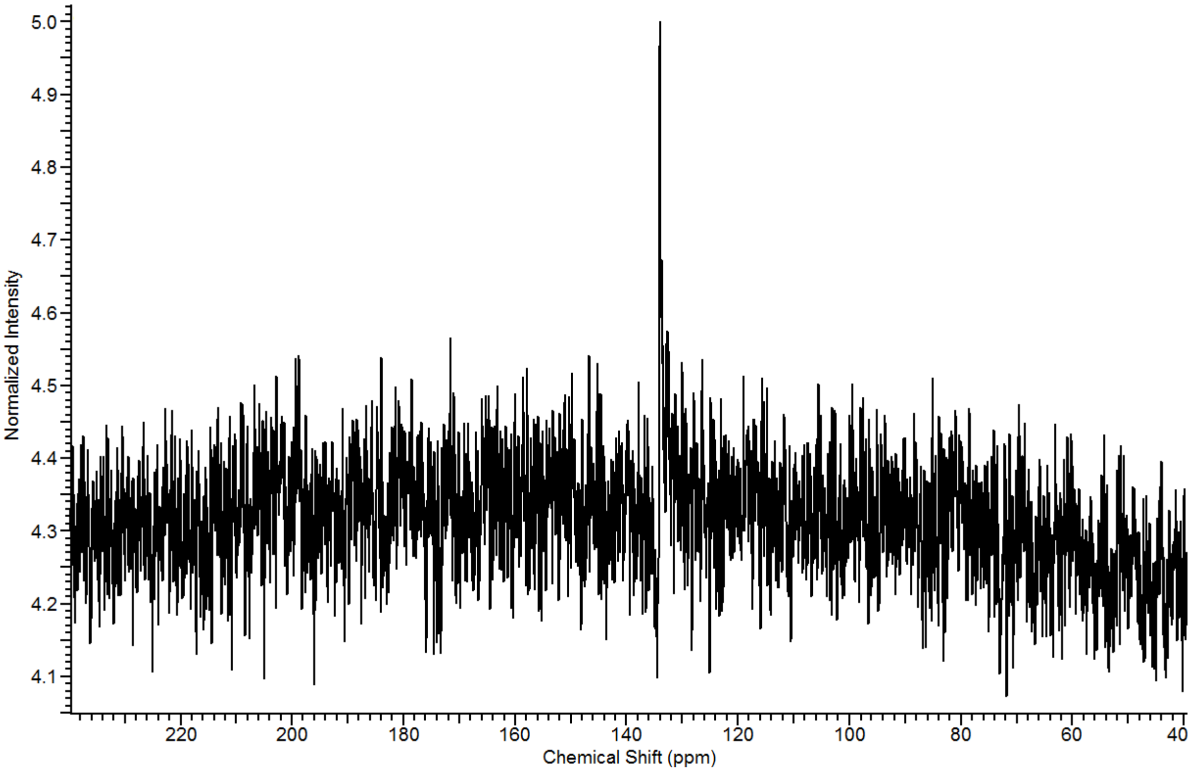

Figure S29. ^{1}H NMR spectrum (401 MHz, $CDCl_3$) of **4a**.

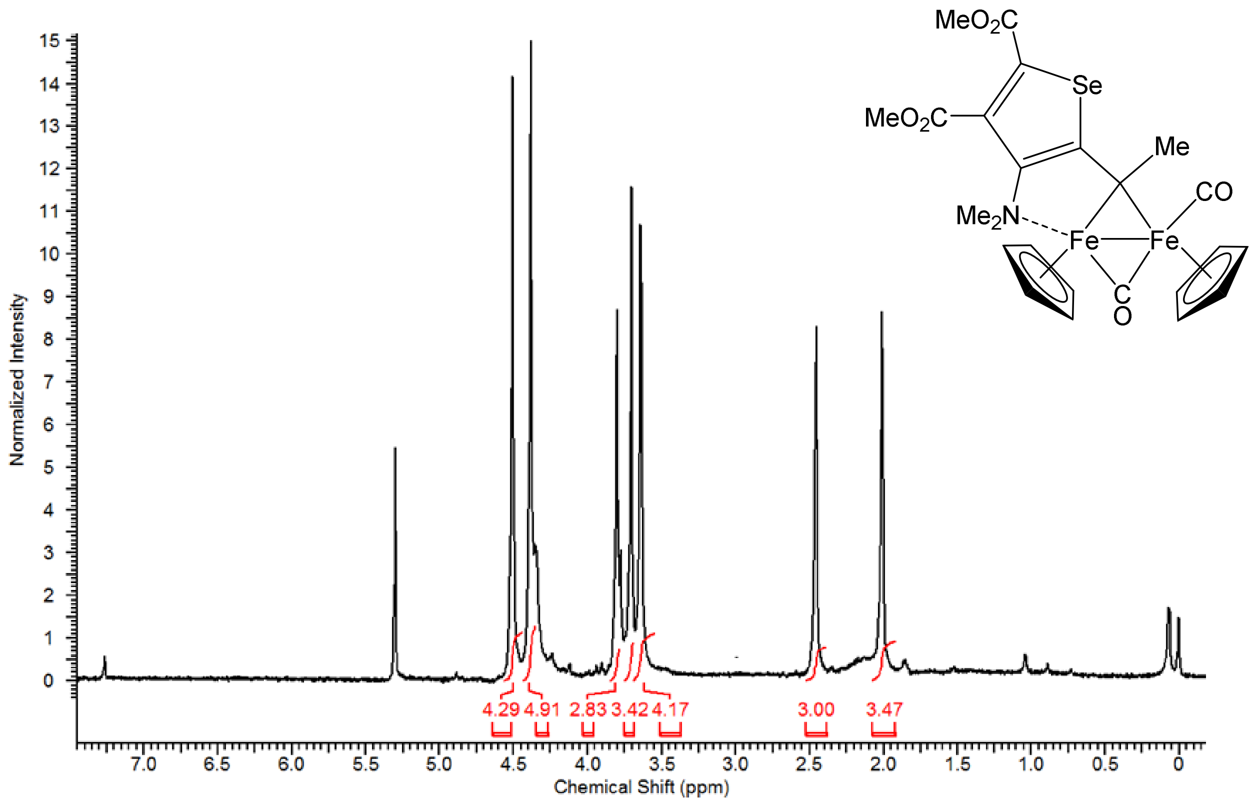

Figure S30. ^{13}C{^{1}H} NMR spectrum (101 MHz, $CDCl_3$) of **4a**.

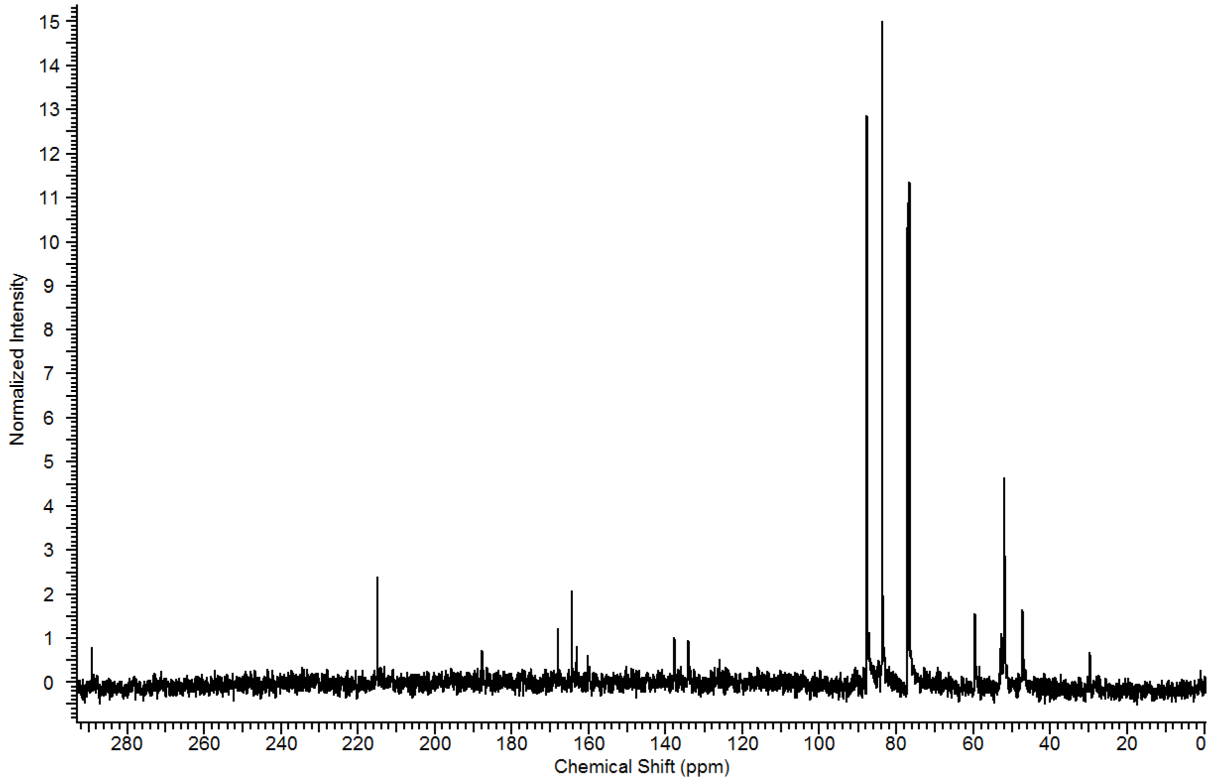

Figure S31. ^{77}Se NMR spectrum (76 MHz, $CDCl_3$) of **4a**.

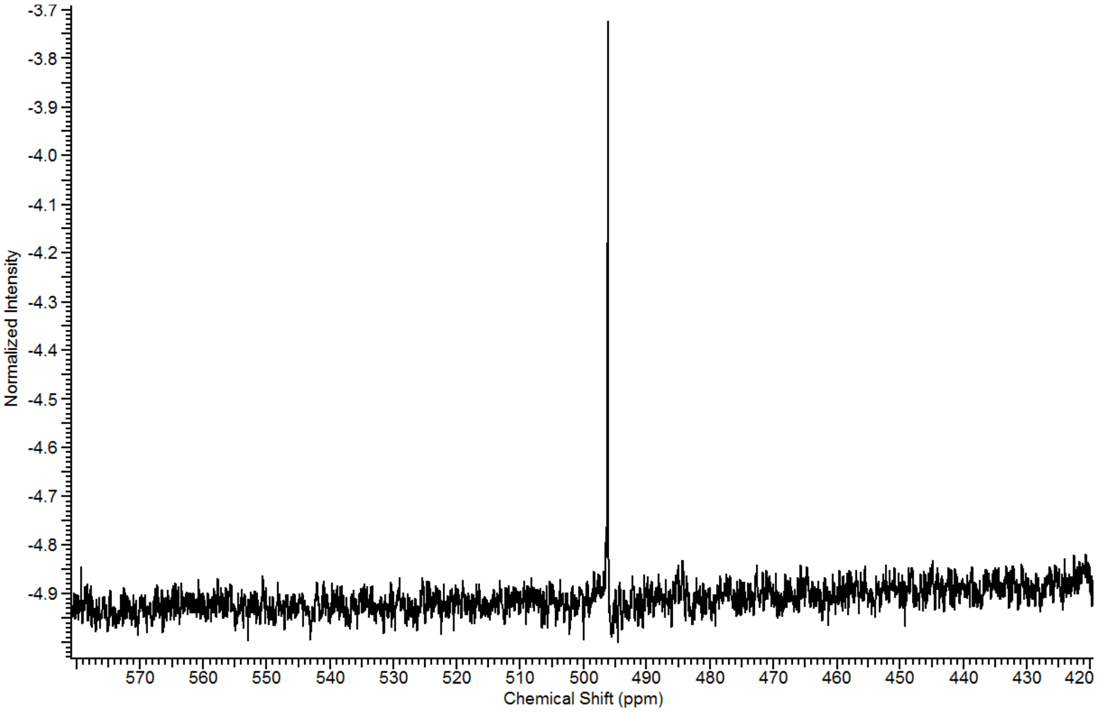

Figure S32. ^{1}H NMR spectrum (401 MHz, $CDCl_3$) of **4b**.

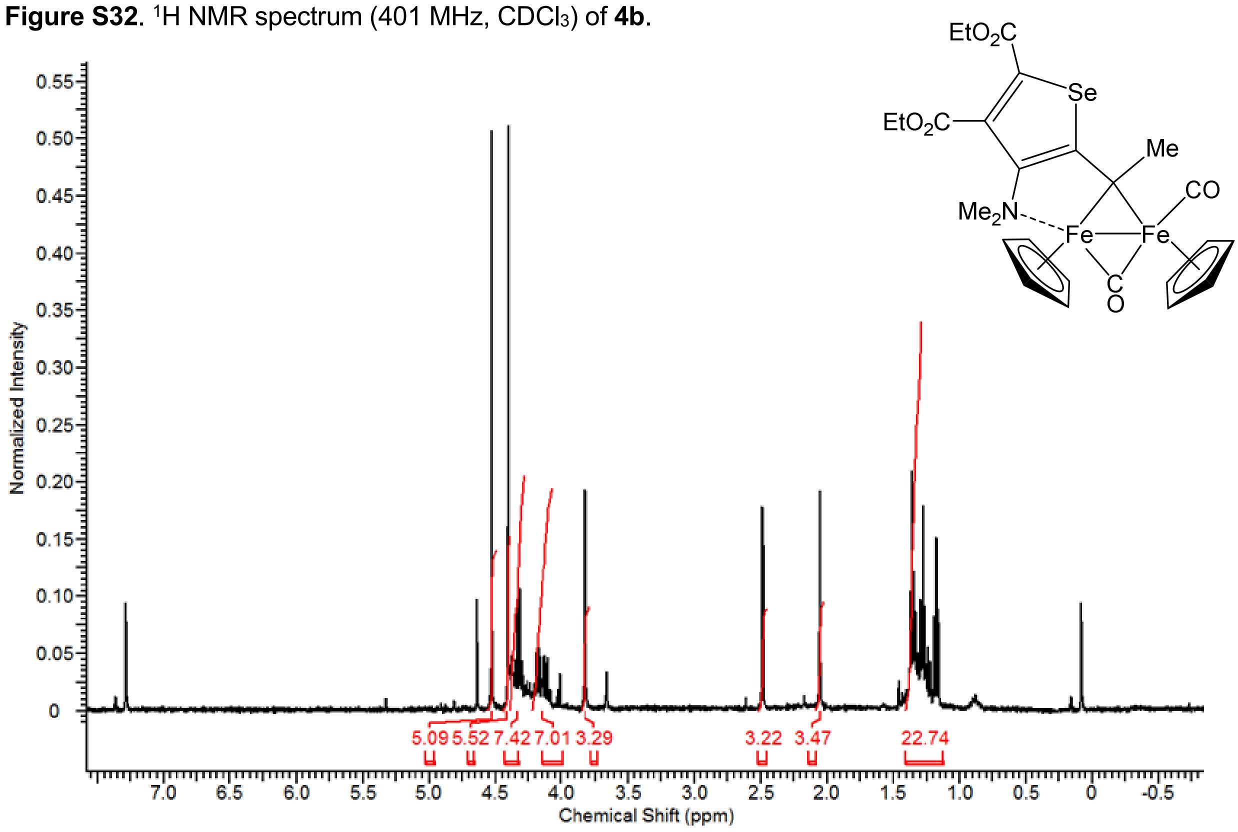

Figure S33. $^{13}C\{^{1}H\}$ NMR spectrum (101 MHz, $CDCl_3$) of **4b.**

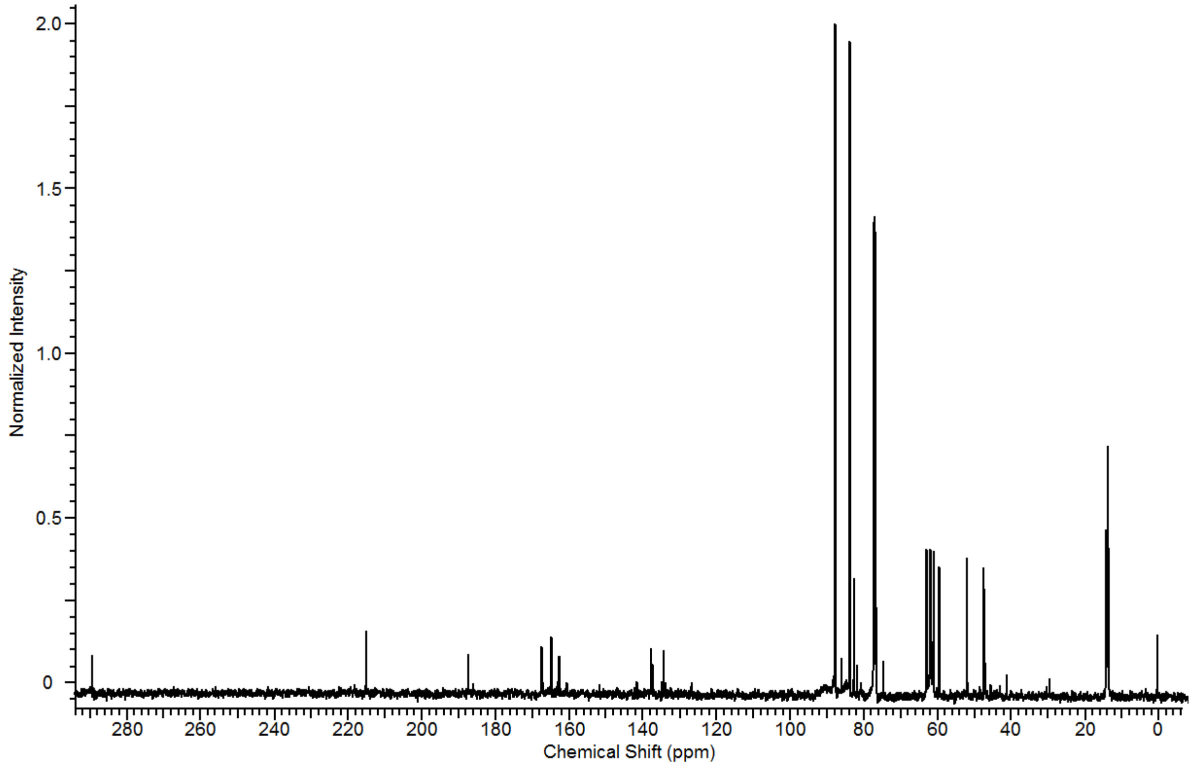

Figure S34. ^{77}Se NMR spectrum (76 MHz, $CDCl_3$) of **4b**.

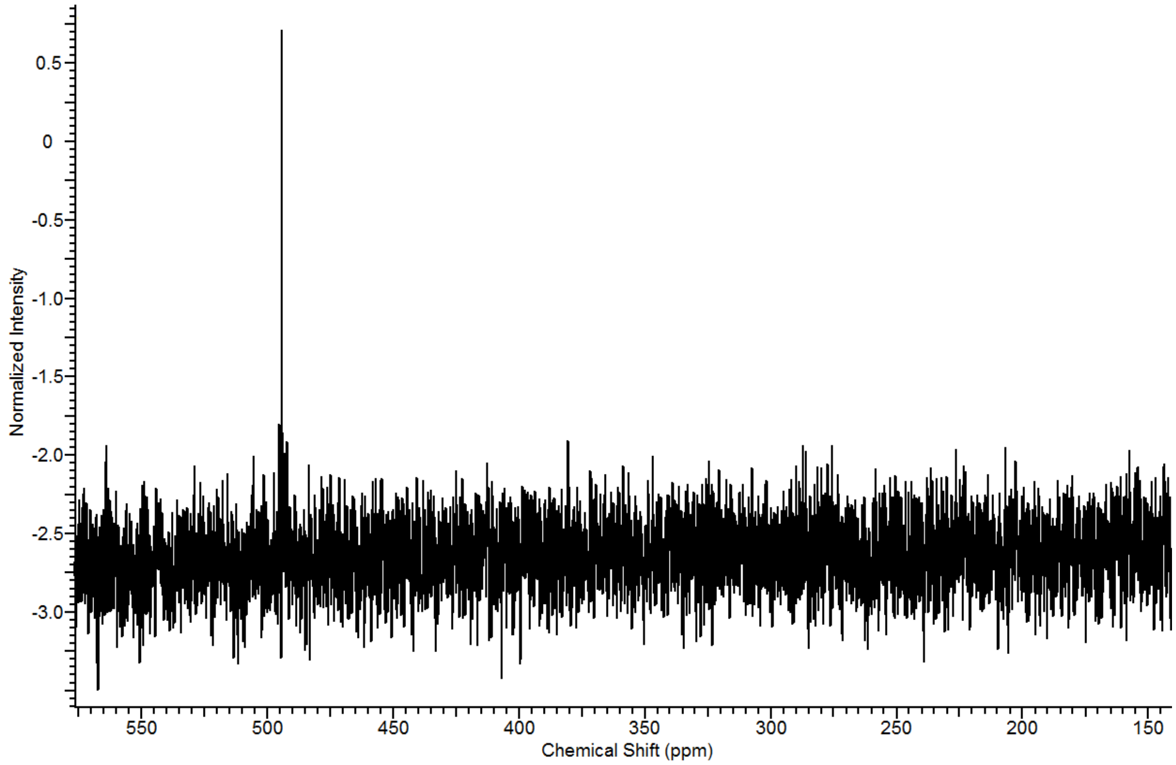

Figure S35. ^{1}H NMR spectrum (401 MHz, $CDCl_3$) of **4c**.

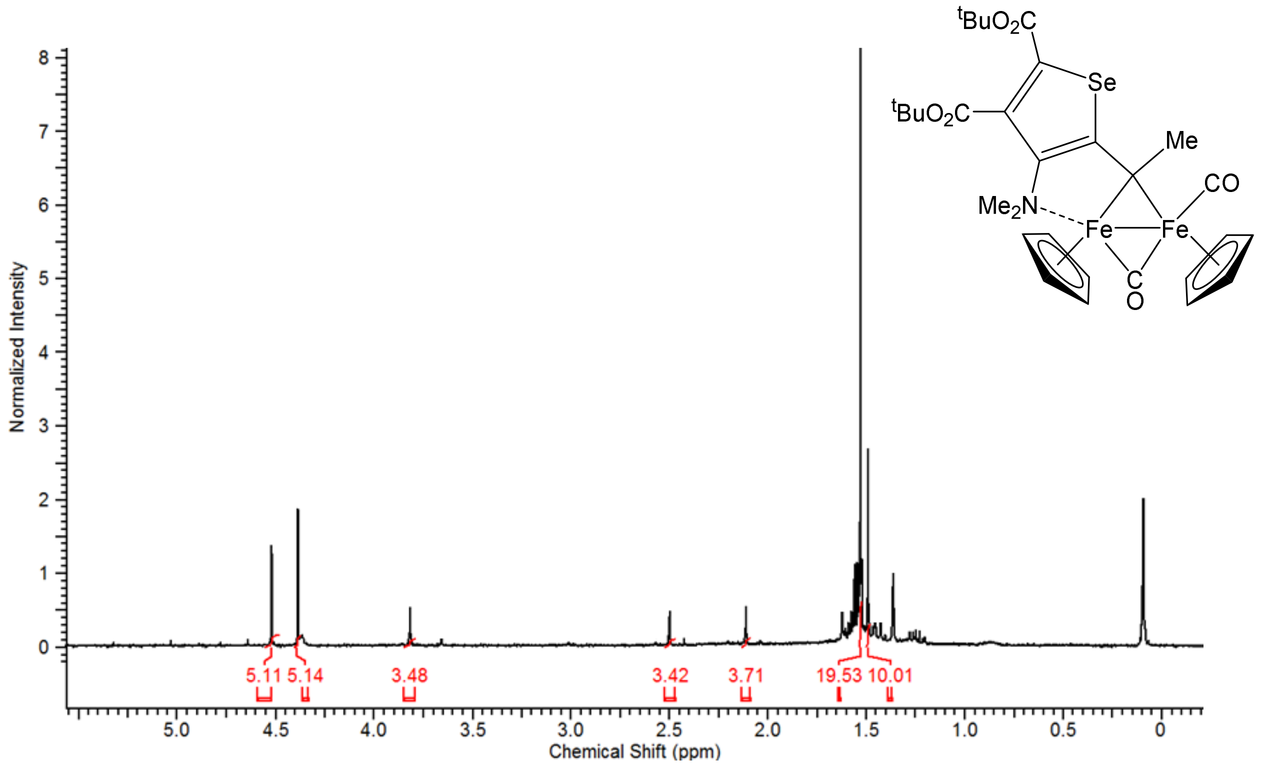

Figure S36. ^{13}C{^{1}H} NMR spectrum (101 MHz, $CDCl_3$) of **4c**.

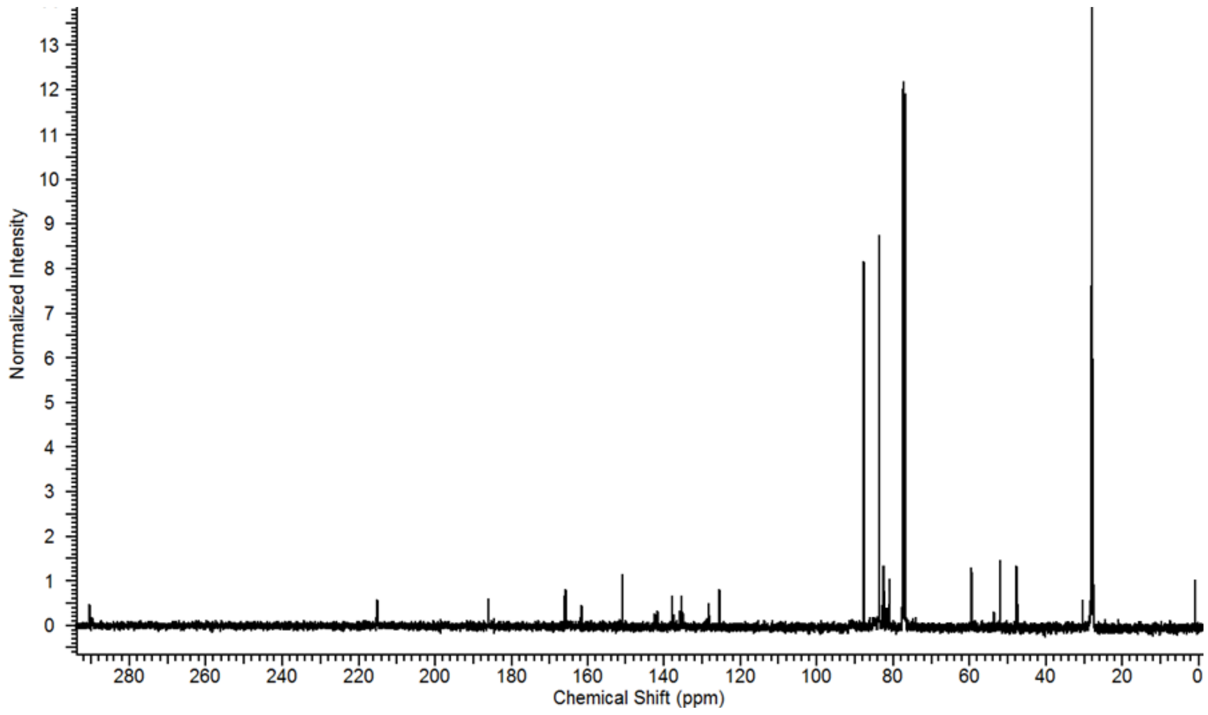

Figure S37. ^{77}Se NMR spectrum (76 MHz, $CDCl_3$) of **4c**.

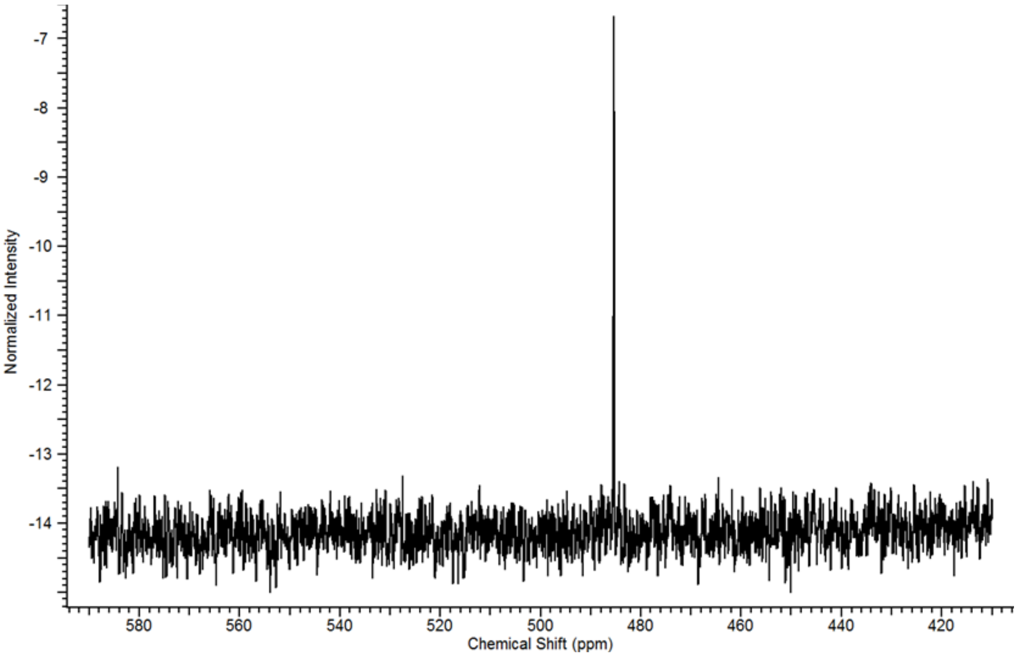

Figure S38. ^{1}H NMR spectrum (401 MHz, $CDCl_3$) of **4d**.

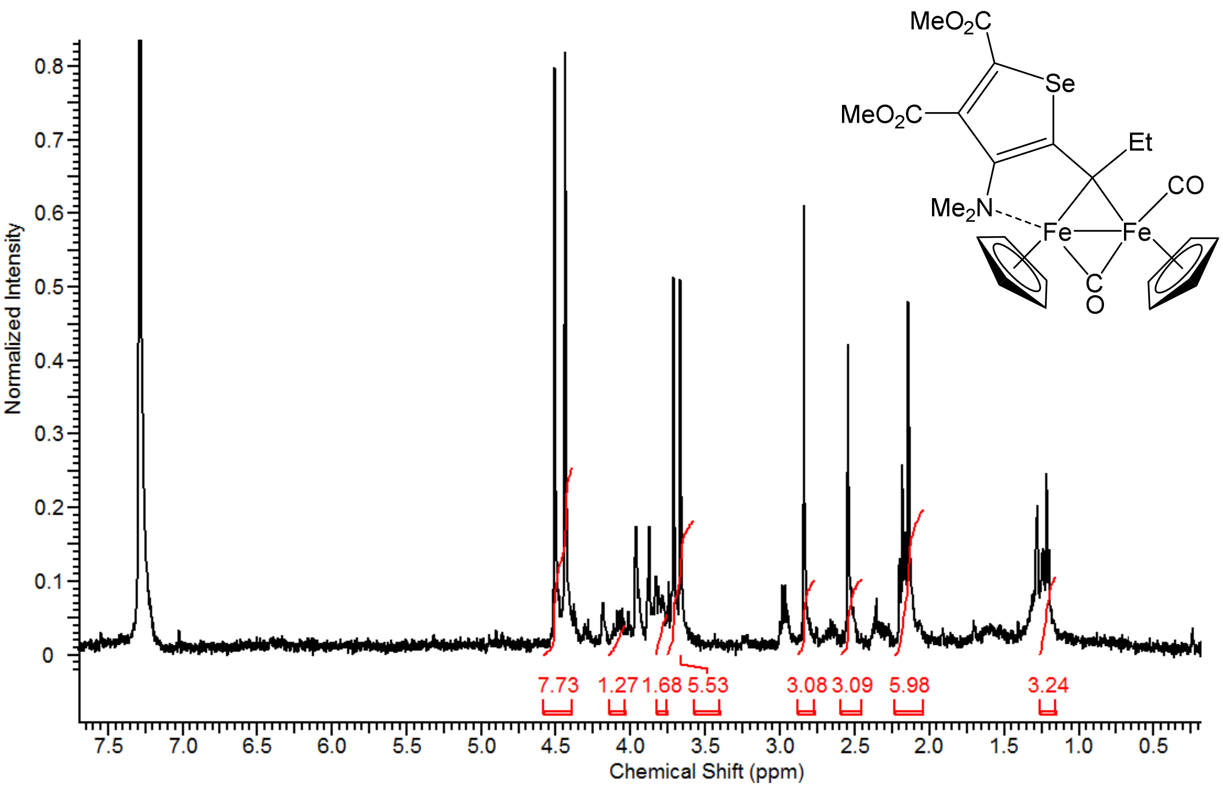

Figure S39. $^{13}C\{^{1}H\}$ NMR spectrum (101 MHz, $CDCl_3$) of **4d**.

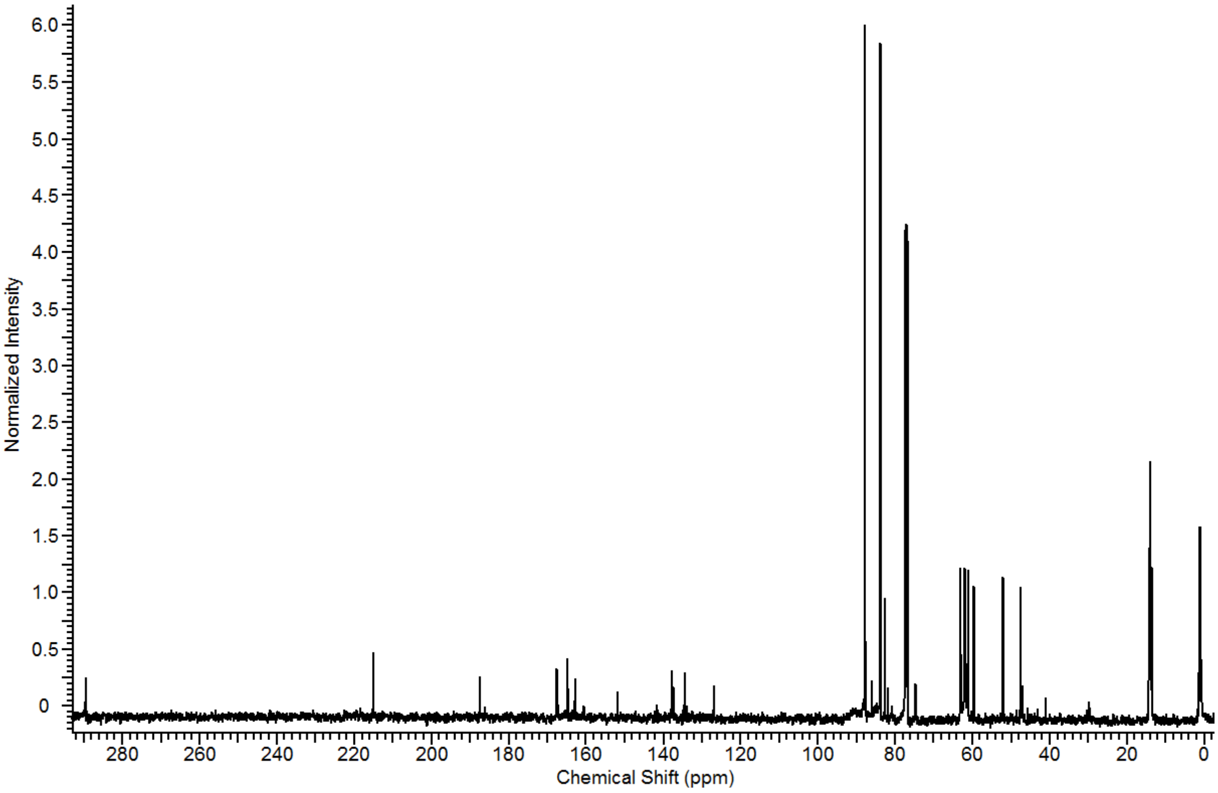

Figure S40. ^{77}Se NMR spectrum (76 MHz, $CDCl_3$) of **4d**.

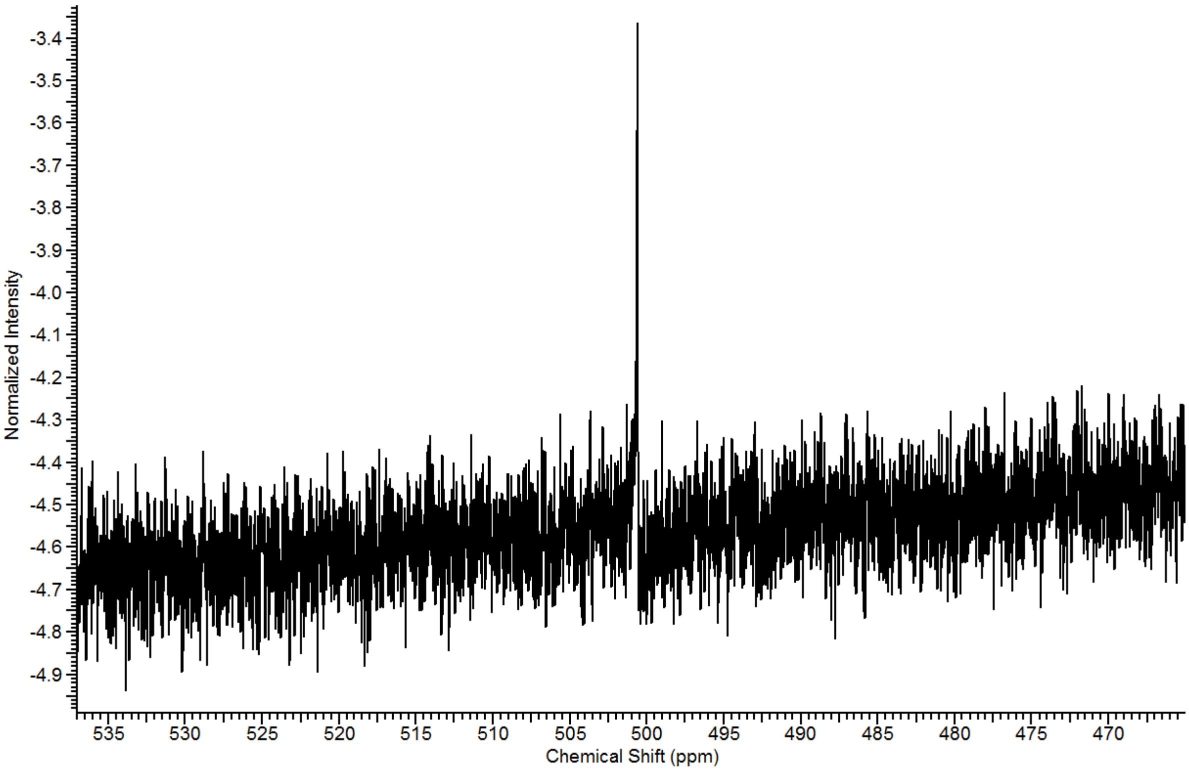

Figure S41. ^{1}H NMR spectrum (401 MHz, $CDCl_3$) of **4e**.

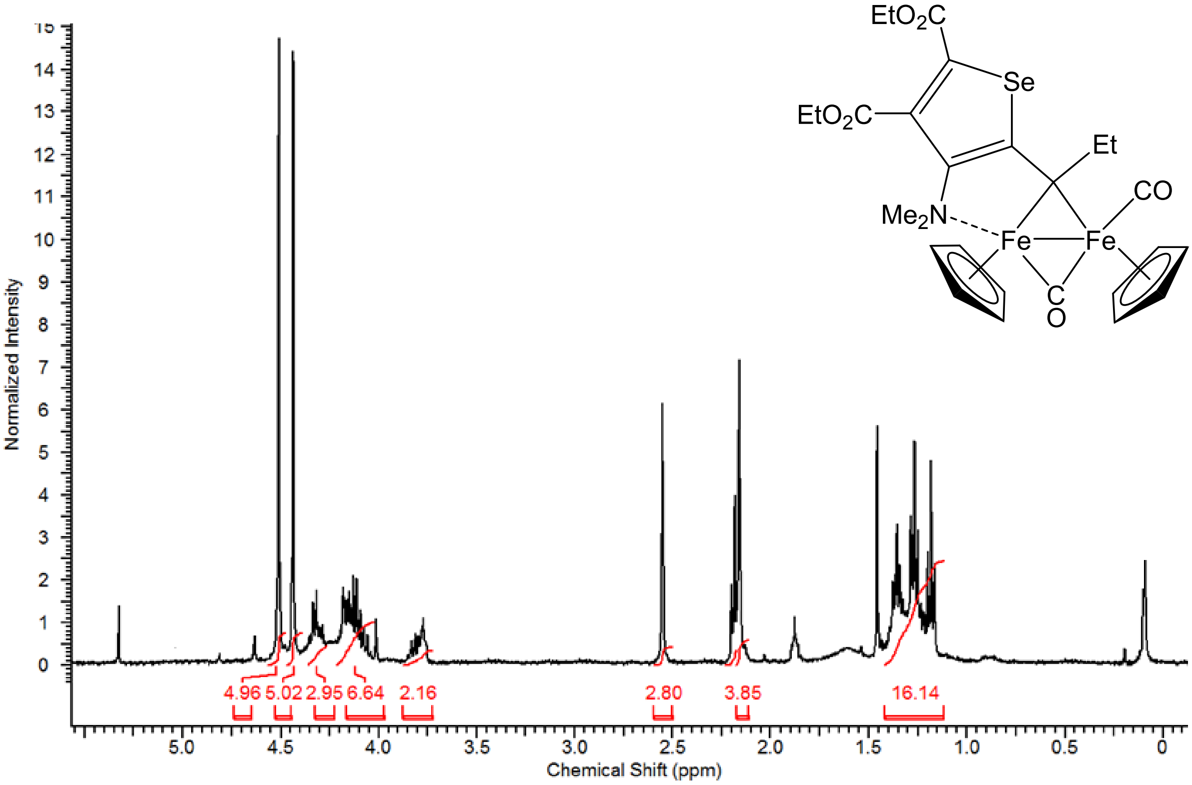

Figure S42. $^{13}C\{^{1}H\}$ NMR spectrum (101 MHz, $CDCl_3$) of **4e**.

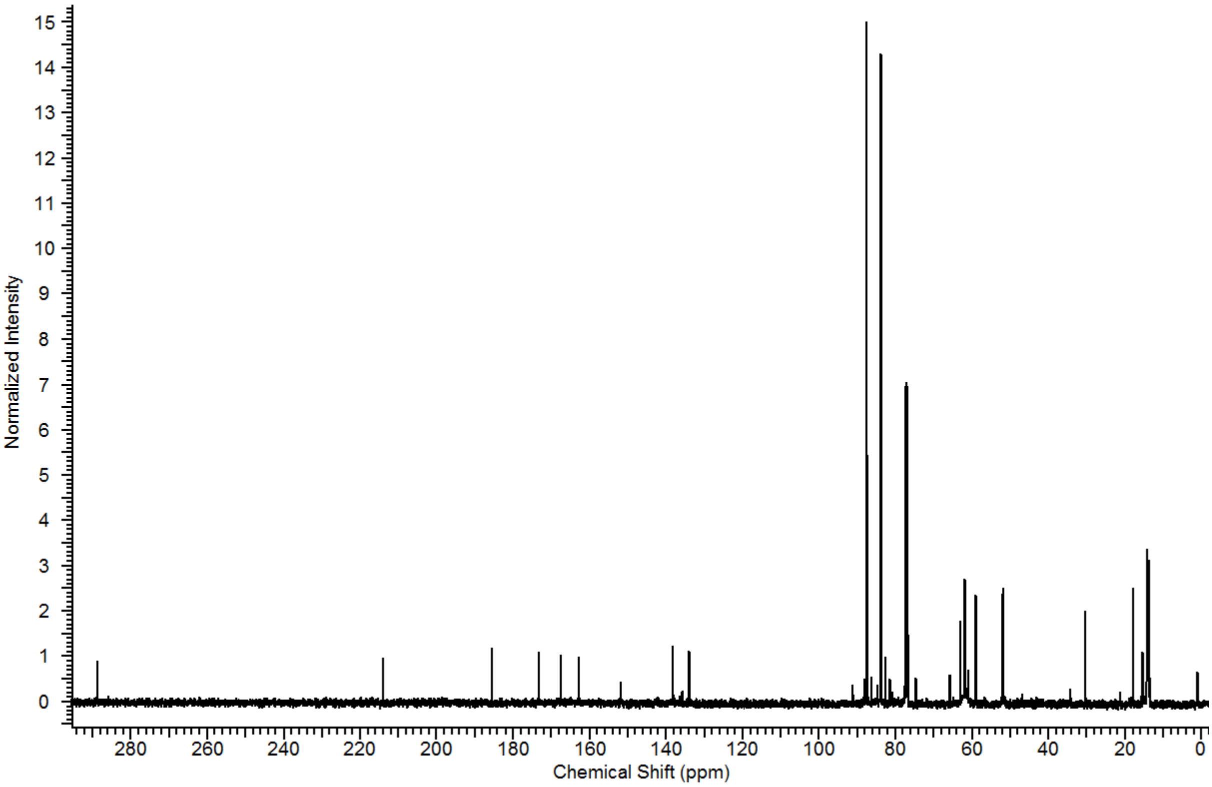

Figure S43. ^{77}Se NMR spectrum (76 MHz, $CDCl_3$) of **4e**.

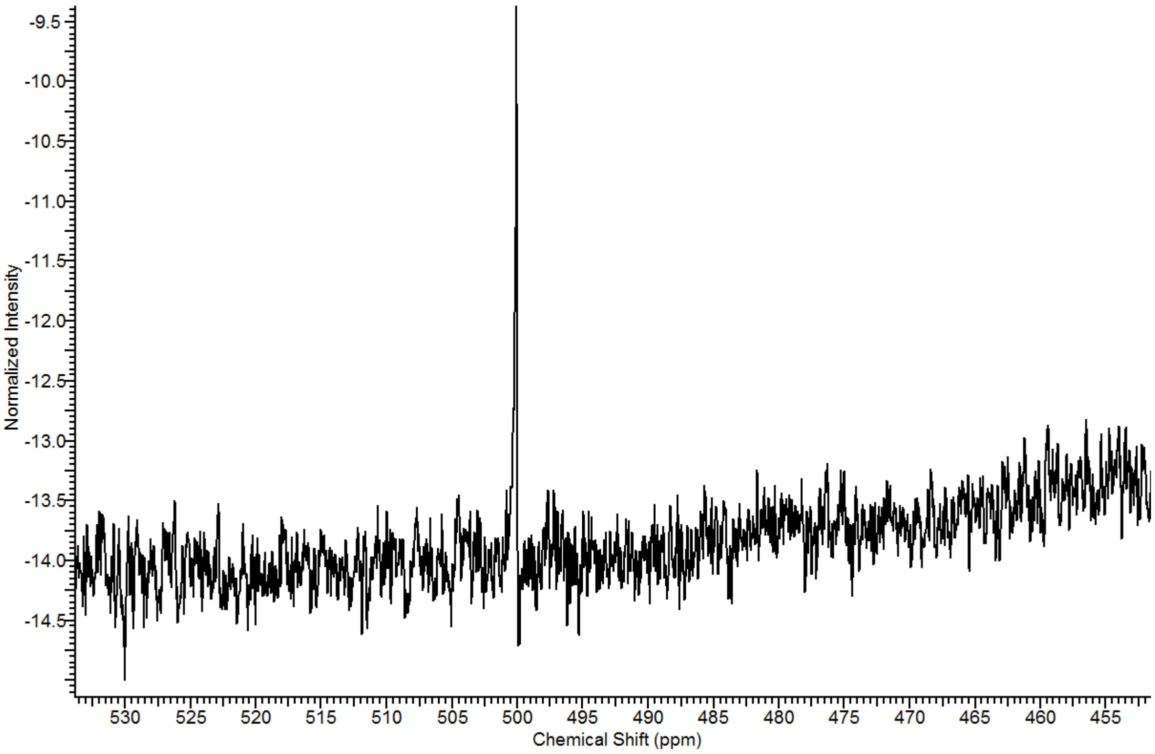

Figure S44. ^{1}H NMR spectrum (401 MHz, $CDCl_3$) of **4f**.

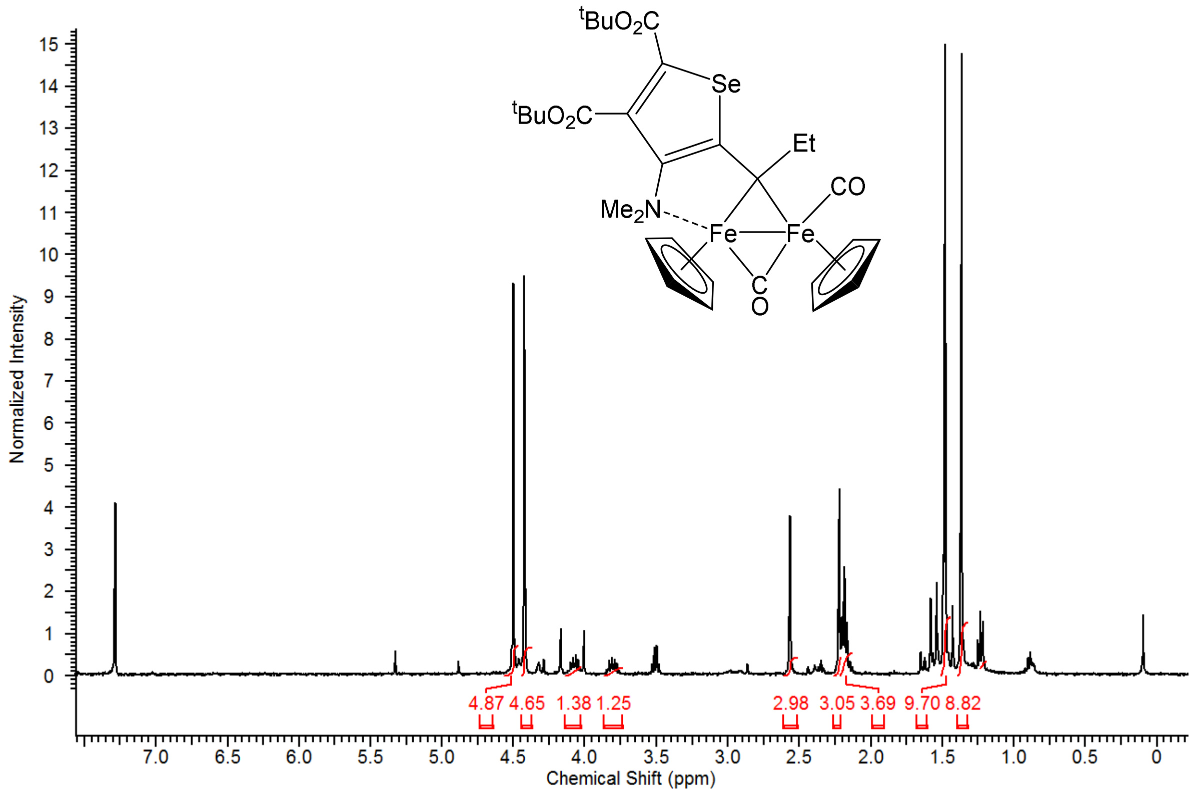

Figure S45. $^{13}C\{^{1}H\}$ NMR spectrum (101 MHz, $CDCl_3$) of **4f**.

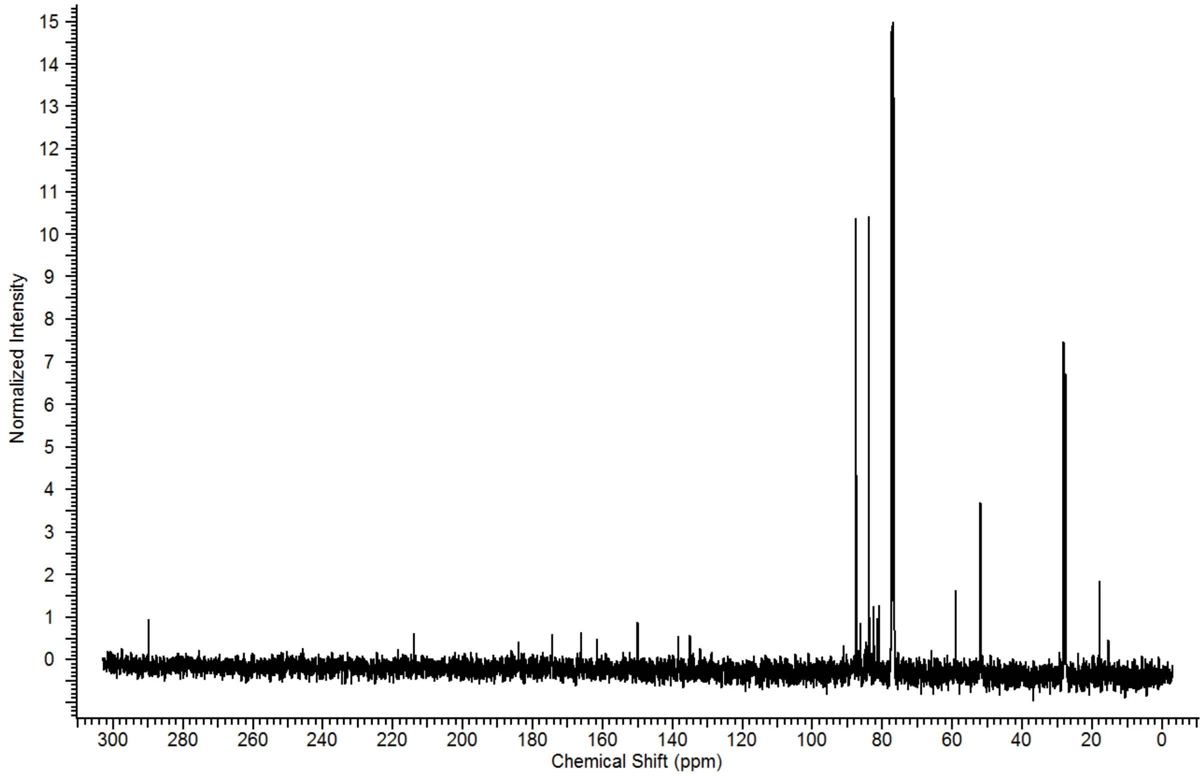

Figure S46. ^{77}Se NMR spectrum (76 MHz, $CDCl_3$) of **4f**.

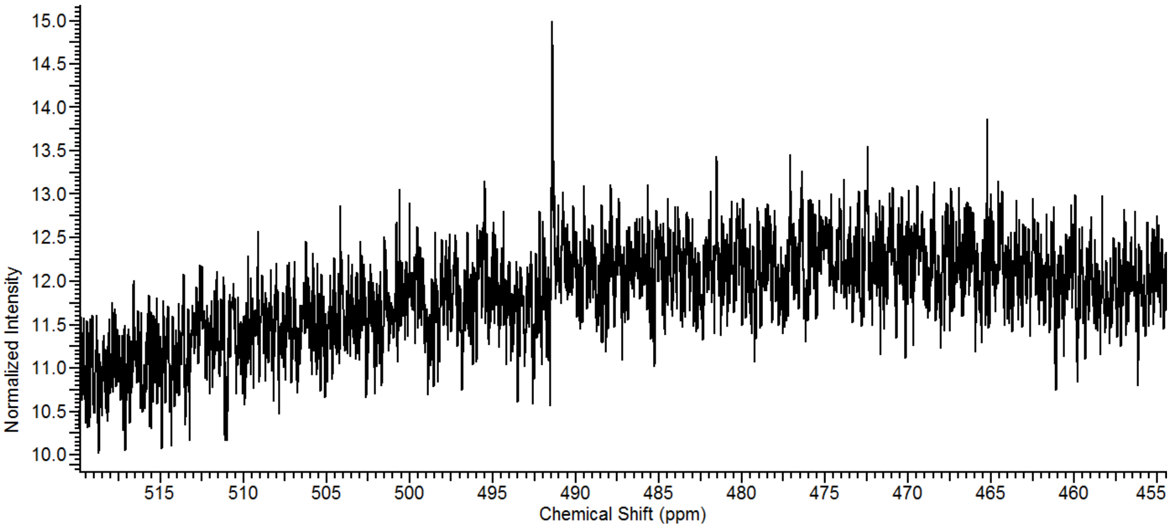

Figure S47. ^{1}H NMR spectrum (401 MHz, $CDCl_3$) of **4g**.

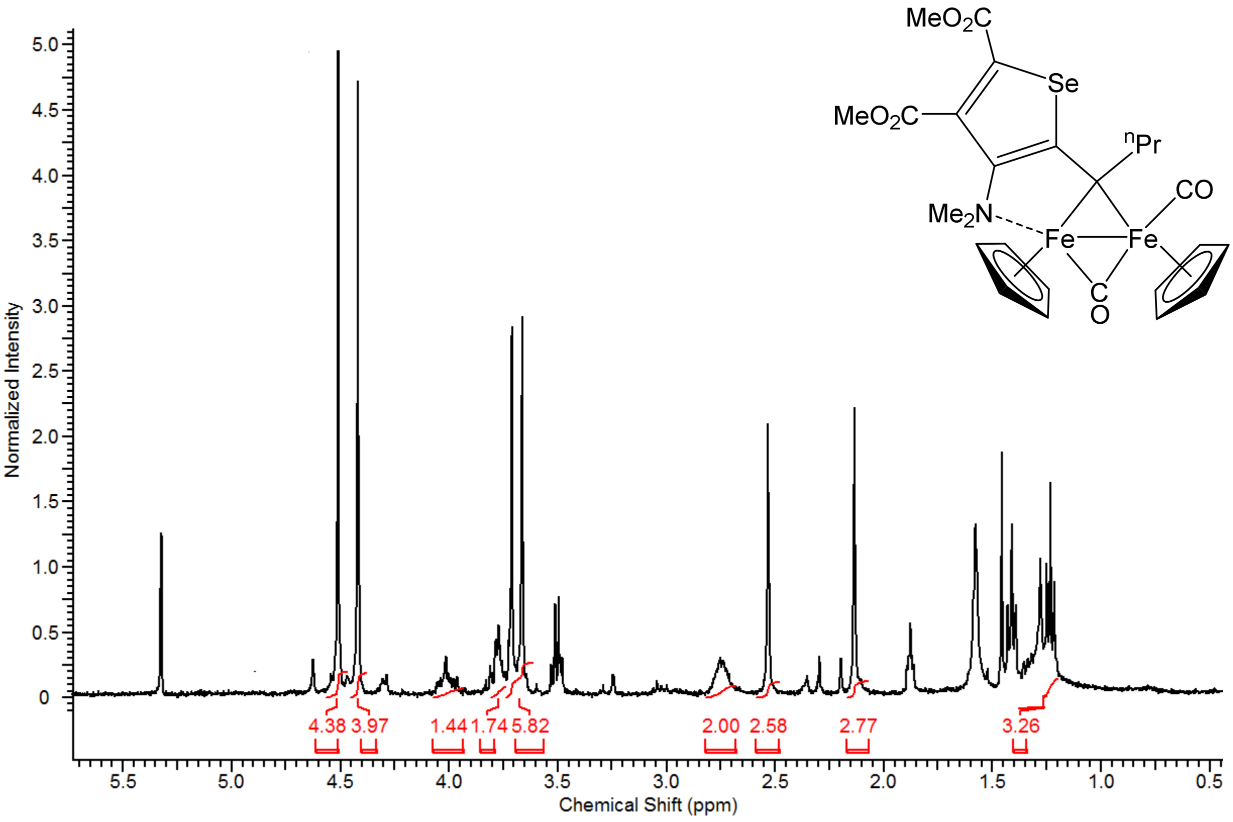

Figure S48. $^{13}C\{^{1}H\}$ NMR spectrum (101 MHz, $CDCl_3$) of **4g**.

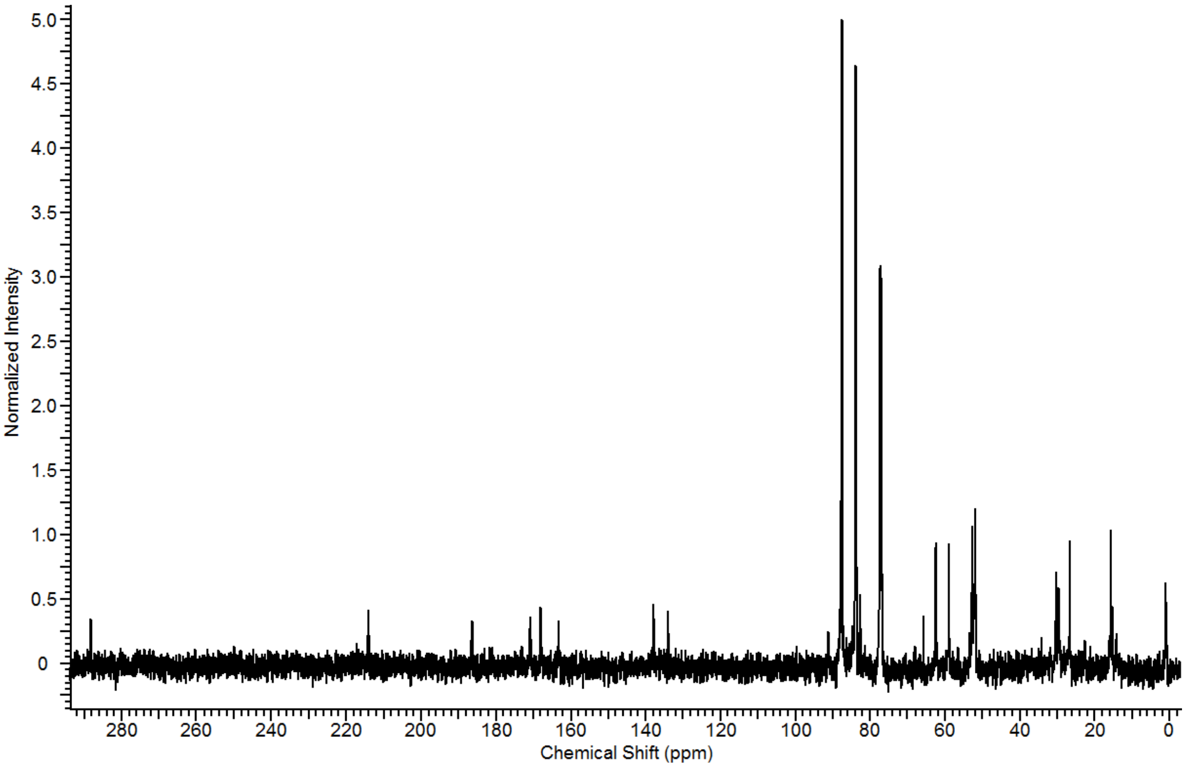

Figure S49. ^{77}Se NMR spectrum (76 MHz, $CDCl_3$) of **4g**.

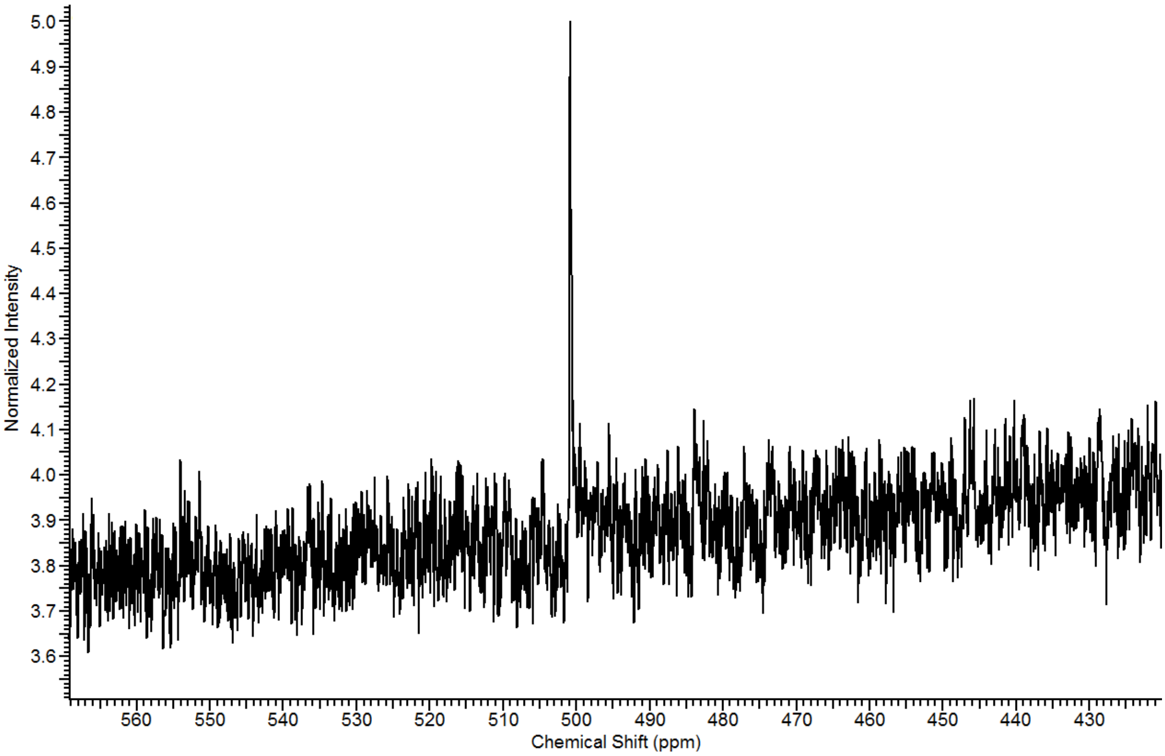

Figure S50. ^{1}H NMR spectrum (401 MHz, $CDCl_3$) of **4h**.

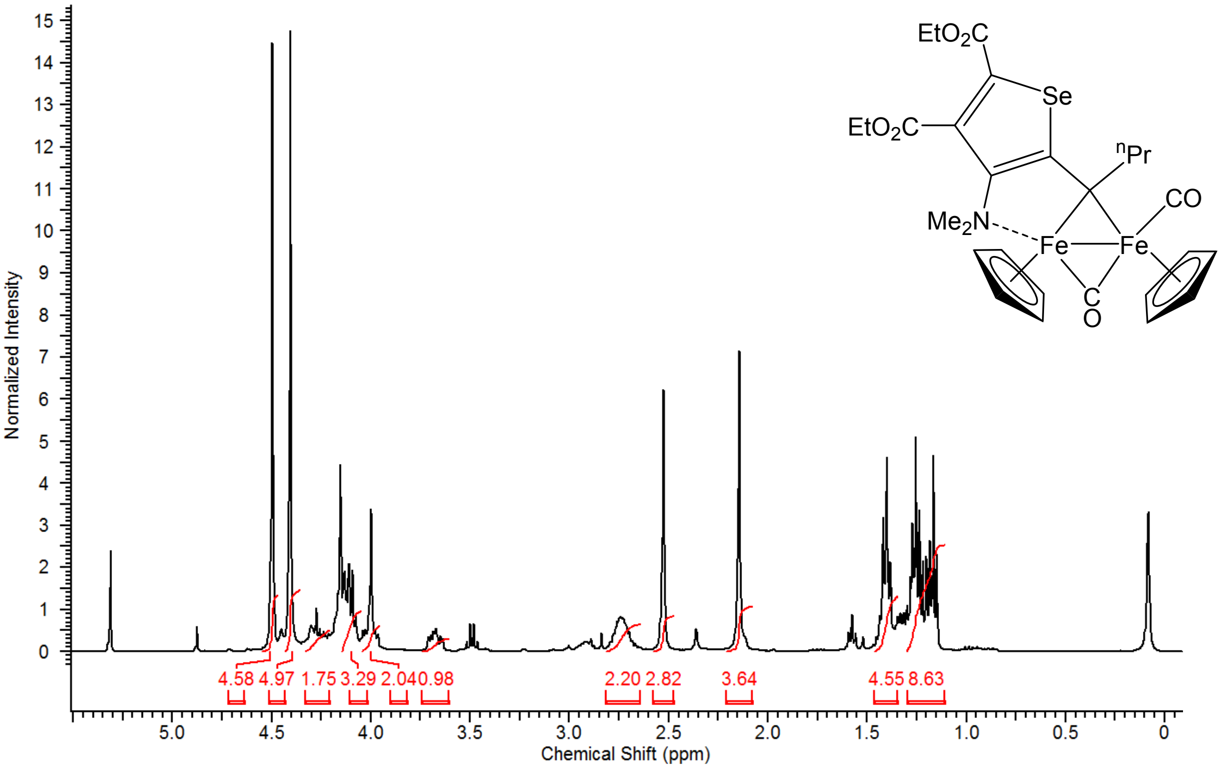

Figure S51. $^{13}C\{^{1}H\}$ NMR spectrum (101 MHz, $CDCl_3$) of **4h**.

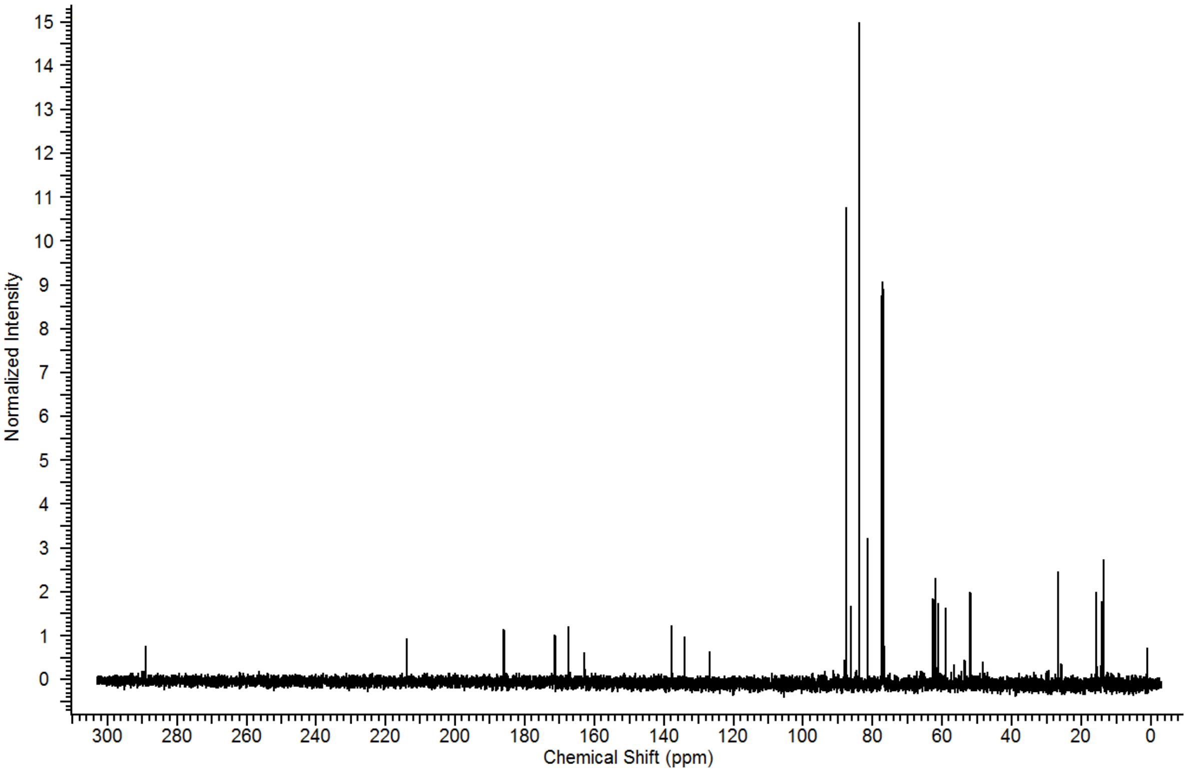

Figure S52. ^{77}Se NMR spectrum (76 MHz, $CDCl_3$) of **4h**.

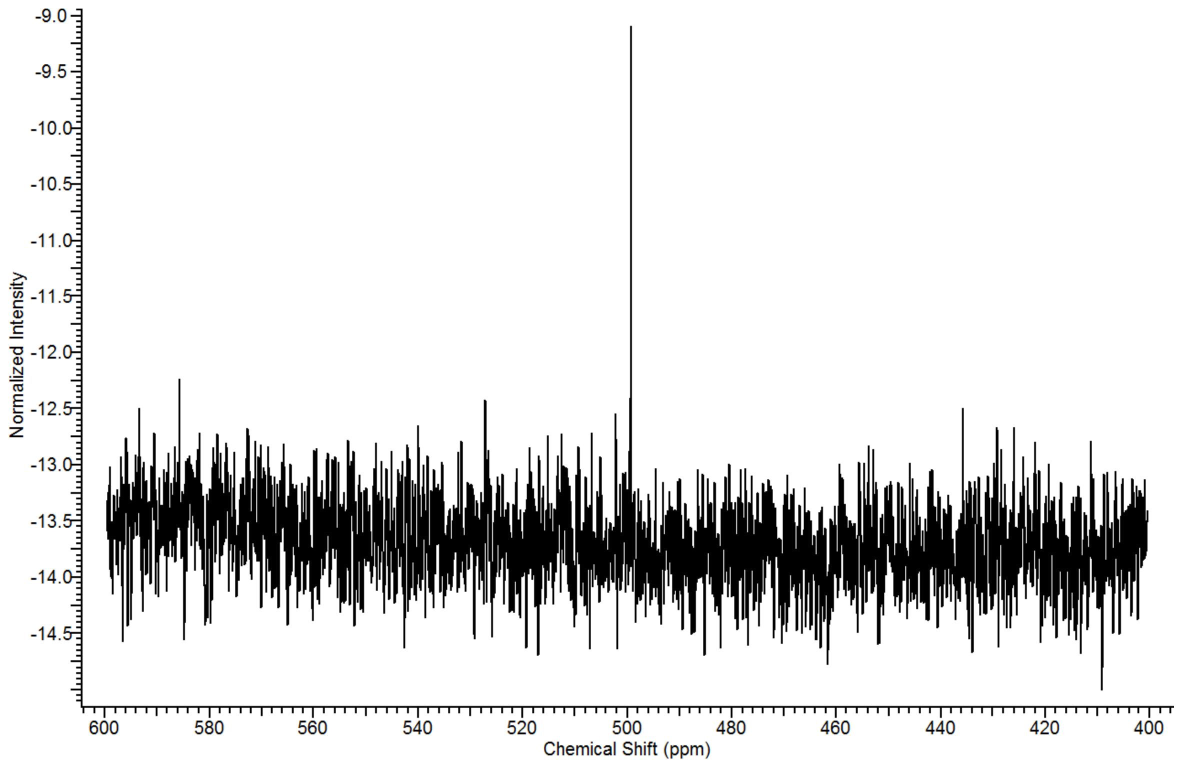

Figure S53. ^{1}H NMR spectrum (401 MHz, $CDCl_3$) of **4i**.

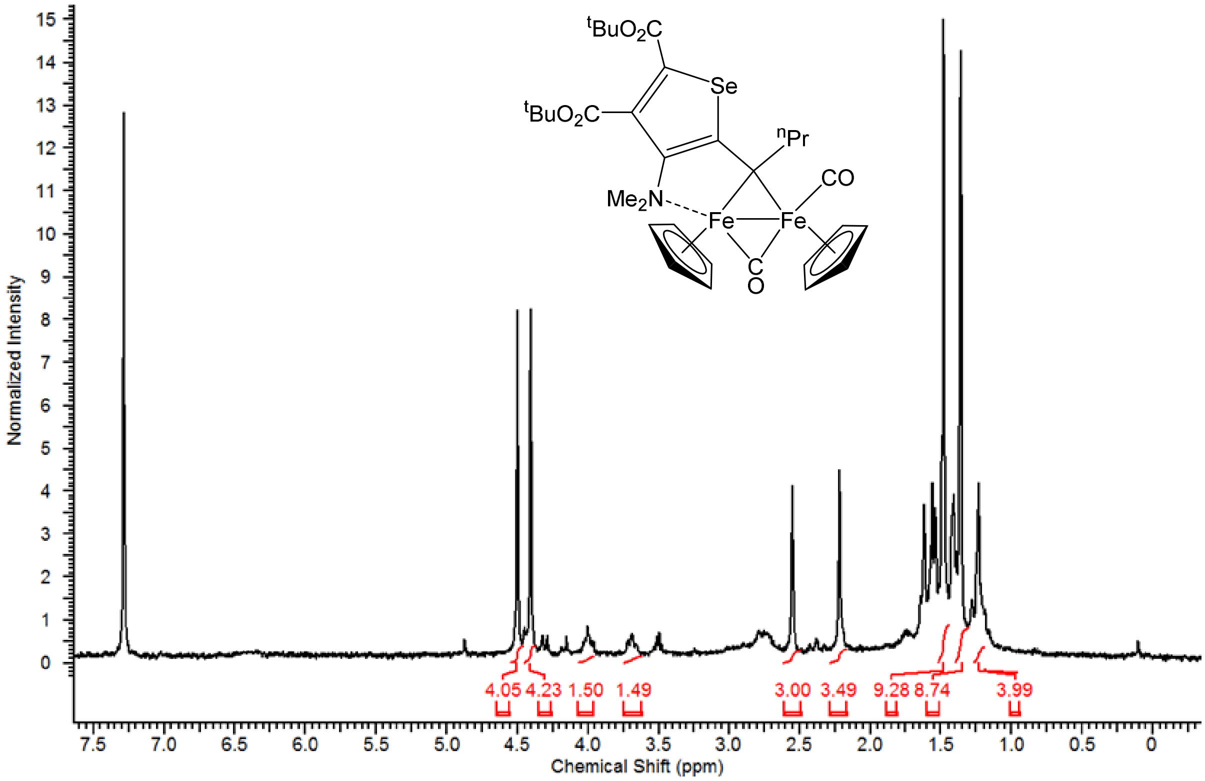

Figure S54. $^{13}C\{^{1}H\}$ NMR spectrum (101 MHz, $CDCl_3$) of **4i**.

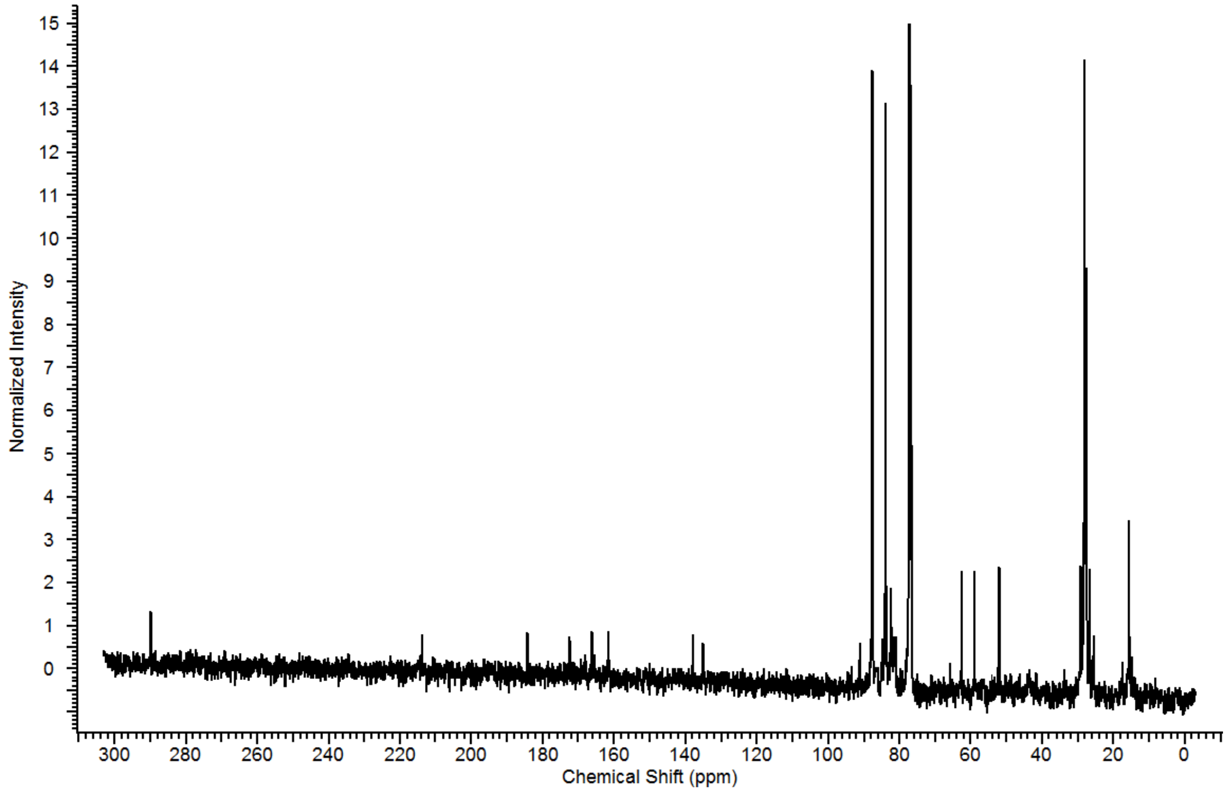

Figure S55. ^{77}Se NMR spectrum (76 MHz, $CDCl_3$) of **4i**.

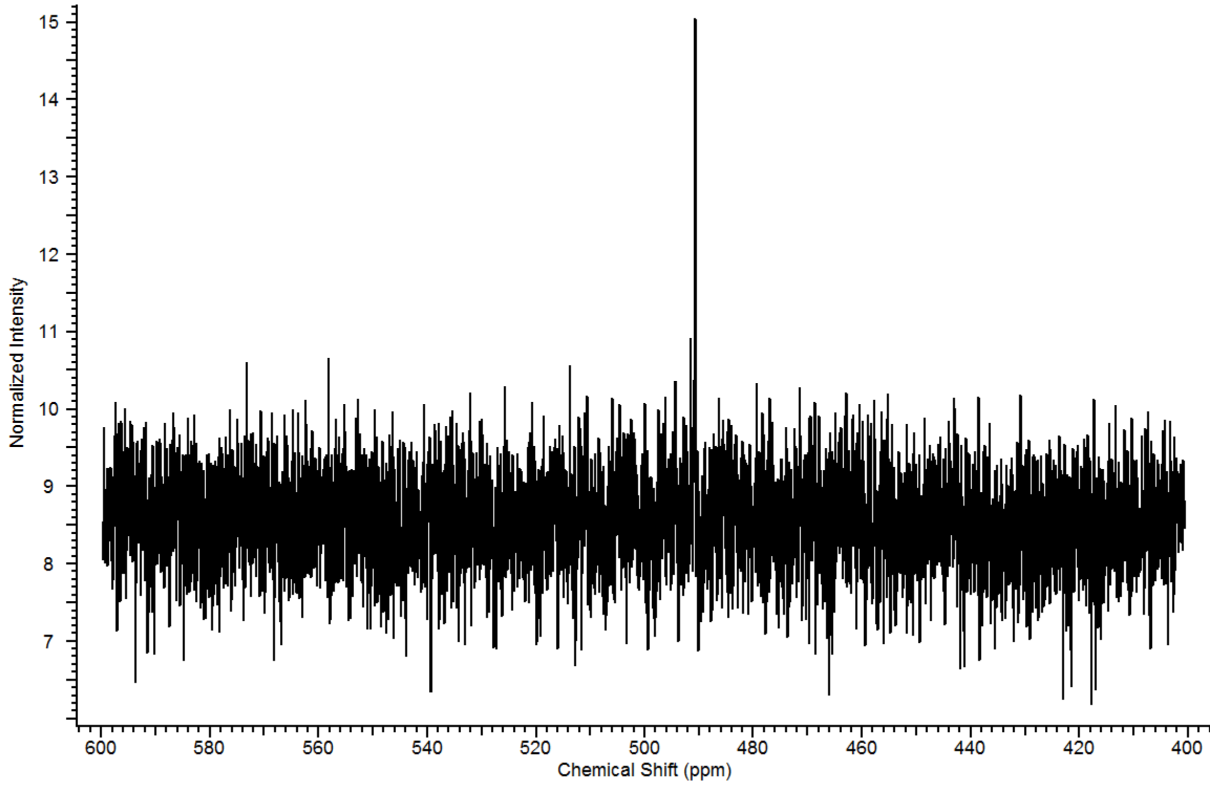

Figure S56. ^{1}H NMR spectrum (401 MHz, $CDCl_3$) of **5a**.

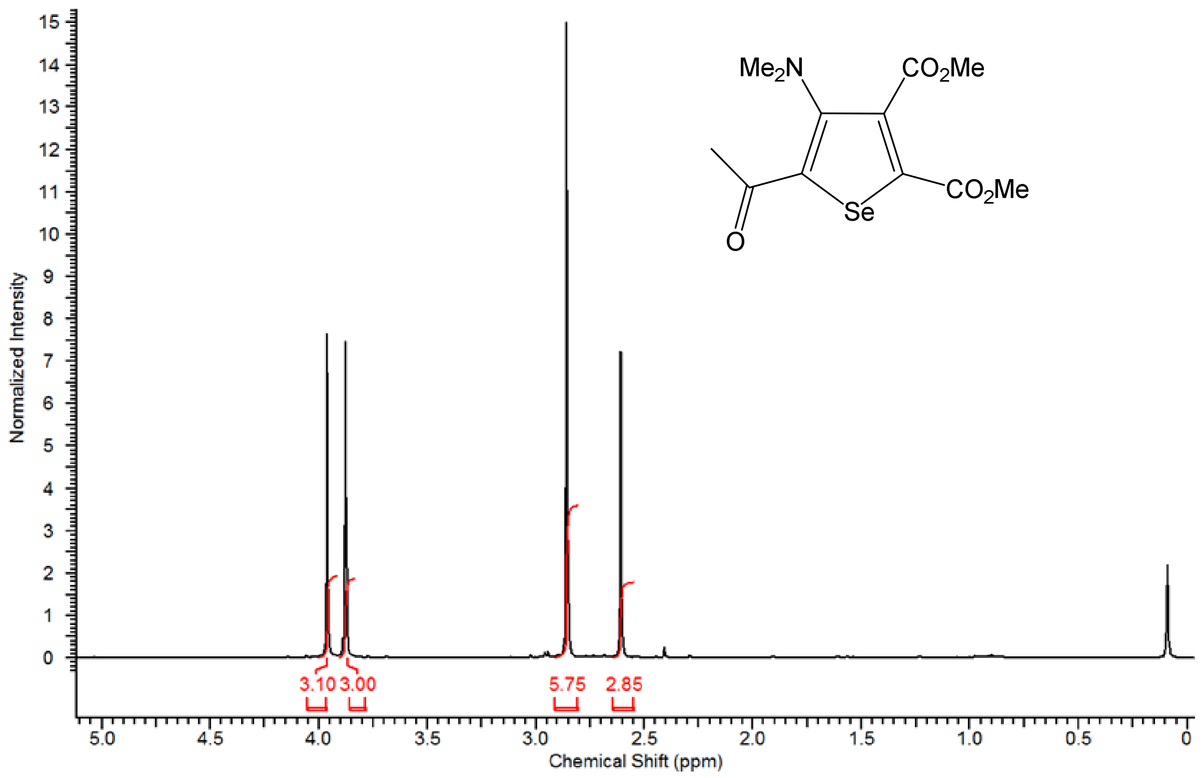

Figure S57. $^{13}C\{^{1}H\}$ NMR spectrum (101 MHz, $CDCl_3$) of **5a**.

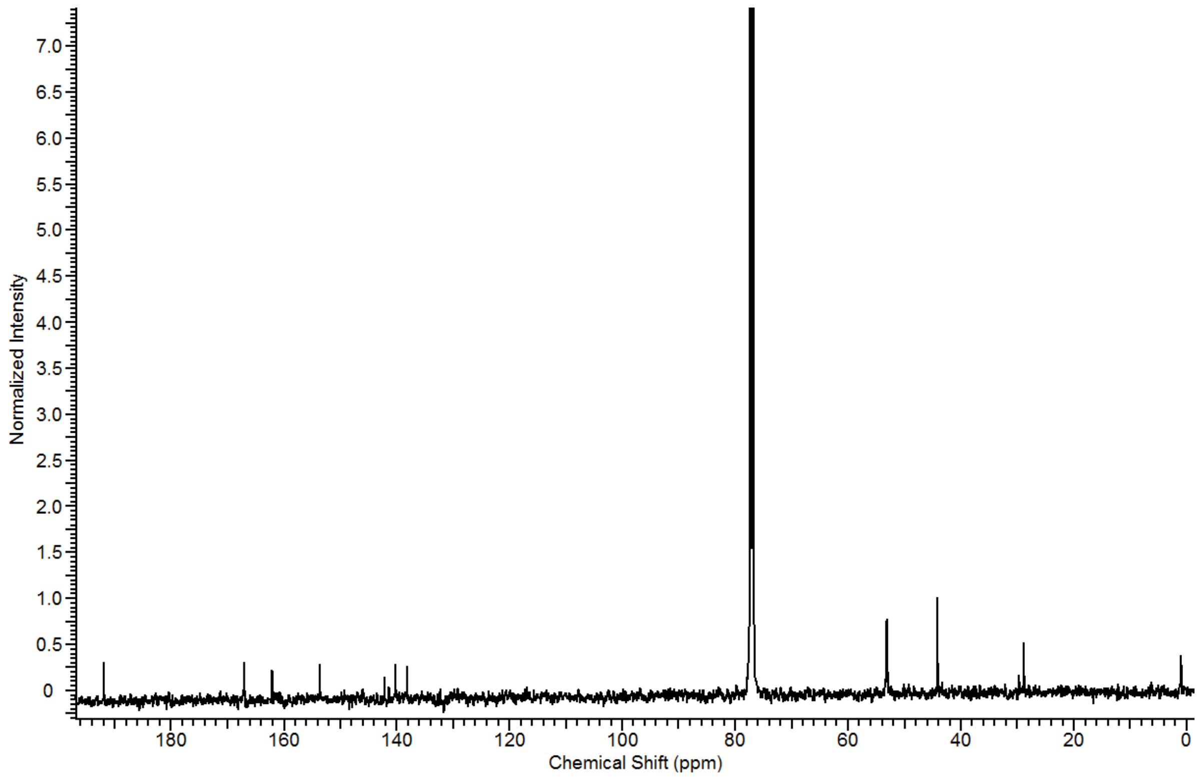

Figure S58. ^{77}Se NMR spectrum (76 MHz, $CDCl_3$) of **5a**.

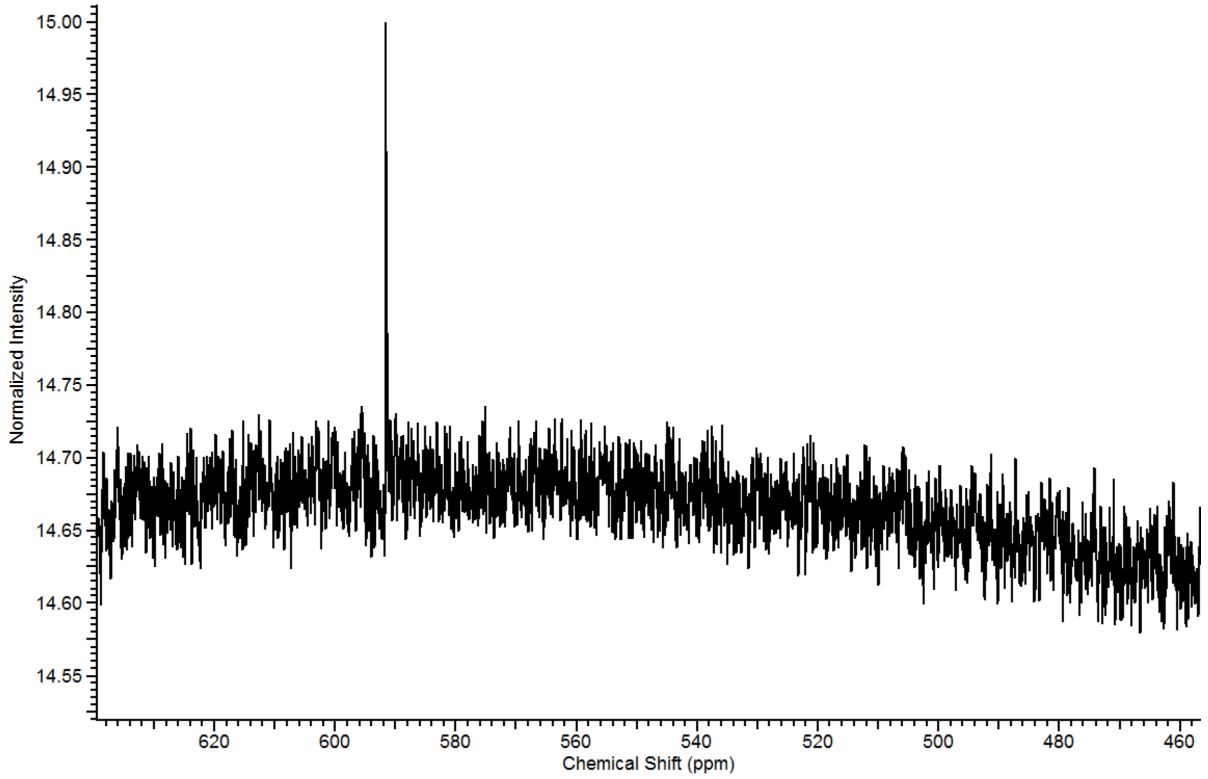

Figure S59. ^{1}H NMR spectrum (401 MHz, $CDCl_3$) of **5b**.

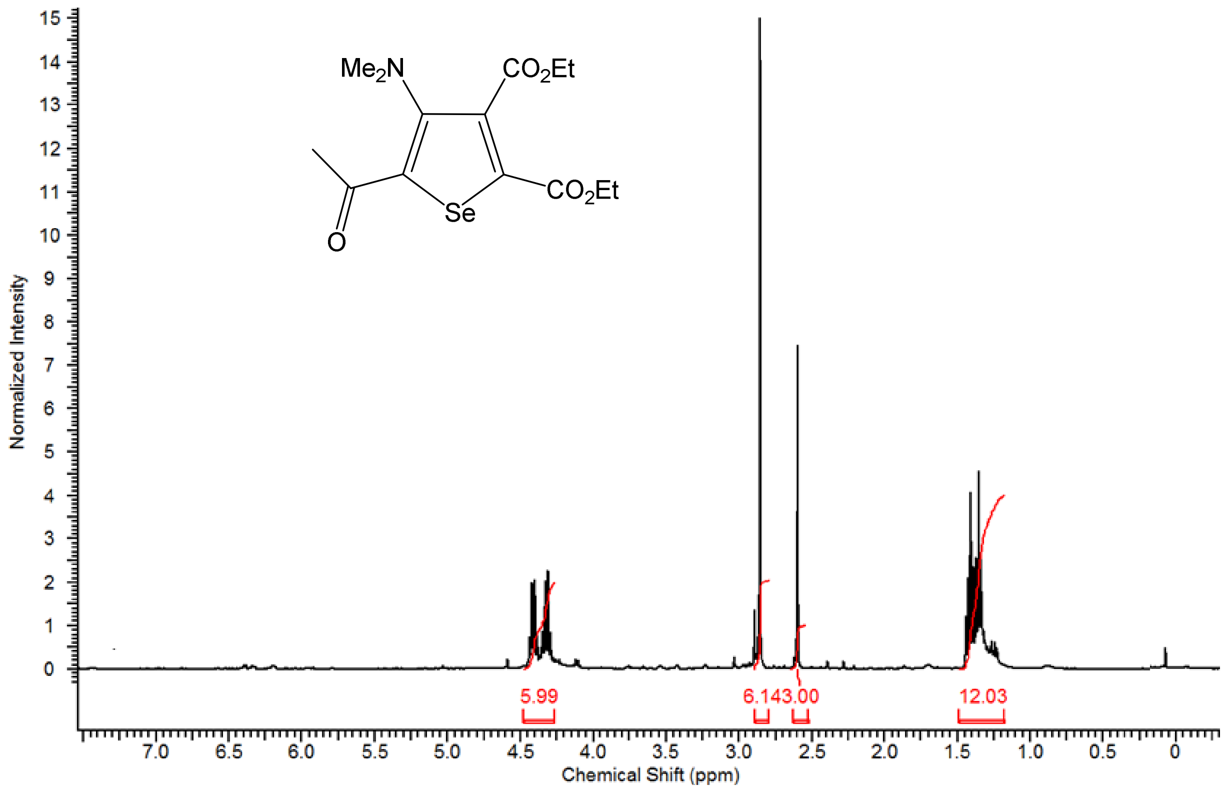

Figure S60. $^{13}C\{^{1}H\}$ NMR spectrum (101 MHz, $CDCl_3$) of **5b**.

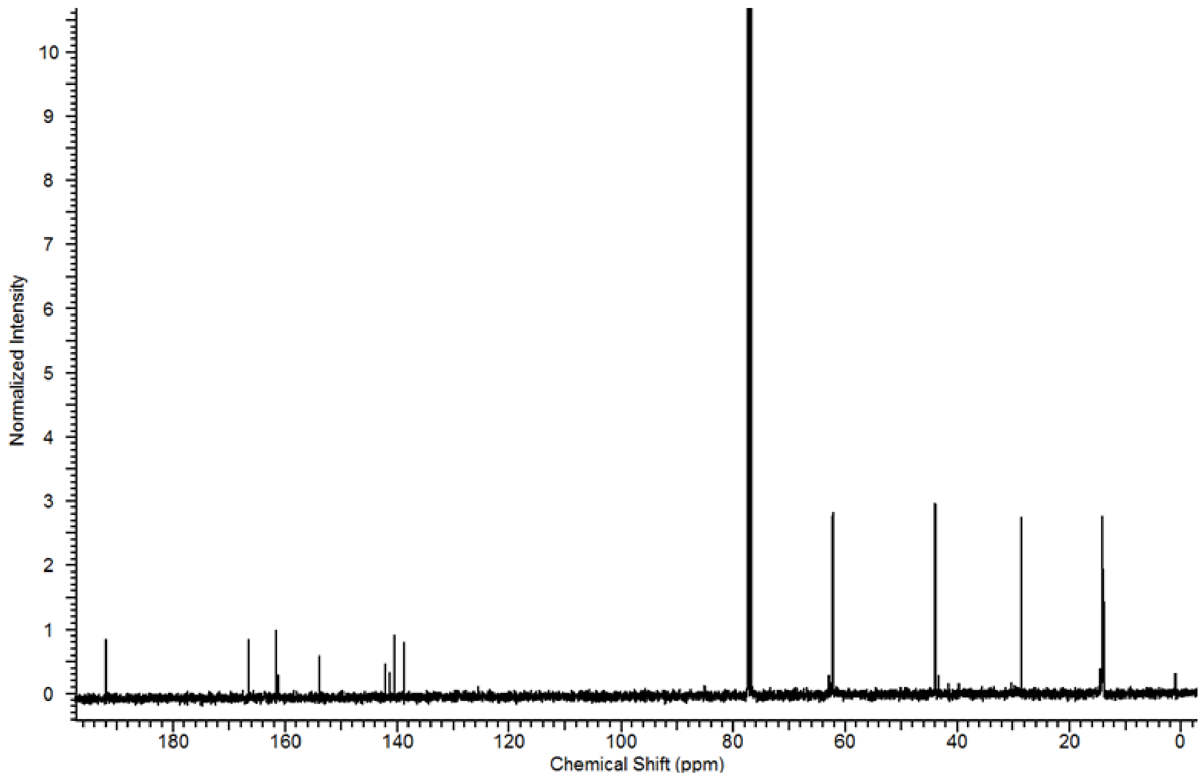

Figure S61. ^{77}Se NMR spectrum (76 MHz, $CDCl_3$) of **5b**.

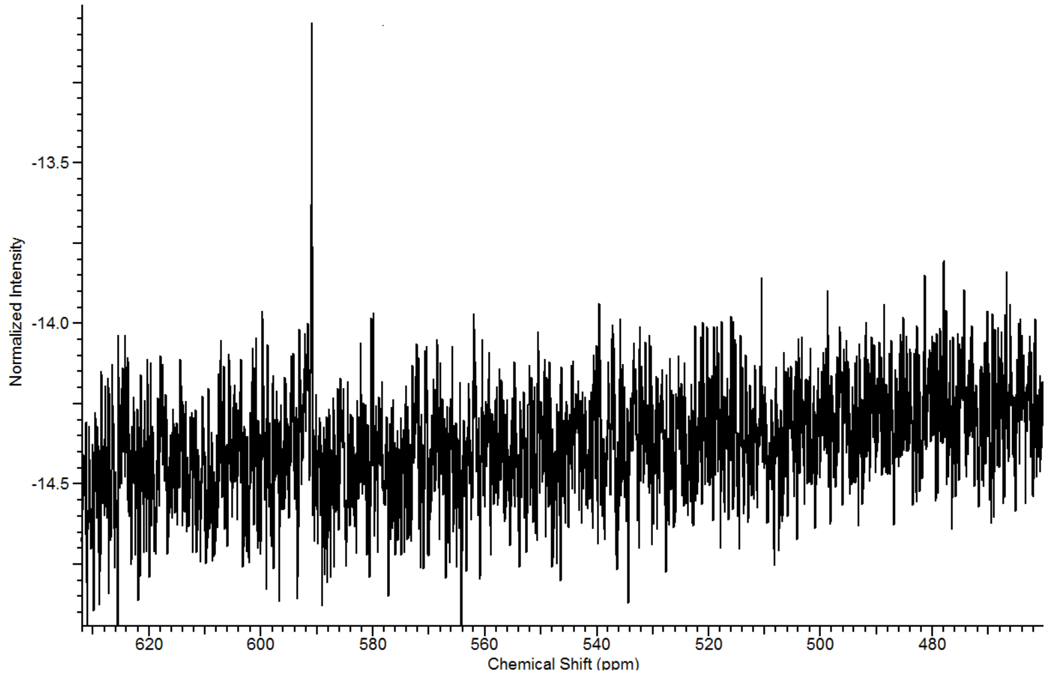

Figure S62. ^{1}H NMR spectrum (401 MHz, $CDCl_3$) of **5c**.

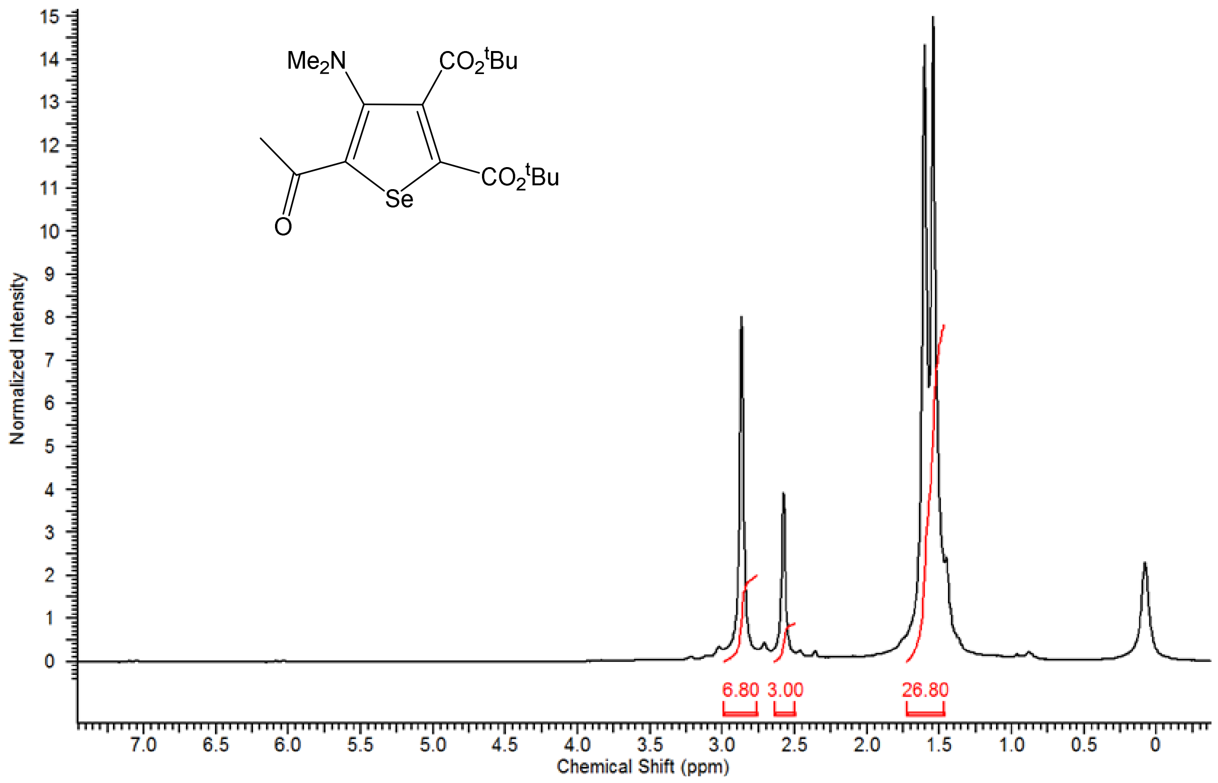

Figure S63. $^{13}C\{^{1}H\}$ NMR spectrum (101 MHz, $CDCl_3$) of **5c**.

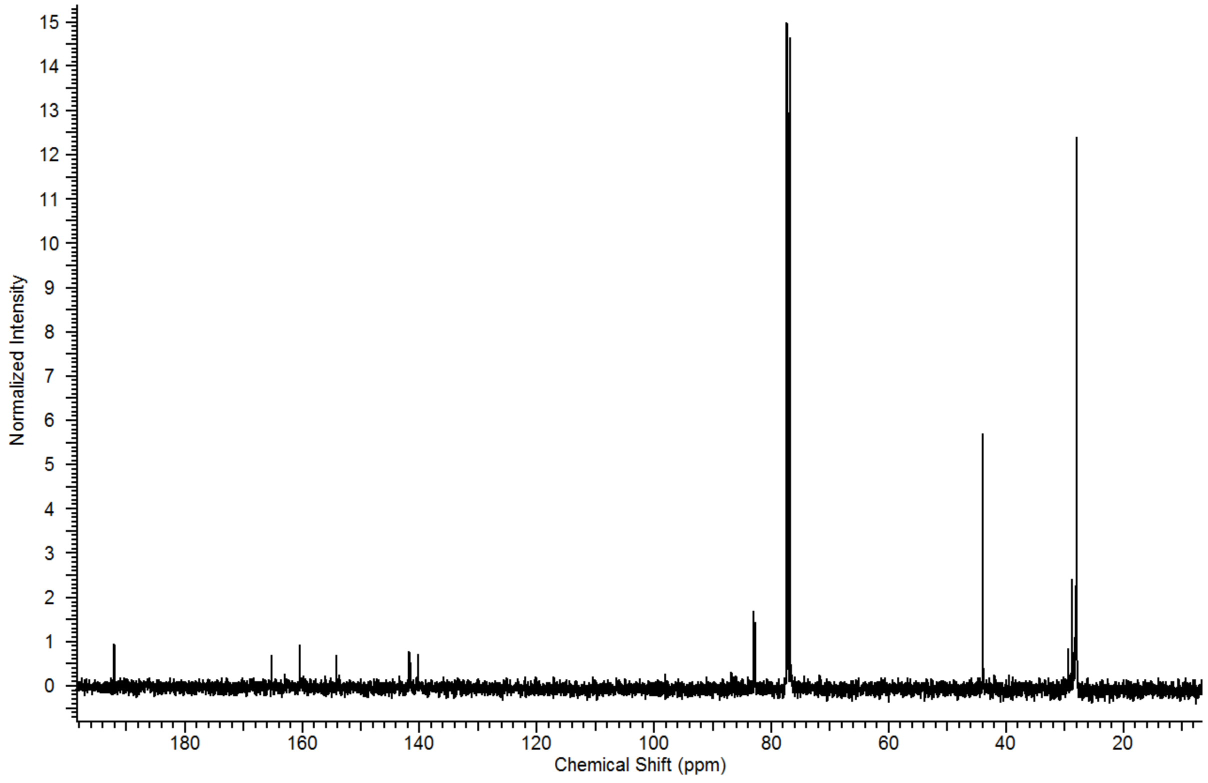

Figure S64. ^{77}Se NMR spectrum (76 MHz, $CDCl_3$) of **5c**.

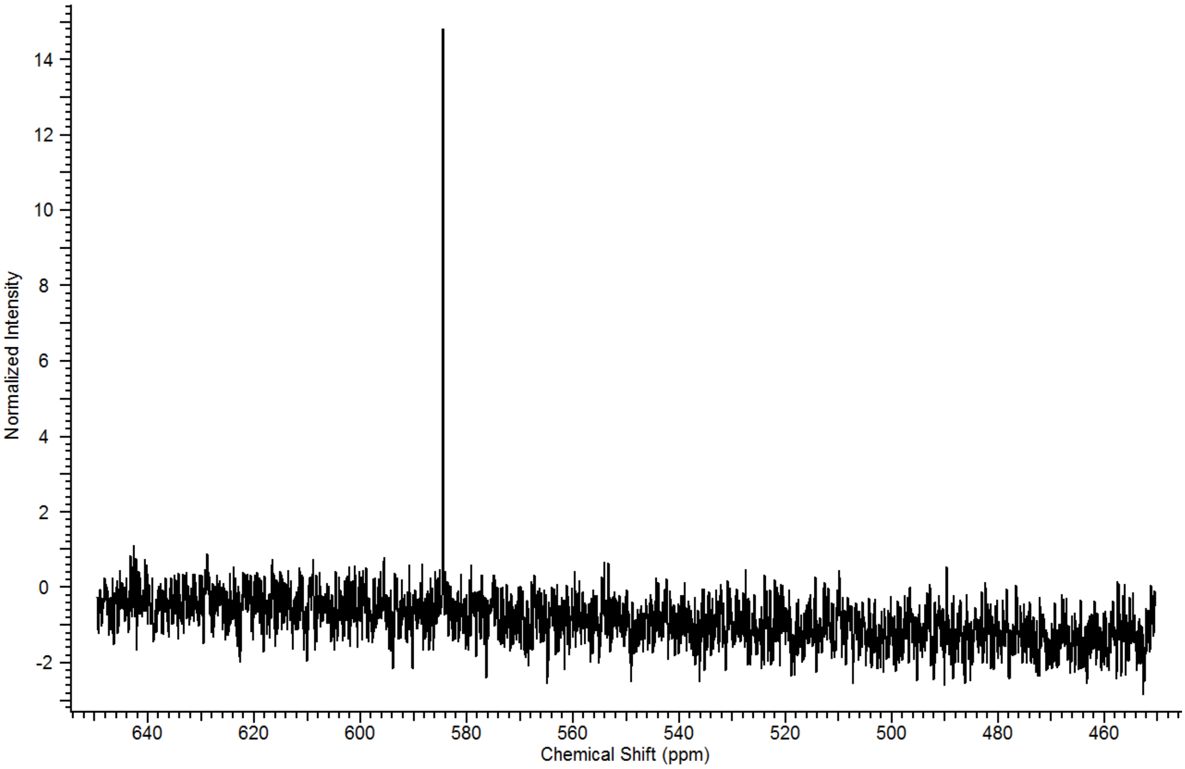

Figure S65. ^{1}H NMR spectrum (401 MHz, $CDCl_3$) of **5d**.

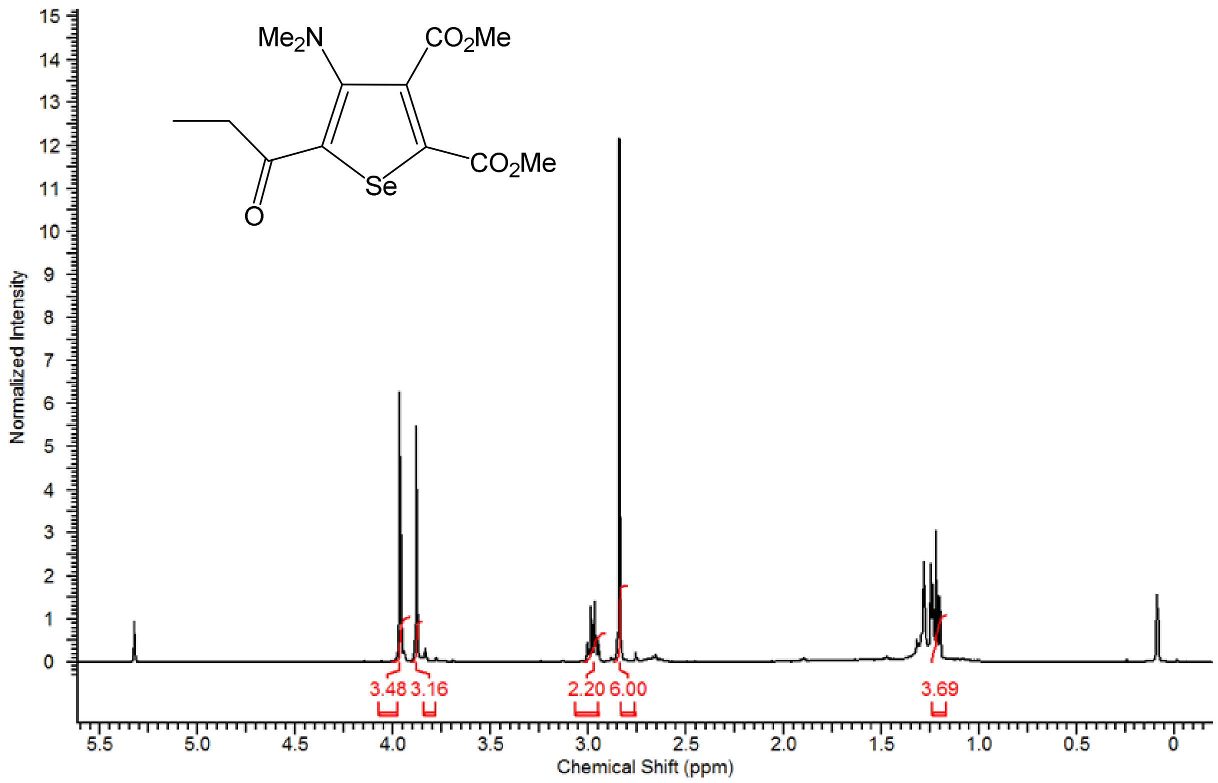

Figure S66. $^{13}C\{^{1}H\}$ NMR spectrum (101 MHz, $CDCl_3$) of **5d**.

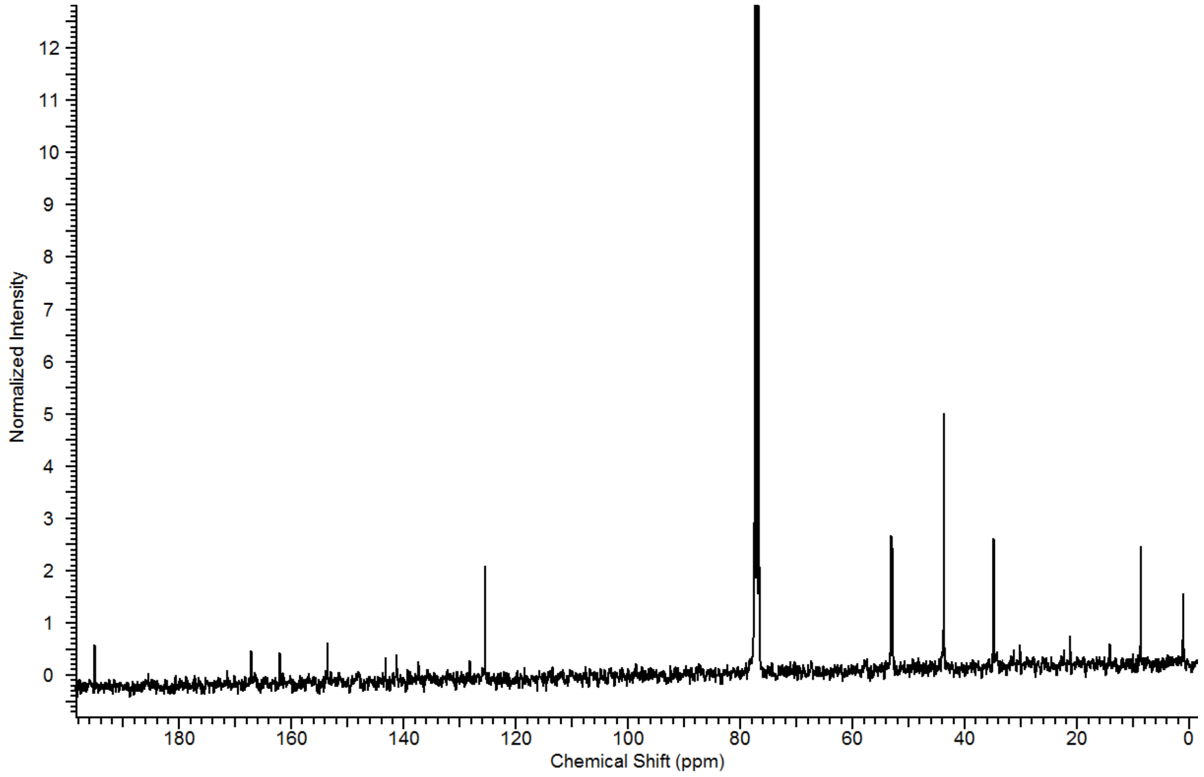

Figure S67. ^{77}Se NMR spectrum (76 MHz, $CDCl_3$) of **5d**.

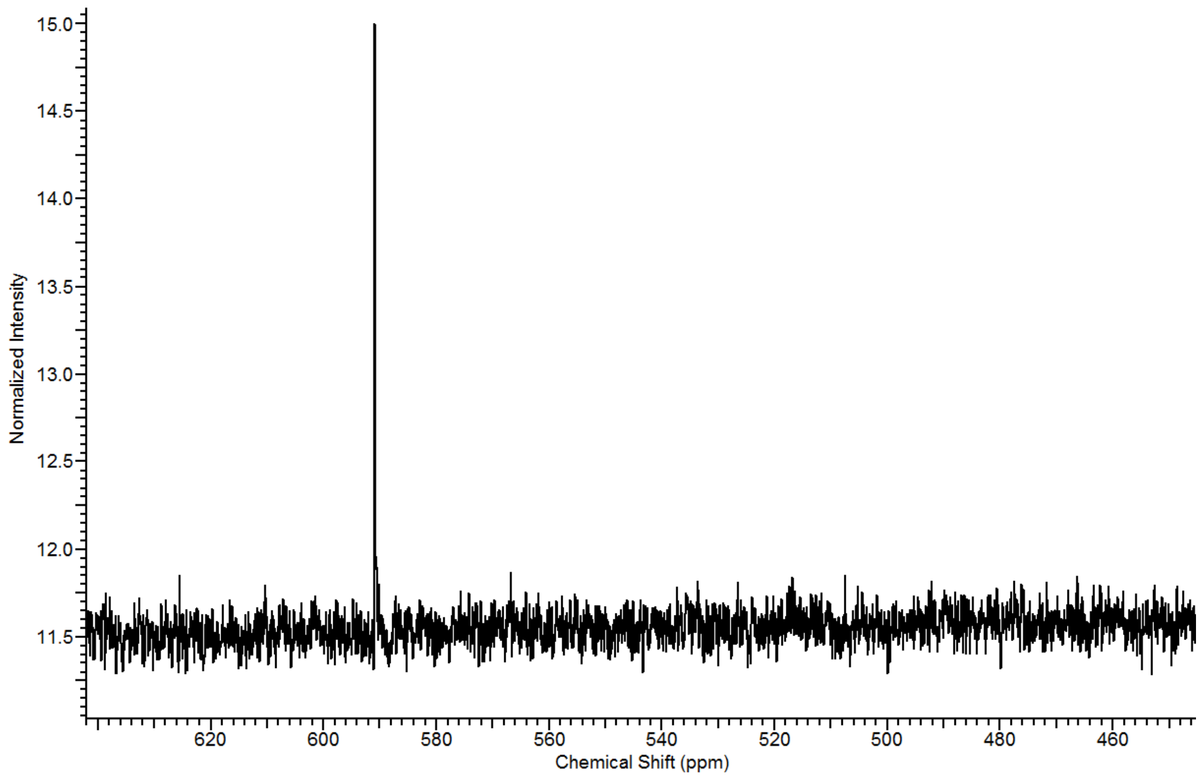

Figure S68. ^{1}H NMR spectrum (401 MHz, CDCl3) of **5e**.

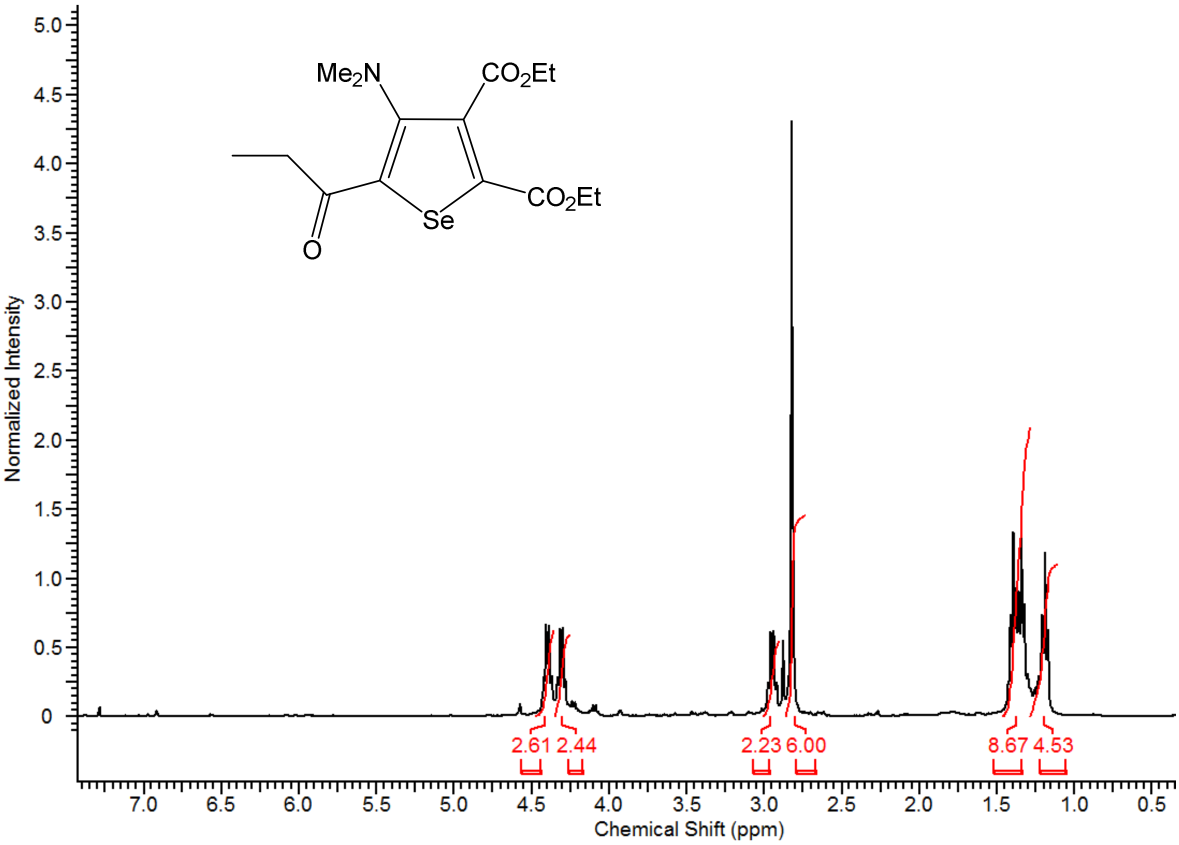

Figure S69. ^{13}C{^{1}H} NMR spectrum (101 MHz, CDCl3) of **5e**.

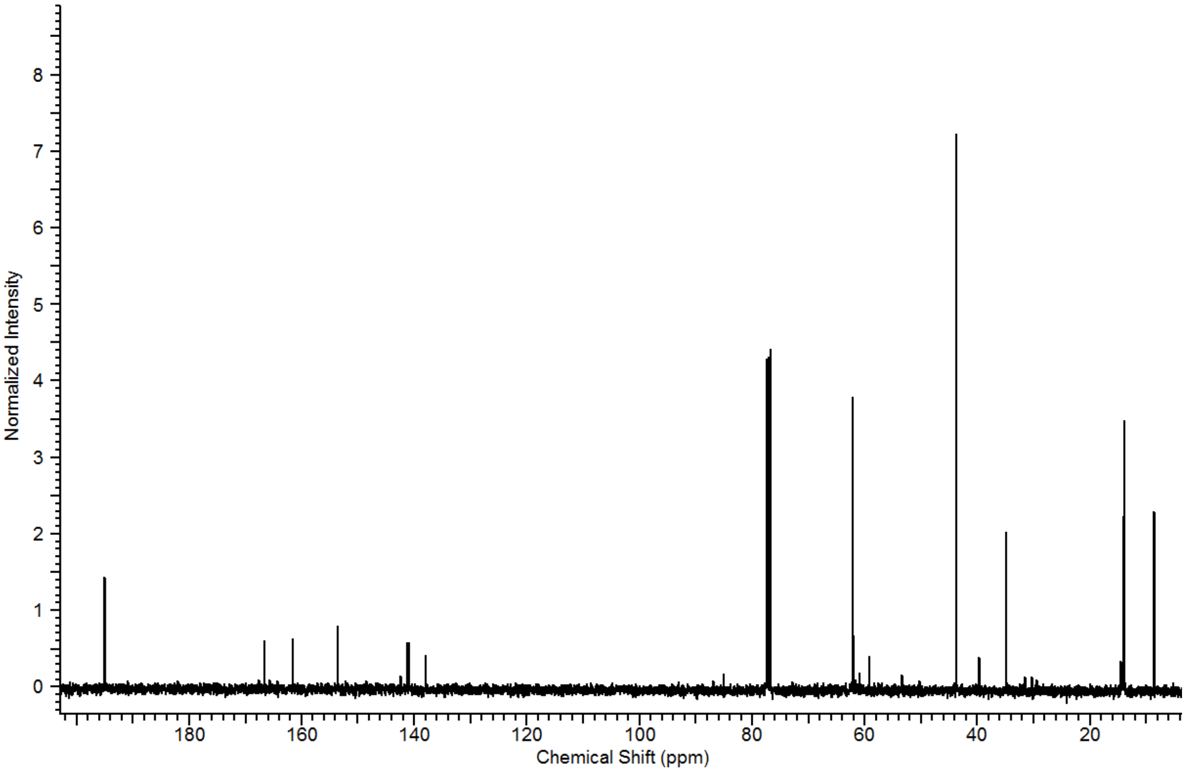

Figure S70. ^{77}Se NMR spectrum (76 MHz, $CDCl_3$) of **5e**.

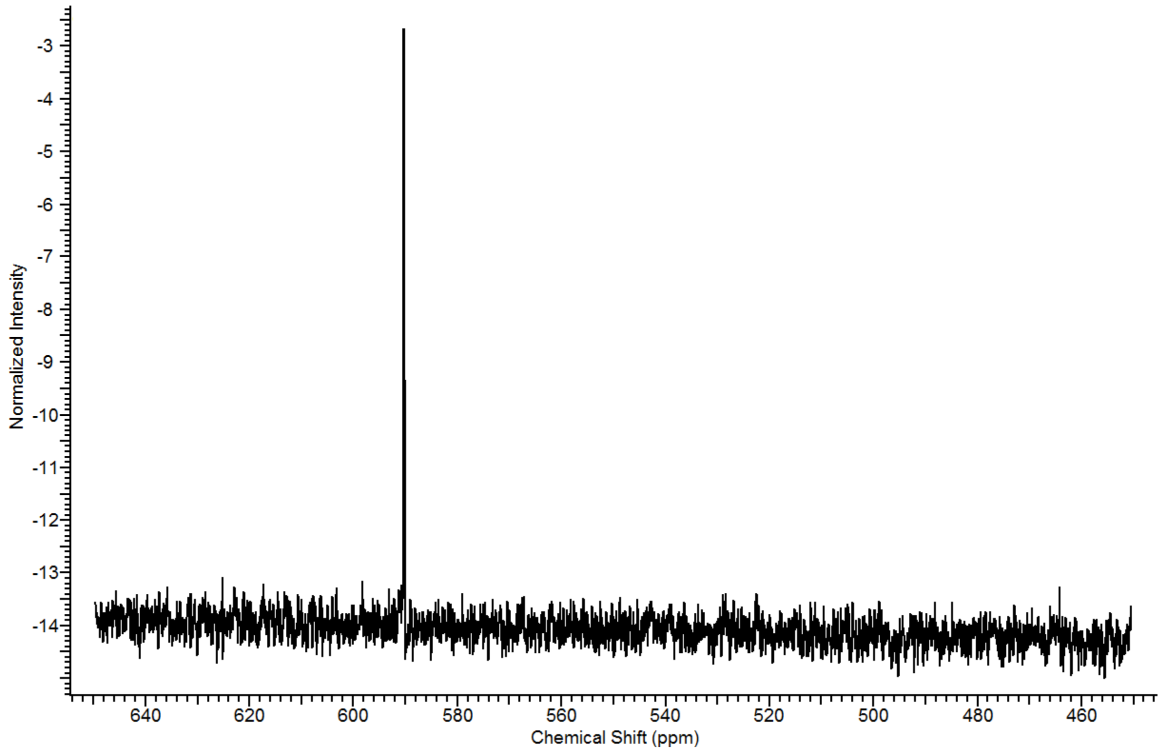

Figure S71. ^{1}H NMR spectrum (401 MHz, $CDCl_3$) of **5f**.

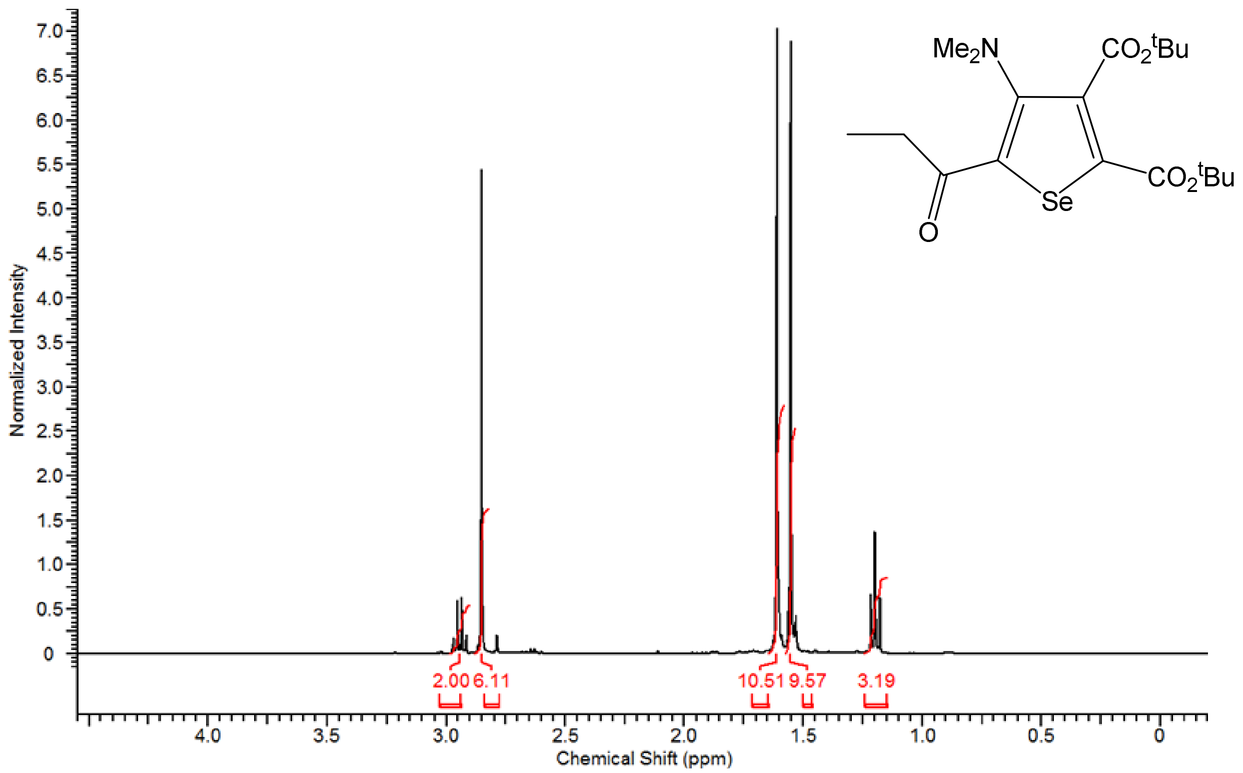

Figure S72. $^{13}C\{^{1}H\}$ NMR spectrum (101 MHz, $CDCl_3$) of **5f**.

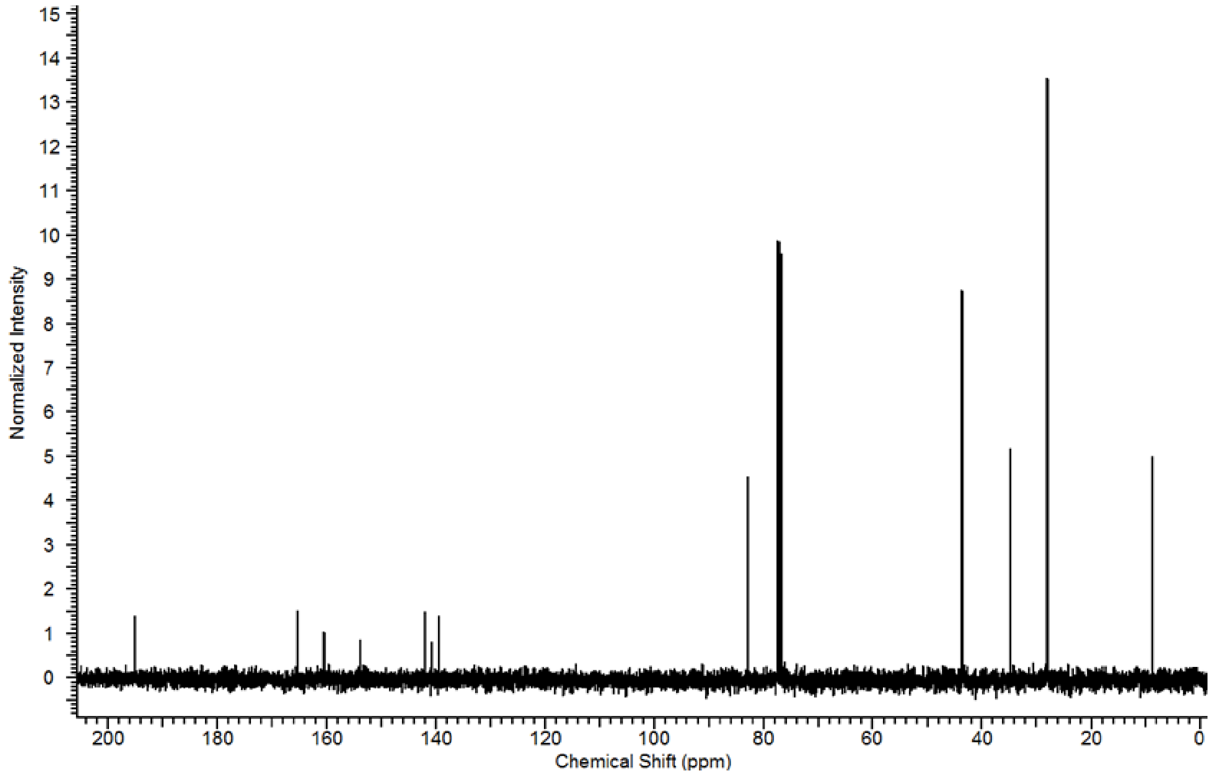

Figure S73. ^{77}Se NMR spectrum (76 MHz, $CDCl_3$) of **5f**.

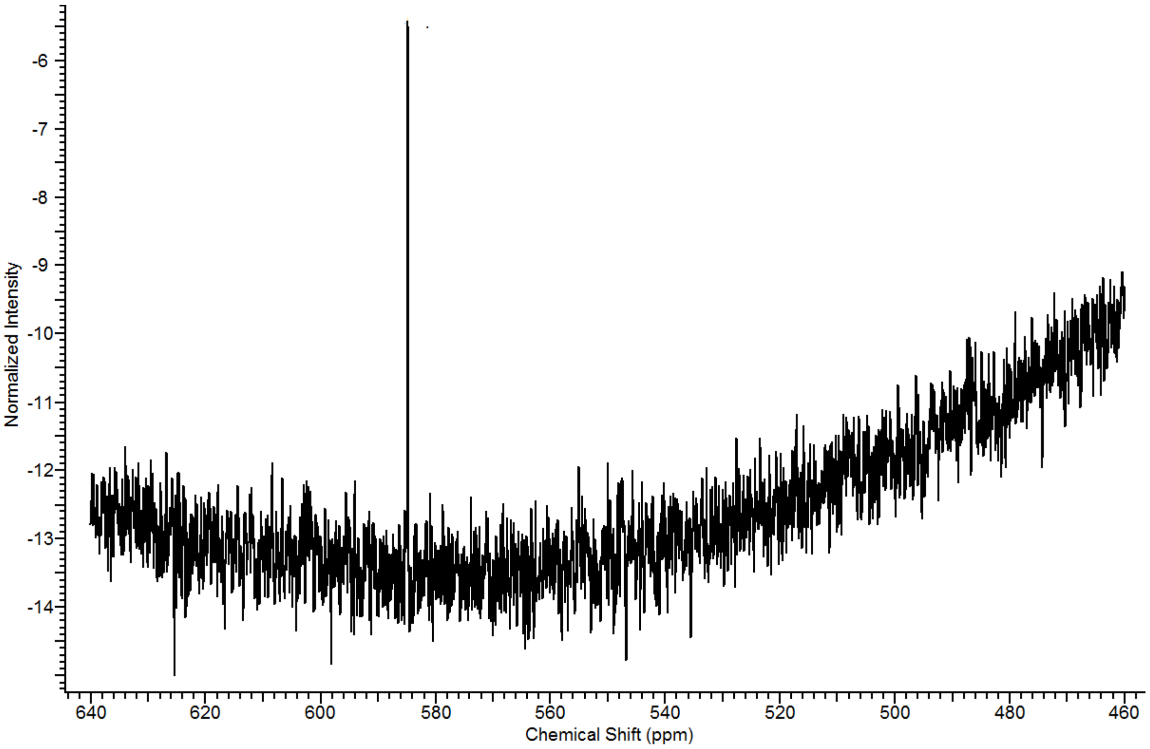

Figure S74. ^{1}H NMR spectrum (401 MHz, $CDCl_3$) of **5g**.

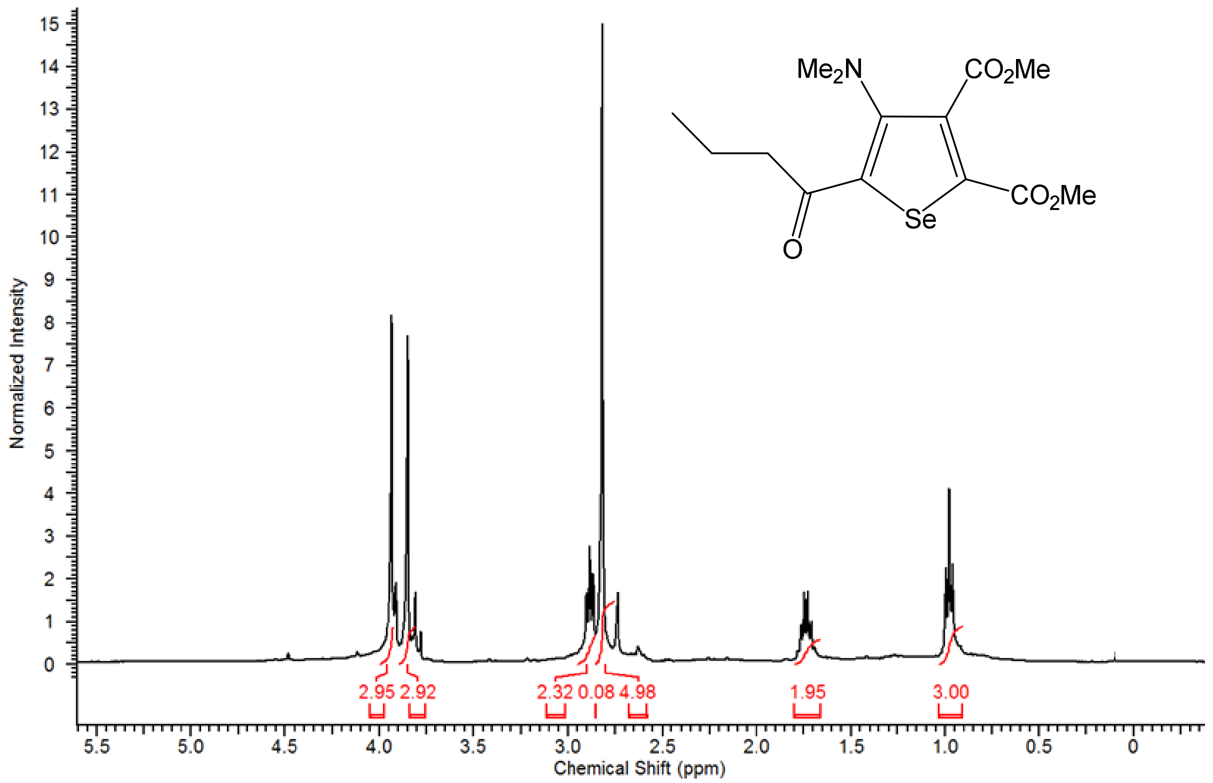

Figure S75. ^{13}C{^{1}H} NMR spectrum (101 MHz, $CDCl_3$) of **5g**.

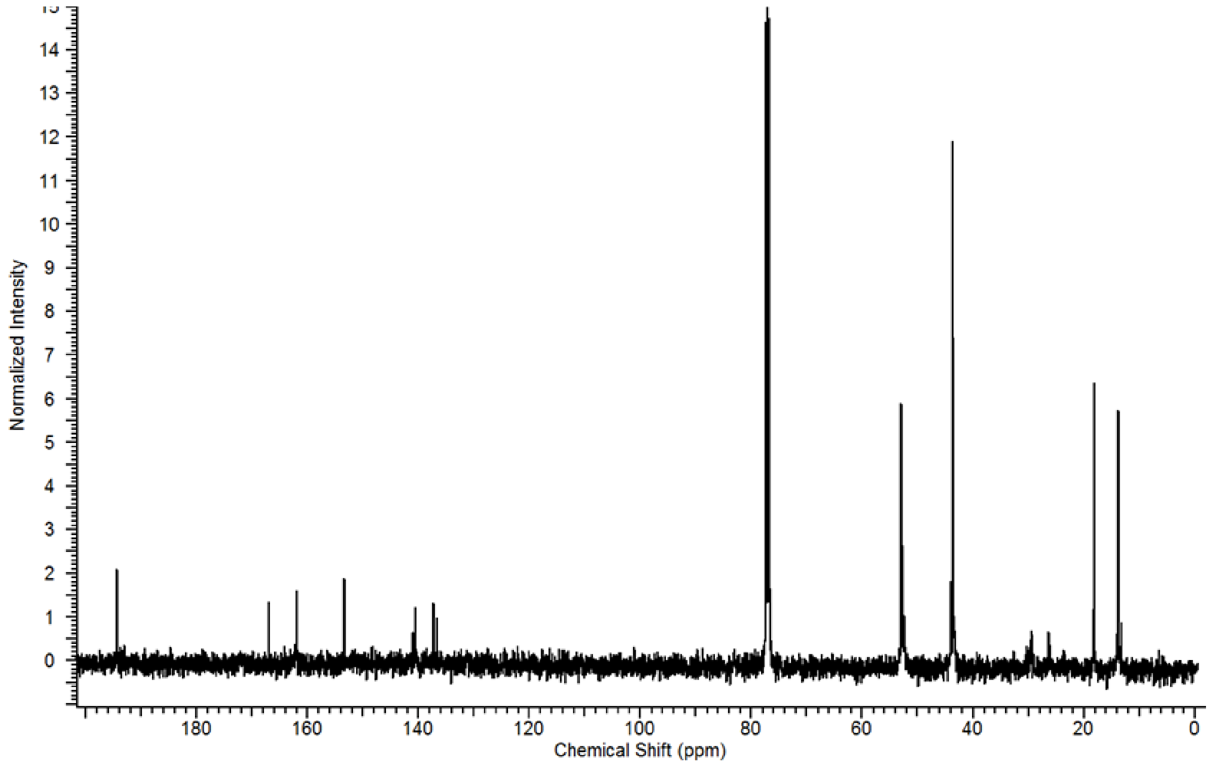

Figure S76. ^{77}Se NMR spectrum (76 MHz, $CDCl_3$) of **5g**.

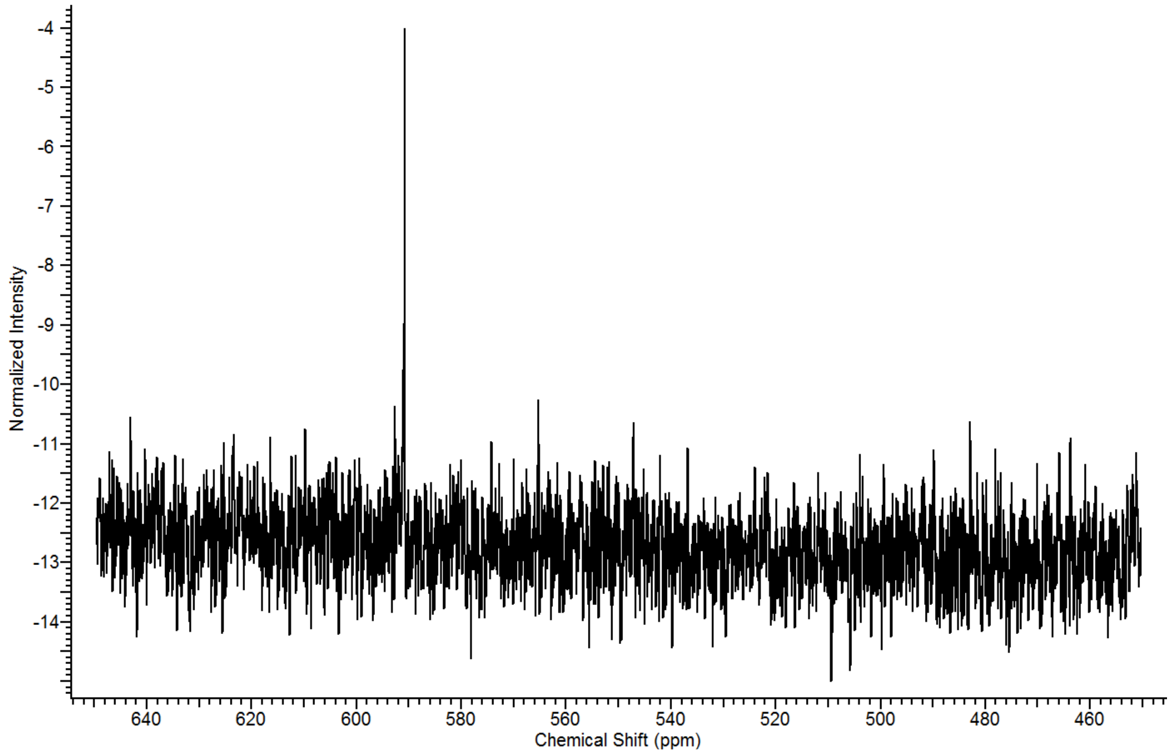

Figure S77. ^{1}H NMR spectrum (401 MHz, $CDCl_3$) of **5h**.

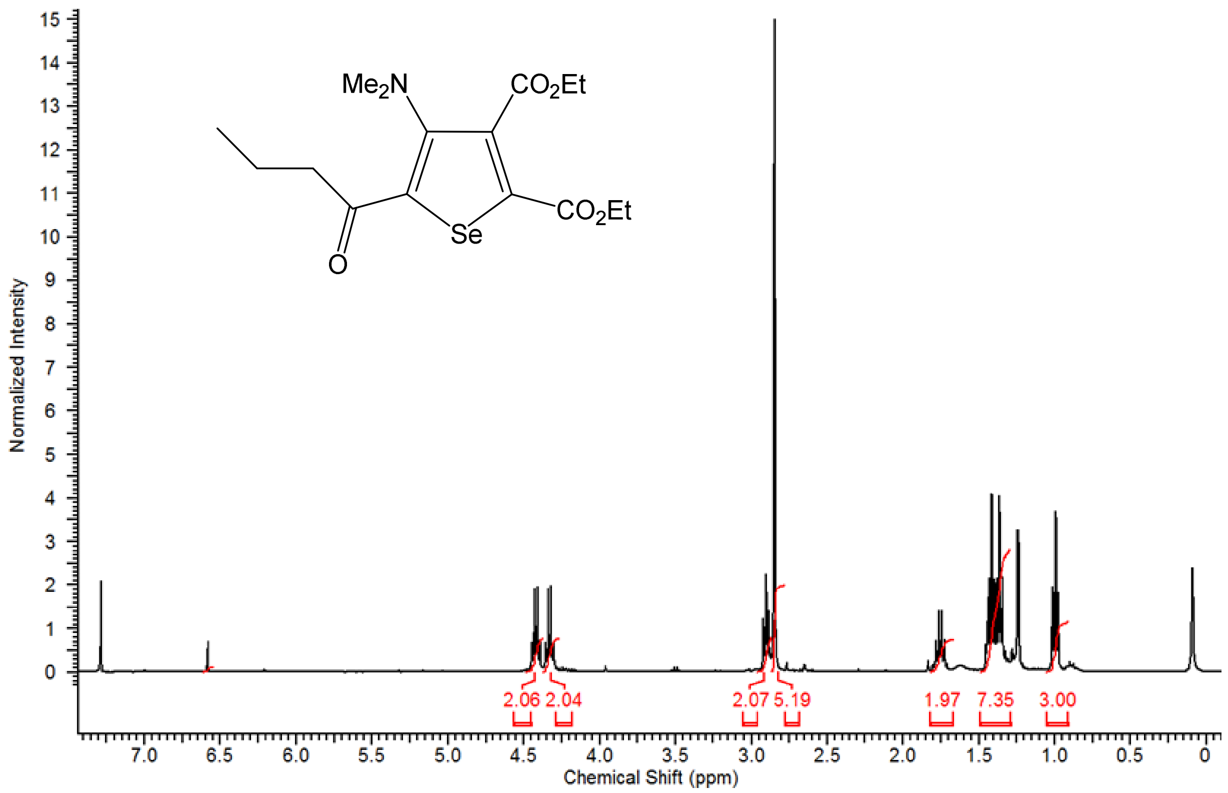

Figure S78. $^{13}C\{^{1}H\}$ NMR spectrum (101 MHz, $CDCl_3$) of **5h**.

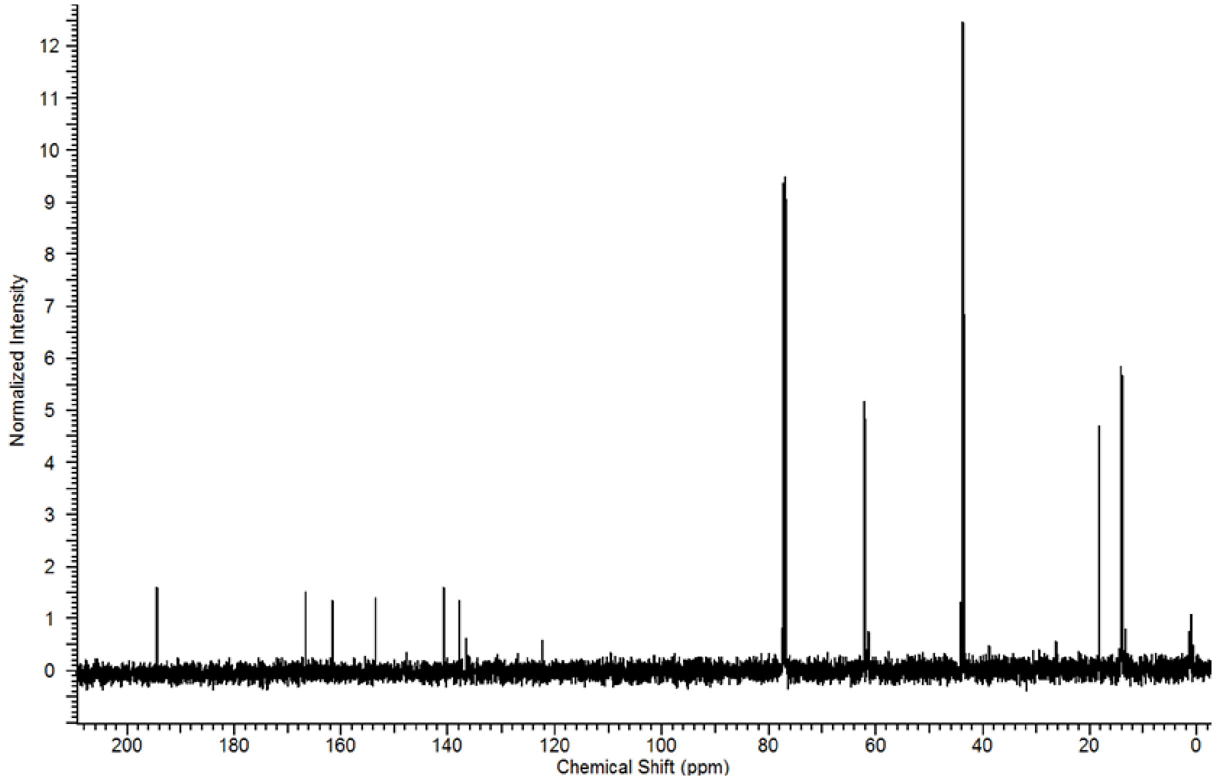

Figure S79. ^{77}Se NMR spectrum (76 MHz, $CDCl_3$) of **5h**.

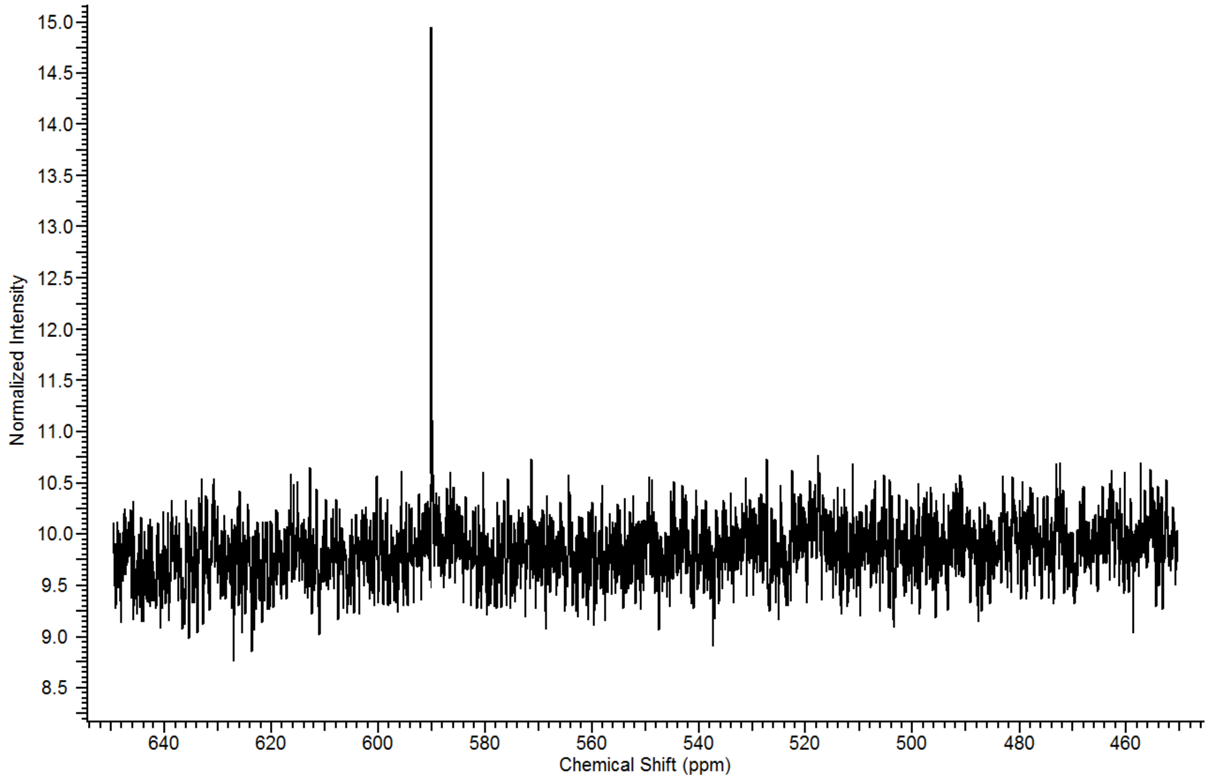

Figure S80. ^{1}H NMR spectrum (401 MHz, $CDCl_3$) of **5i**.

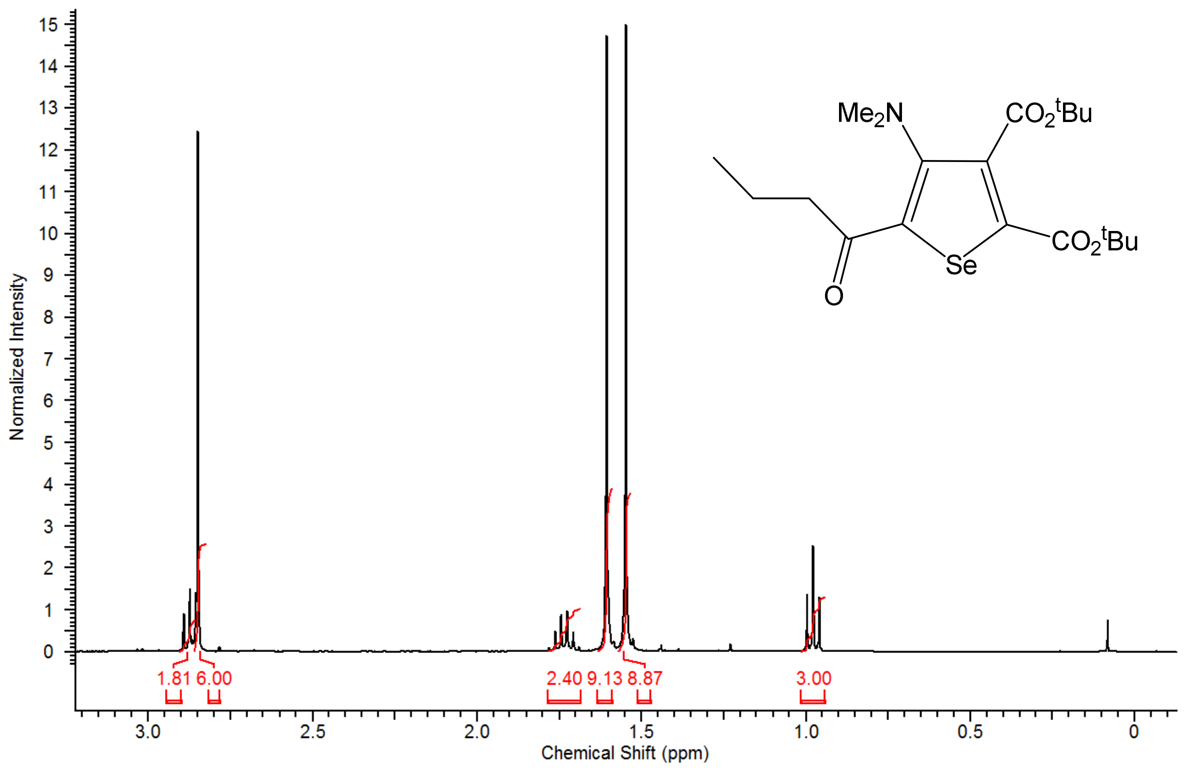

Figure S81. $^{13}C\{^{1}H\}$ NMR spectrum (101 MHz, $CDCl_3$) of **5i**.

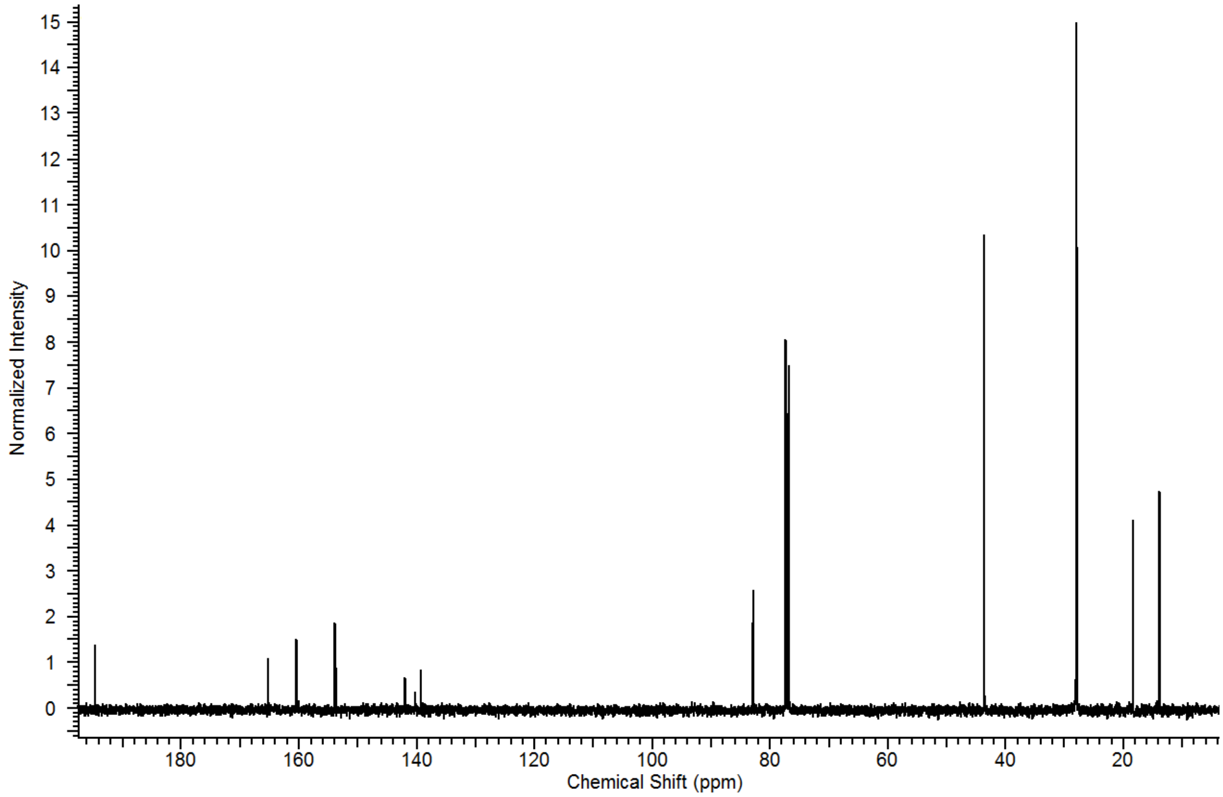

Figure S82. ^{77}Se NMR spectrum (76 MHz, $CDCl_3$) of **5i**.

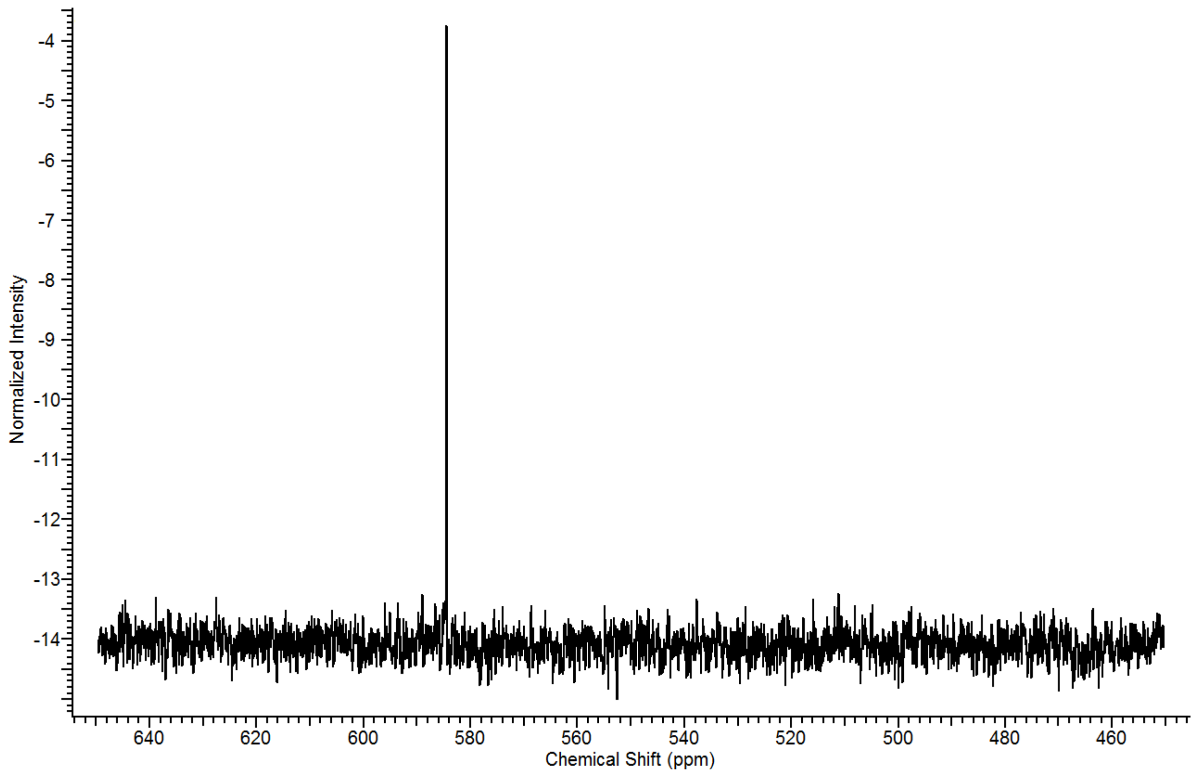

Figure S83. ^{1}H NMR spectrum (401 MHz, $CDCl_3$) of **5j**.

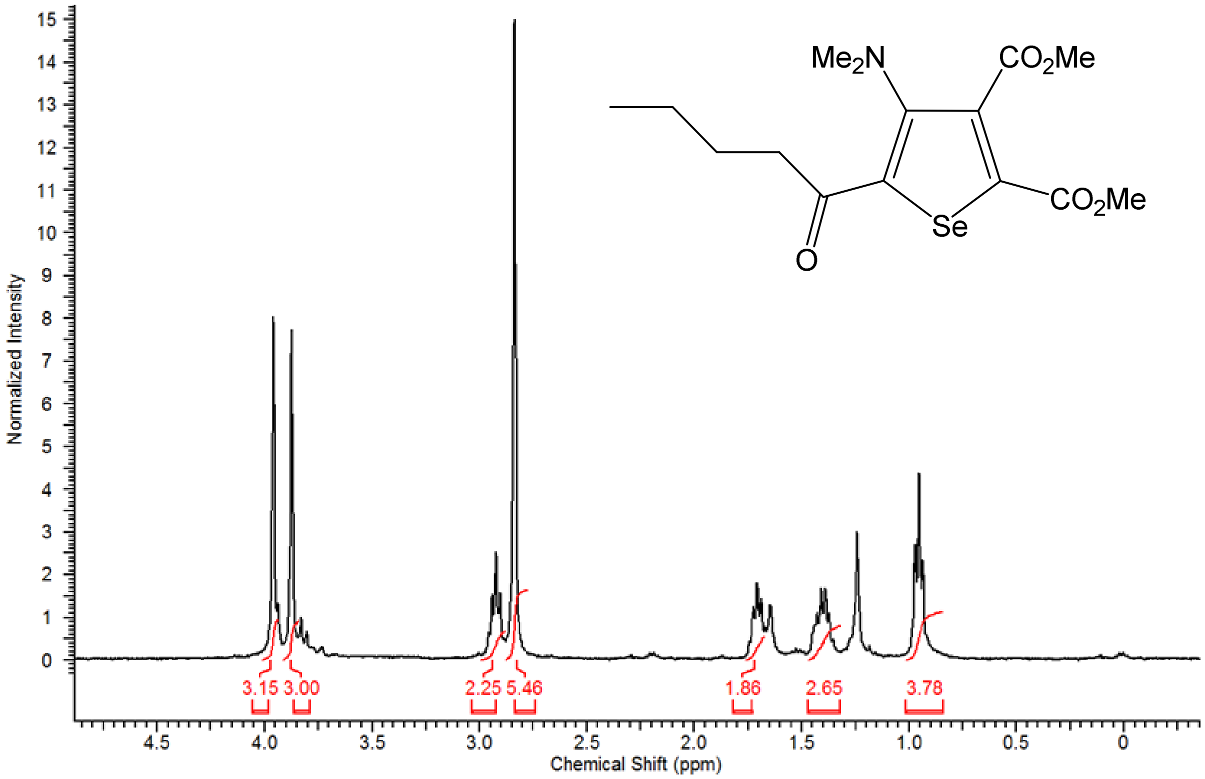

Figure S84. ^{13}C{^{1}H} NMR spectrum (101 MHz, $CDCl_3$) of **5j**.

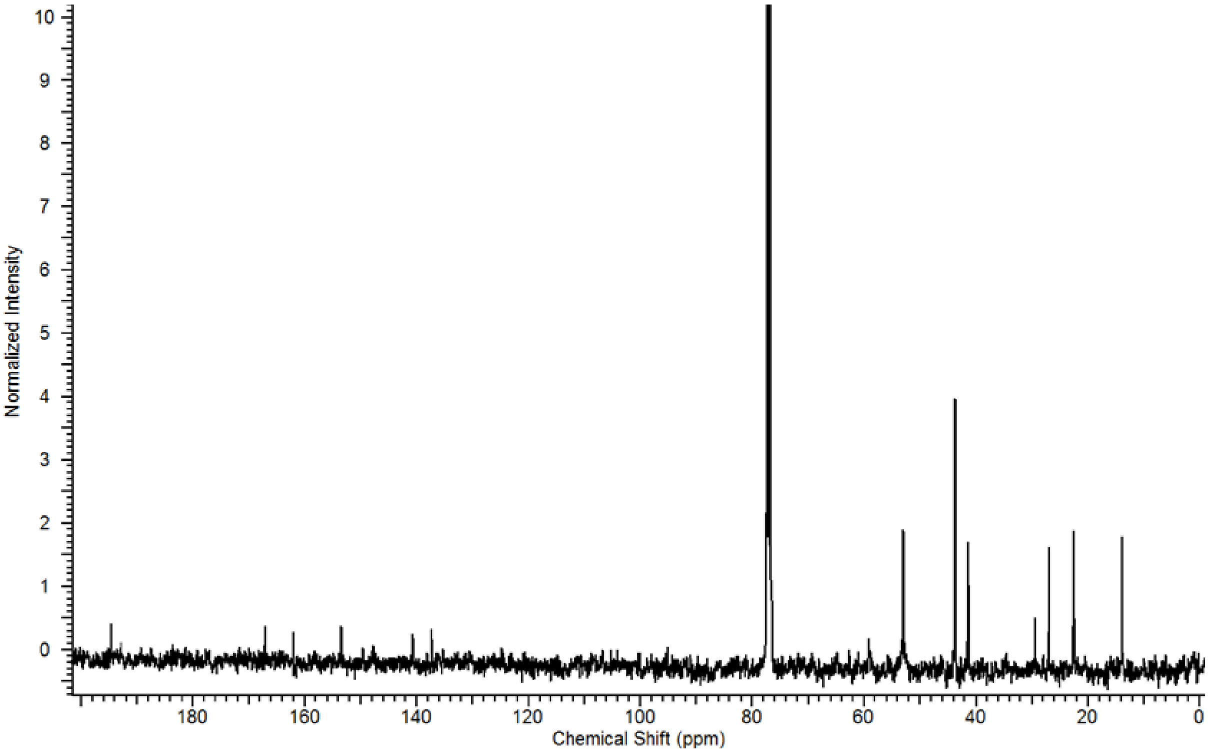

Figure S85. ^{77}Se NMR spectrum (76 MHz, $CDCl_3$) of **5j**.

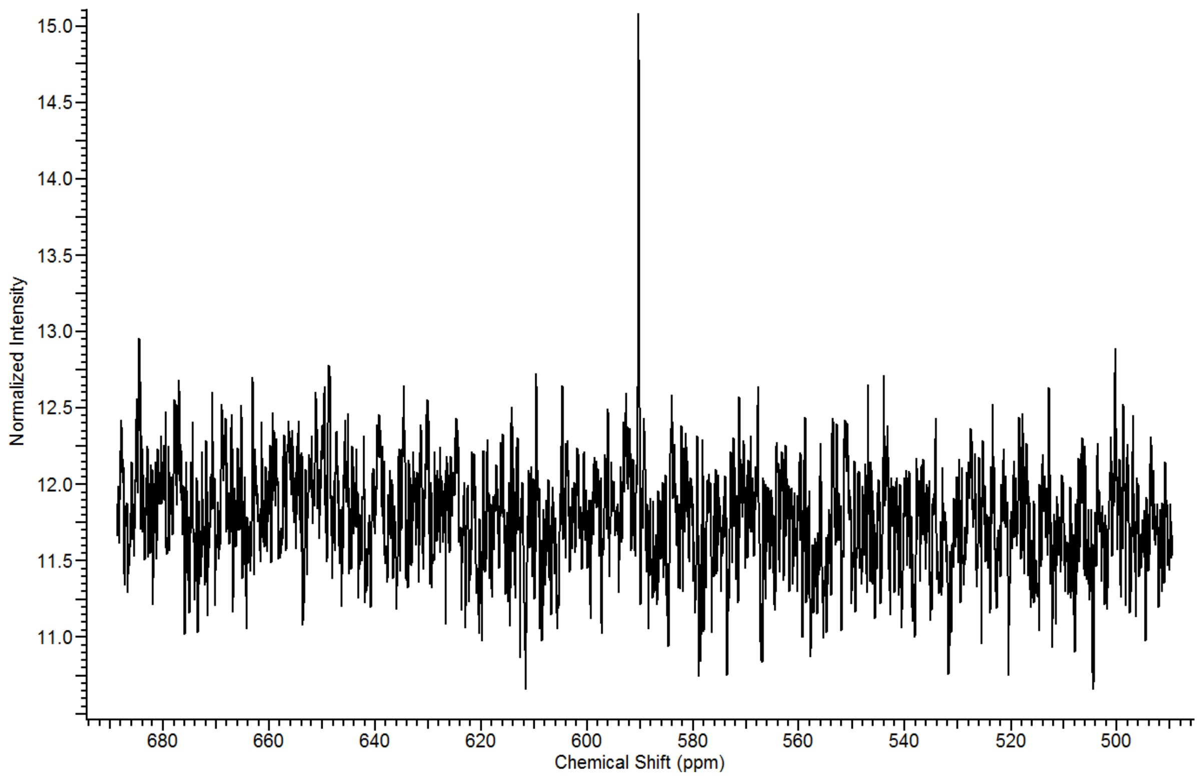

References

1 Albano, V. G.; Busetto, L.; Monari, M.; Zanotti, V. Reactions of acetonitrile di-iron m -aminocarbyne complexes; synthesis and structure of [Fe_2(μ-$CNMe_2$)(μ-H)$(CO)_2(Cp)_2$]. *J. Organomet. Chem.* **2000**, *606*, 163–168.

2 Ciancaleoni, G.; Zacchini, S.; Zanotti, V.; Marchetti, F. DFT Mechanistic Insights into the Alkyne Insertion Reaction Affording Diiron μ-Vinyliminium Complexes and New Functionalization Pathways. *Organometallics* **2018**, *37*, 3718−3731.

3 Albano, V. G.; Busetto, L.; Marchetti, F.; Monari, M.; Zacchini, S.; Zanotti, V. Diiron μ-Vinyliminium Complexes from Acetylene Insertion into a Metal−Aminocarbyne Bond. *Organometallics* **2003**, *22*, 1326-1331.

4 Busetto, L.; Marchetti, F.; Zacchini, S.; Zanotti, V. Unprecedented Zwitterionic Iminium−Chalcogenide Bridging Ligands in Diiron Complexes. *Organometallics* **2006**, *25*, 4808-4816.
